# Stronger Bounds for Weak Epsilon-Nets in Higher Dimensions

**DOI:** 10.1007/s00454-026-00878-y

**Published:** 2026-11-06

**Authors:** Natan Rubin

**Affiliations:** Institute for The Theory of Computing, The Stein Faculty of Computer and Information Science, Ben-Gurion University of the Negev, Beer-Sheba, Israel

**Keywords:** Epsilon-nets, Convex sets, Selection theorems, Simplices, Hypergraphs, Transversals

## Abstract

Given a finite point set *P* in $\mathbb {R}^d$, and ϵ>0 we say that $N\subseteq \mathbb {R}^d$ is a weak ϵ-net if it pierces every convex set *K* with $|K\cap P|\ge \epsilon |P|$. We show that for any finite point set in dimension d≥3, and any ϵ>0, one can construct a weak ϵ-net whose cardinality is $\displaystyle O^*\left( \frac{1}{\epsilon ^{2.558}}\right)$ in dimension d=3, and $\displaystyle o\left( \frac{1}{\epsilon ^{d-1/2}}\right)$ in all dimensions d≥4. (In the sequel, the $O^*(x)$ notation hides multiplicative factors that are bounded by $x^{\gamma }$, for arbitrary small γ>0, whereas the $\tilde{O}(x)$-notation hides multiplicative factors that are polylogarithmic in *x*.) To be precise, our weak ϵ-net has cardinality $\displaystyle O\left( \frac{1}{\epsilon ^{\alpha _d+\gamma }}\right)$ for any γ>0, with

$$ \alpha _d= {\left\{ \begin{array}{ll} 2.558 &  {\text {if}} \ d=3\\ 3.48 &  {\text {if}} \ d=4\\ \left( d+\sqrt{d^2-2d}\right) /2 &  {\text {if}} \ d\ge 5. \end{array}\right. } $$

This is the first significant improvement of the bound of $\displaystyle \tilde{O}\left( \frac{1}{\epsilon ^d}\right)$ that was obtained in 1993 by Chazelle, Edelsbrunner, Grigni, Guibas, Sharir, and Welzl for general point sets in dimension d≥3.

## Introduction

### Background

**Transversals and** ϵ**-nets.** Given a family ${{\mathcal {K}}}$ of geometric ranges in $\mathbb {R}^d$ (e.g., lines, triangles, or convex sets), we say that $N\subset \mathbb {R}^d$ is a *transversal* to ${{\mathcal {K}}}$ (or *N* pierces ${{\mathcal {K}}}$) if each range $K\in {{\mathcal {K}}}$ is pierced by at least one point of *N*. Given an underlying set *P* of *n* points, we say that a range $K\in {{\mathcal {K}}}$ is ϵ*-heavy* if $|P\cap K|\ge \epsilon n$. We say that *N* is an ϵ*-net* for ${{\mathcal {K}}}$ if it pierces every ϵ-heavy range in ${{\mathcal {K}}}$. We say that such a set *N* is a *strong* ϵ*-net* for ${{\mathcal {K}}}$ if N⊂P, that is, the points of the net are drawn from the underlying point set *P*. Otherwise (i.e., if *N* includes additional points outside *P*), we say that *N* is a *weak* ϵ*-net*.

The study of ϵ-nets was initiated by Vapnik and Chervonenkis [60], in the context of Statistical Learning Theory. Following a seminal paper of Haussler and Welzl [36], ϵ-nets play a central role in Discrete and Computational Geometry [42]. For example, bounds on ϵ-nets determine the performance of the best-known algorithms for the Minimum Hitting Set/Set Cover Problem in geometric hypergraphs [7, 15, 28, 32], and the transversal numbers of families of convex sets [3, 4, 6, 40].

Haussler and Welzl [36] proved in 1986 the existence of strong ϵ-nets of cardinality $O\left( \frac{1}{\epsilon }\log \frac{1}{\epsilon }\right)$ for families of simply-shaped, or semi-algebraic geometric ranges in *d*-space, for a fixed d>0 (e.g., lines, boxes, spheres, halfspaces, or simplices), by observing that their induced hypergraphs have a bounded Vapnik-Chervonenkis dimension (so called *VC-dimension*).^1^ While the bound is generally tight for set systems with a bounded VC-dimension [41], tight $o\left( \frac{1}{\epsilon }\log \frac{1}{\epsilon }\right)$-size constructions were discovered for discs in $\mathbb {R}^2$, halfplanes in $\mathbb {R}^2$ and halfspaces in $\mathbb {R}^3$ [28, 41, 46], and rectangles in $\mathbb {R}^2$ and boxes in $\mathbb {R}^3$ [7, 51]. We refer the reader to a recent state-of-the-art survey [49].

It had long been conjectured that all the “natural” geometric instances, that involve simply-shaped geometric ranges in a fixed-dimensional Euclidean space $\mathbb {R}^d$, admit a strong ϵ-net of cardinality O(1/ϵ). The conjecture was refuted, for the particular case of line ranges, in 2010 by Alon [2]. Pach and Tardos [51] subsequently demonstrated that the multiplicative term $\Theta \left( \log 1/\epsilon \right)$ is necessary for halfspaces in dimension higher than 3.

**Weak** ϵ**-nets for convex sets.** In sharp contrast to the case of simply-shaped ranges, no constructions of small-size strong ϵ-nets exist for general families of convex sets in $\mathbb {R}^d$, for d≥2. For example, given an underlying set of *n* points in convex position in $\mathbb {R}^2$, any strong ϵ-net with respect to convex ranges must include at least n-ϵn of the points. Nevertheless, Bárány, Füredi and Lovász [14] observed in 1990 that families of convex sets in $\mathbb {R}^2$ still admit weak ϵ-nets of cardinality $O(\epsilon ^{-1026})$. Alon, Bárány, Füredi, and Kleitman [1] were the first to show in 1992 that families of convex sets in any dimension d≥1 admit weak ϵ-nets whose cardinality is bounded in terms of 1/ϵ and *d*. The subsequent study and application of weak ϵ-nets bear strong relations to convex geometry, including Helly-type, Tverberg-type, and Selection Theorems; see [43, Sects. 8–10] for a comprehensive introduction.

**Weak** ϵ**-nets and the Hadwiger-Debrunner Problem.** Alon and Kleitman [6] used the boundedness of weak ϵ-nets to confirm the long-standing (*p*, *q*)*-conjecture* by Hadwiger and Debrunner [34]. We say that a family ${{\mathcal {K}}}$ of convex sets satisfies the (*p*, *q*)*-property* if any its *p*-size subfamily ${{\mathcal {K}}}'\subset {{\mathcal {K}}}$ contains a *q*-size subset ${{\mathcal {K}}}''\subset {{\mathcal {K}}}$ with a non-empty common intersection $\bigcap {{\mathcal {K}}}''\ne \emptyset$. Hadwiger and Debrunner conjectured that for every positive integers *p*, *q* and *d* that satisfy $p\ge q\ge d+1$, there exists an integer $C_d(p,q)<\infty$ such that the following statement holds: *Any family* ${{\mathcal {K}}}$ *of convex sets in* $\mathbb {R}^d$ *with the* (*p*, *q*)-*property admits a transversal by at most* $C_d(p,q)$ *points.* Note that the celebrated Helly Theorem yields a transversal by a *single* point in the case p=q=d+1 whenever $|{{\mathcal {K}}}|\ge d+1$.

Showing good quantitative estimates for the Hadwiger-Debrunner numbers $C_d(p,q)$ is a formidable open problem which requires tight asymptotic bounds for weak ϵ-nets; see the latest study by Keller, Smorodinsky and Tardos [40], and the concluding discussion in Sect. 7. Very recently, lower bounds for several of the above questions – including strong and weak ϵ-nets with respect to line ranges [13], and the 2-dimensional Hadwiger-Debrunner numbers $C_2(p,q)$ [39] – were improved using the novel combinatorial machinery of hypergraph containers [12, 56].

**Point-selection theorems.** The aforementioned first general construction of weak ϵ-nets in the Euclidean spaces $\mathbb {R}^d$ of dimension d≥2, due to Alon et al. [1], used the following property of simplicial hypergraphs: for any *n* point set *P* in general position in $\mathbb {R}^d$ (with n≥d+1), and any dense collection *E* of *d*-dimensional simplices over *P*, which are identified with a subset of Pd+1, there is a point $x\in \mathbb {R}^d$ piercing a significant fraction of the simplices of *E*. (This fraction is polynomial in the edge density $|E|/{n\atopwithdelims ()d+1}$. The asymptotic guarantee was dramatically improved by the author [54], partly using the methods that are developed in this paper.) This deep result, which has since become known as *The Second Selection Theorem*, underlies a number of other fundamental results in discrete and computational geometry, such as the best-known estimates for *k*-sets and *k*-levels in dimension d≥5 [14]. Its special case $E={P\atopwithdelims ()d+1}$ is known as *The First Selection Theorem*.

**Bounds on weak** ϵ**-nets.** For any ϵ>0 and d≥0, let $f_d(\epsilon )$ be the smallest number f>0 so that, for any underlying finite point set *P*, one can pierce all the ϵ-heavy convex sets using only *f* points in $\mathbb {R}^d$. It is an outstanding open problem in Discrete and Computational geometry to determine the true asymptotic behaviour of $f_d(\epsilon )$ in dimensions d≥2. As Alon, Kalai, Matoušek, and Meshulam noted in 2001: “*Finding the correct estimates for weak* ϵ-*nets is, in our opinion, one of the truly important open problems in combinatorial geometry”* [4].

Alon, Bárány, Füredi, and Kleitman [1] (see also [6]) showed that $f_d(\epsilon )=O\left( 1/\epsilon ^{(d+1)(1-1/\beta _d)}\right)$, where $0<\beta _d<1$ denotes the so called *point selection exponent*, to be defined in the sequel. Using a different argument, they showed that $f_2(\epsilon )=O\left( 1/\epsilon ^2\right)$. The bound in higher dimensions d≥3 has been subsequently improved in 1993 by Chazelle et al. [24] to roughly $\displaystyle \tilde{O}(1/\epsilon ^d)$. Though the latter construction was somewhat simplified in 2004 by Matoušek and Wagner [45] using simplicial partitions with low hyperplane-crossing number [44], no study in the subsequent 25 years came close to tackling the notorious “$\displaystyle \frac{1}{\epsilon ^d}$-barrier” for general families of convex sets and arbitrary finite point sets in the Euclidean spaces $\mathbb {R}^d$ of (fixed) dimension d≥2.

In view of the best known lower bound of $\Omega \left( \frac{1}{\epsilon } \log ^{d-1}\left( \frac{1}{\epsilon }\right) \right)$ for $f_d(\epsilon )$ due to Bukh, Matoušek and Nivasch [17], it still remains to settle whether the asymptotic behaviour of this quantity substantially deviates from the long-known “almost-(1/ϵ)” bounds on strong ϵ-nets (e.g., for lines and triangles in $\mathbb {R}^2$ or simplices in $\mathbb {R}^d$)? The only interesting instances in which the gap has been nearly closed, involve essentially lower-dimensional distributions of points [5, 18, 24]. For example, Alon, Kaplan, Nivasch, Sharir, and Smorodinsky [5] showed in 2008 that any finite point set in *convex position in* $\mathbb {R}^2$ allows for a weak ϵ-net of cardinality $\displaystyle O\left( g(\epsilon )/\epsilon \right)$ with respect to convex sets, where g(·) denotes the very slowly inverse Ackerman function.

A previous study by the author [53] made the first step to breaching the infamous $\displaystyle \frac{1}{\epsilon ^d}$-barrier by showing that $\displaystyle f_2(\epsilon )=O\left( \frac{1}{\epsilon ^{3/2+\gamma }}\right)$, for any constant γ>0. Unfortunately, its (partly) ad-hoc machinery did not directly apply to dimensions d≥3.

### Our Result

The present study offers the first $o(1/\epsilon ^d)$-size construction in the Euclidean spaces $\mathbb {R}^d$ of dimension d≥3.^2^

#### Theorem 1.1

Let d≥3, and

1 $$\begin{aligned} \alpha _d:= {\left\{ \begin{array}{ll} 2.558 &  {\text {if}} \ d=3\\ 3.48 &  {\text {if}} \ d=4\\ \left( d+\sqrt{d^2-2d}\right) /2 &  {\text {if}} \ d\ge 5. \end{array}\right. } \end{aligned}$$

Then we have that $\displaystyle f_d(\epsilon )=O\left( \displaystyle \frac{1}{\epsilon ^{\alpha _d+\gamma }}\right)$ for any γ>0. That is, for any underlying set *P* of *n* points in $\mathbb {R}^d$, and any ϵ>0, one can construct a weak ϵ-net with respect to convex sets, whose cardinality is $O\left( \displaystyle \frac{1}{\epsilon ^{\alpha _d+\gamma }}\right)$, for any γ>0.^3^

In particular, we have that $f_3(\epsilon )=O^*\left( 1/\epsilon ^{2.558}\right)$, and $f_d(\epsilon )=o\left( 1/\epsilon ^{d-1/2}\right)$ for any d≥4.

**Proof overview.** Our proof of Theorem 1.1 is fully constructive. Similar to a plethora of previous works [24, 45, 53], it uses standard divide-and-conquer methods (e.g., Matoušek’s simplicial partitions [44]) to derive an efficient recurrence in ϵ>0 (and, implicitly, the ground set *P*).

**Fig. 1:**
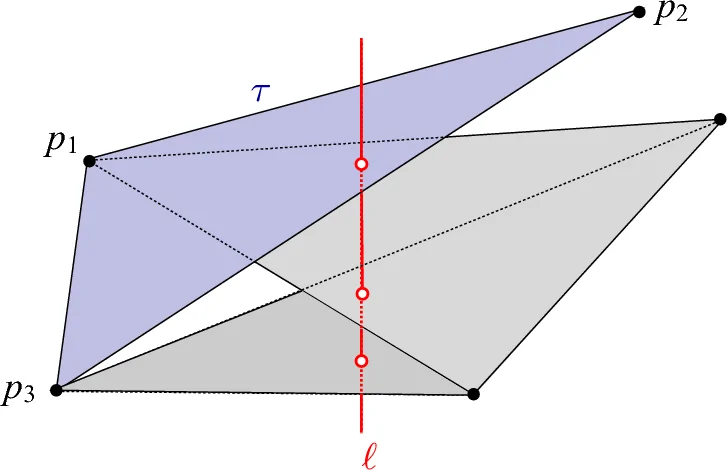
Illustrating the proof of Theorem 1.1 in $\mathbb {R}^3$. For each of the strong 1-dimensional nets that constitute our weak ϵ-net, the ground set $X_\ell$ is contained in a vertical line ℓ, and it is comprised of the ℓ-intercepts $\tau \cap \ell$ of certain triangles $\tau =\triangle p_1p_2p_3$, with $\{p_1,p_2,p_3\}\in {P\atopwithdelims ()3}$

However, at the heart of our new estimate lies a rather basic reduction of the weak ϵ-net problem in $\mathbb {R}^d$ to a sequence of non-recursive 1*-dimensional* strong ν-nets $N_\ell$ (with respect to intervals) for $\nu \gg \epsilon ^d$, which are restricted to a handful of vertical lines ℓ within $\mathbb {R}^d$. For each of these 1-dimensional instances, its respective ground set $X_\ell$ will be comprised of the ℓ-intercepts $\tau \cap \ell$ of certain (d-1)-dimensional simplices τ, each spanned by some *d*-tuple of points within *P*. See Fig. 1 for an illustration in dimension d=3.

For the sake of simplicity, every ϵ-heavy convex set *K* will be assigned a unique subset $P_K\subseteq P\cap K$ of exactly $\lceil \epsilon n\rceil$ points which determine ${\lceil \epsilon n\rceil \atopwithdelims ()d}$ (d-1)-dimensional simplices. In what follows, we establish a novel *line-selection* result, which yields a small family $\mathcal {{L}}$ of vertical lines ℓ that would pierce a large fraction of the (d-1)-dimensional simplices induced by an *unknown* such subset of $\lceil \epsilon n\rceil$ points of *P*. Hence, a “typical” ϵ-heavy convex set *K* must induce, for some $\ell \in \mathcal {{L}}$, an interval $K\cap \ell$ that contains $\Omega ^*\left( (\epsilon n)^d\right)$ points of $X_\ell$.

As is shown in the sequel, any 1-dimensional reduction of this sort must leave out some sub-family of “narrow” sets *K* whose (d-1)-dimensional simplices are hard to intercept by few vertical lines. Fortunately, such “narrow” sets *K* can be passed on to subsidiary recursive instances which entail smaller sets in $\mathbb {R}^d$, and larger fractions ϵ>0.

More importantly, though, a naive implementation of this scheme would yield ground sets $X_\ell \subset \ell$ of cardinality $\Theta \left( n^d\right)$ for certain lines $\ell \in \mathcal {{L}}$, which would result in ratios $\nu \approx (\epsilon n)^d/|X_\ell |\approx \epsilon ^d$ and lead to 1-dimensional ν-nets $N_\ell$ of cardinality $\Omega (1/\nu )=\Omega \left( 1/\epsilon ^d\right)$. To obtain an $o(1/\epsilon ^d)$-size net, the ϵ-heavy convex sets *K* will be subdivided into several categories based on the relative distribution of the points of $P_K$ within the simplicial partition of the entire ground set *P* [44].

Specializing to a certain class of “hyperplane-like” convex sets, however, our 1-dimensional reduction can be attained with $\nu \approx \epsilon ^{\alpha _d}$. To this end, each 1-dimensional set $X_\ell$ will be replaced with a sparser substitute $Y_\ell \subseteq X_\ell$ of $O^*\left( \epsilon ^{d-\alpha _d}n^d\right)$ points with the following property: any relatively flat convex set *K* still contains almost $\epsilon ^dn^d$ elements of $\bigcup _{\ell \in \mathcal {{L}}}Y_\ell$.

The remaining ϵ-heavy convex sets will be pierced using an ad-hoc combination of more specialized (and mostly non-recursive) nets, each tailored to a particular class of convex sets. For example, the relatively “thick” convex sets will be pierced by a variant of 1-dimensional $\Omega ^*(\epsilon ^{\alpha _d})$-nets with respect to a differently chosen point set within $\bigcup \mathcal {{L}}$, and an additional strong $\Omega ^*\left( \epsilon ^{\alpha _d}\right)$-net will be used to pierce all convex sets that contain “heavy” convex polytopes of bounded description complexity.

An especially challenging scenario, which notably does not occur in $\mathbb {R}^2$, involves the ϵ-heavy convex sets *K* for which the projection $P_K^\perp$ is in “almost convex” position within $\mathbb {R}^{d-1}$; in what follows such subsets $P_K$ will be referred to as δ*-hollow*. Our separate divide-and-conquer treatment of this class of convex sets will involve an ad-hoc cutting [21, 25, 26] in a certain arrangement of hyperplanes which support (d-1)-dimensional simplices of Pd. The subsequent analysis of the resulting (sub)-recurrence in ϵ will use upper bounds on the combined complexity of multiple cells in a hyperplane arrangement [8, 9].

### Paper Organization

In Sect. 2 we introduce the essential geometric machinery, which includes (i) configurations of point sets, (ii) simplicial hypergraphs and point-selection theorems, (iii) Matoušek’s simplicial partitions, (iv) random sampling and strong ϵ-nets, and (v) hyperplane arrangements. In addition, we lay down the recursive framework for bounding the quantities $f_d(\epsilon )$, and provide a more comprehensive roadmap to the entire proof of Theorem 1.1.

In Sect. 3 we obtain the cornerstone reduction of the weak ϵ-net problem to a sequence of 1-dimensional ν-nets.

In Sect. 4 we establish Theorem 1.1 by fixing the underlying set *P* of *n* points, and then describing a small-size net that pierces all the ϵ-heavy convex sets *K*. To this end, we analyze the implicit structure of the subsets of *P* that are “cut out” by the ϵ-heavy convex sets *K*, with particular emphasis on certain families of simplices that are determined by such subsets of $\lceil \epsilon n\rceil$ points. As a result, the convex sets under our consideration are subdivided into finer sub-categories, to be dispatched by separate nets whose details are relegated to Sects. 5 and 6. Namely, in Sect. 5, we show how to pierce the convex sets *K* that determine (so called) δ*-punctured* subsets $P_K$ of $\lceil \epsilon n\rceil$ points within *P*, whereas in Sect. 6 we pierce the remaining ϵ-heavy convex sets, which yield δ*-hollow* subsets within *P*.

In Sect. 7, we sum-up the properties of our improved weak ϵ-net construction, and outline a few promising directions for further investigation.

## Preliminaries

### Basic Notation

**Vinogradov notation.** For any $x,y\in \mathbb {R}$, we denote x≪y (resp., x≫y) whenever x=O(y) (resp., x=Ω(y)).

**Set families and random sampling.** Let *A* be a set of finite cardinality *n*, and r≥0 and integer. In the sequel, we use Ar to denote the collection of all the *r*-size subsets of *A*. (This collection is empty if n<r.) We then use ${A\atopwithdelims ()\le r}$ to denote the collection of all such subsets of *A* whose cardinality is at most *r*.

Now let *r* be an integer that satisifes $1\le r\le n=|A|$. An *r-sample* of *A* is a random subset $B\in {A\atopwithdelims ()r}$ of *r* elements, which is selected with uniform probability $1/{n\atopwithdelims ()r}$ from among all such subsets within Ar. The reader is referred to Appendix A for further properties of *r*-samples, and their application to finding small-size strong ϵ-nets for set systems of bounded VC-dimension.

**Convex hulls and affine closures.** In the sequel, we use ${{\texttt{conv}}}(A)$ to denote the convex hull of any point set $A\subseteq \mathbb {R}^d$, and we use ${{\texttt{aff}}}(A)$ to denote the *affine closure* of *A* – the smallest affine space (i.e., a translate of a linear subspace of $\mathbb {R}^d$) that contains *A*. We say that a finite point set $A\subset \mathbb {R}^d$ is in *convex position* if every point of *A* lies on the relative boundary of ${{\texttt{conv}}}\left( A\right)$.

**Projections.** For any point $x=(x_1,\ldots ,x_d)\in \mathbb {R}^d$, and any set $A\subseteq \mathbb {R}^d$, we denote their vertical projections by $x^\perp :=(x_1,\ldots ,x_{d-1})$ and $A^\perp :=\{x^\perp \mid x\in A\}$.

Conversely, for any point $y\in \mathbb {R}^{d-1}$, and any set $B\subseteq \mathbb {R}^{d-1}$, we use $y^*$ to denote the vertical line $\{x\mid x^\perp =x\}$ *in* $\mathbb {R}^d$ over *y*, and we use $B^*$ to denote the vertical prism $\bigcup \{y^*\mid y\in B\}$ over *B*. (With some abuse of notation, these definitions will also extend to points and sets in $\mathbb {R}^{d}$. For example, for any point $x\in \mathbb {R}^d$, we will use $x^*$ to denote the vertical line $\left( x^\perp \right) ^*$ through *x* in $\mathbb {R}^d$.)

We say that a set $A\subseteq \mathbb {R}^d$ lies *above* another set $B\subseteq \mathbb {R}^d$ (whereas *B* lies *below* *A*) if (i) we have that either $A^\perp \subseteq B^\perp$ or $A^\perp \supseteq B^\perp$, and (ii) for any vertical line ℓ, that intersects both sets, the interval $\ell \cap A$ lies entirely above $\ell \cap B$. (Notice that the described partial relation between sets in $\mathbb {R}^d$ is not transitive, as can be demonstrated, e.g., by a cycle of just three line segments in $\mathbb {R}^3$. Economical cutting of cycles in this partial vertical order in $\mathbb {R}^3$ is an active area of discrete and computational geometry [11].)

*k***-flats.** In what follows, we refer to any *k*-dimensional affine space as a *k-dimensional plane*, or a *k-flat*; the (d-1)-dimensional planes in $\mathbb {R}^d$ are called *hyperplanes*.

In the sequel, we say that a *k*-flat *g*, for $1\le k\le d-1$, *crosses* a convex set *K* if *g* intersects the relative interior of *K* yet does not contain *K*.

**General position.** To simplify the exposition, we assume in what follows, that the underlying point set *P* in Theorem 1.1 is in *a general position* in $\mathbb {R}^d$ (as described, e.g., [43, Sect. 5] and [30]). In particular, no d+1 of them lie on the same hyperplane, and no *d* of them determine a hyperplane that is parallel to any of the coordinate axes; hence, no *d* of the vertically projected points lie in the same (d-2)-flat within $\mathbb {R}^{d-1}$. Furthermore, the affine hull ${{\texttt{aff}}}(A)$ of any subset A⊆P of at most d+1 points must be an (|A|-1)-flat. More generally, for any *k* pairwise disjoint subsets $A_1,\ldots ,A_k\subseteq P$, each of cardinality $|A_i|\le d+1$, the intersection $\bigcap _{i=1}^d {{\texttt{aff}}}(A_i)$ is an affine space of dimension $\max \{-1,d-\sum _{i=1}^l (d-|A_i|+1)\}$ (where -1 is the dimension of the empty set), and the analogous property should hold, in $\mathbb {R}^{d-1}$, for the vertically projected set $P^\perp$.

A standard perturbation argument [30] shows that these assumptions incur no loss of generality: a weak ϵ-net with respect to an infinetisimately perturbed point set, now in a general position, would immediately yield such a net with respect to the original set.

**Simplices.** Let *A* be a finite point set in general position in $\mathbb {R}^d$. The elements of ${A\atopwithdelims ()\le d+1}$ are called *simplices*. Specifically, for $0\le k\le d$, every (k+1)-size set $S\in {A\atopwithdelims ()k+1}$ determines a *k-dimensional simplex* ${{\texttt{conv}}}(S)$ (or, simply, a *k-simplex*) which supports the *k*-flat ${{\texttt{aff}}}(S)$; see [43, Sects. 1 and 5]. For the sake of brevity, the induced set of |A|k+1 *k*-dimensional simplices will be identified with Ak+1.^4^ The 1-dimensional simplices of A2 will be called 2*-edges*.

### Configurations of Point Sets and Hyperplanes in $\mathbb {R}^d$

**Fig. 2:**
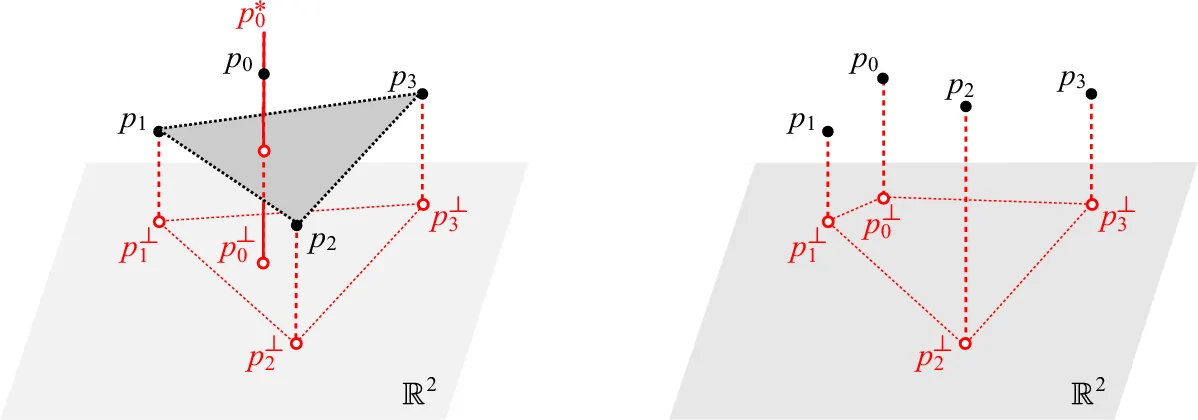
Left: The set $A=\{p_0,p_1,p_2,p_3\}$ in $\mathbb {R}^3$ is punctured, as the vertical line $p_0^*$ through $p_0$ crosses ${{\texttt{conv}}}(p_1,p_2,p_3)$. Right: The set $A=\{p_0,p_1,p_2,p_3\}$ in $\mathbb {R}^3$ is hollow

δ**-punctured and** δ**-hollow point sets.** We say that a set *A* of d+1 points in general position is *punctured* if a vertical line $a^*$ through some point a∈A crosses the relative interior of ${{\texttt{conv}}}(A\setminus \{a\})$; see Fig. 2. If such a set *A* is not punctured, we say it is *hollow*, in which case the vertical projection $A^\perp$ of such a set *A* must be in convex position within $\mathbb {R}^{d-1}$; see Fig. 2 (right). (It is instructive to note that *all* triples points in general position in $\mathbb {R}^2$ are punctured.)

More generally, for $\delta \ge 0$, we say that a finite set *A* in general position in $\mathbb {R}^d$ is δ*-punctured* if it encompasses more than $\delta {|A|\atopwithdelims ()d+1}$ punctured subsets $B\in {A\atopwithdelims ()d+1}$, and that it is δ*-hollow* otherwise (in which case *A* encompasses at least $(1-\delta ){|A|\atopwithdelims ()d+1}$ hollow subsets $B\in {A\atopwithdelims ()d+1}$). In particular, this point set *A* is 0-hollow if and only if its projection $A^\perp$ is in convex position in $\mathbb {R}^d$. In Appendix B, the probabilistic argument of Clarkson and Shor [27] is used to show that any δ-hollow point set contains a relatively large 0-hollow subset.

#### Lemma 2.1

For every dimension d≥2 there is a constant $c_0=c_0(d)>0$ with the following property:

Let *A* be a δ-hollow set of *m* points in $\mathbb {R}^d$, and S⊆A be an *r*-sample of *A*, so that $1\le r\le m$ and $\delta \le c_0/r^{d+1}$. Then *S* is 0-hollow with probability at least 99/100.

**Separated families of convex sets.** We say that a finite family $\Sigma =\{\Delta _1,\ldots ,\Delta _m\}$ of m≥d+1 compact convex sets in $\mathbb {R}^d$ is *separated* if no d+1 among its elements are intersected by the same hyperplane [19, 52, 54].

**Fig. 3:**
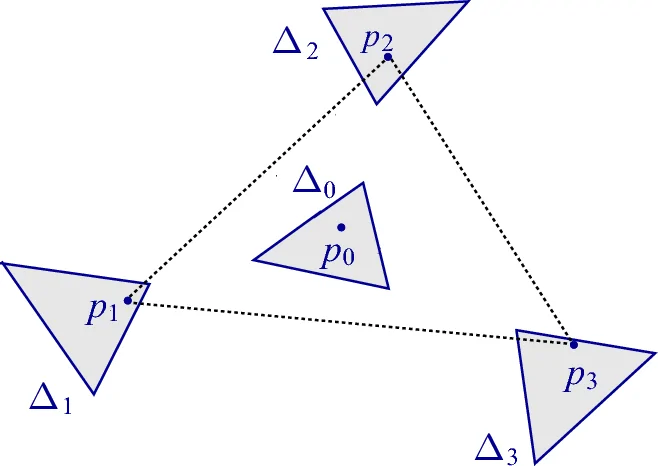
The set $\Delta _0$ is surrounded by the three other sets $\Delta _1,\Delta _2$ and $\Delta _3$ in $\mathbb {R}^2$. For any choice of $p_0\in P_0,p_1\in \Delta _1,p_2\in \Delta _2$, and $p_3\in \Delta _3$, the point $p_0$ lies in the interior of ${{\texttt{conv}}}(p_1,p_2,p_3)$

**Surrounded subsets of points.** We say that a compact (yet not necessarily finite, or convex) set $\Delta _0$ in $\mathbb {R}^d$ is *surrounded* by d+1 compact sets $\Delta _1,\ldots ,\Delta _{d+1}\subseteq \mathbb {R}^{d}$ if for any choice $p_0\in \Delta _0,p_1\in \Delta _1,p_2\in \Delta _2,\ldots ,p_{d+1}\in \Delta _{d+1}$, the point $p_0$ lies in the interior of ${{\texttt{conv}}}(p_1,\ldots ,p_{d+1})$. See Fig. 3. In particular, no d+1 points in such a (d+2)-size sequence $p_0,\ldots ,p_{d+1}$ can lie in the same hyperplane, so the (d+2)-size family $\Sigma =\{\Delta _0,\ldots ,\Delta _{d+1}\}$ is separated.

In Appendix B, we use elementary continuous motion arguments to establish the following properties.

#### Lemma 2.2

**Fig. 4:**
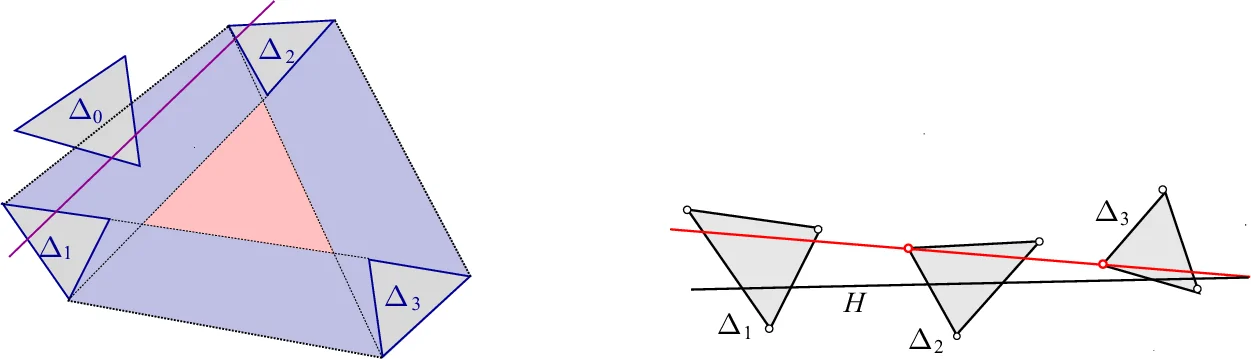
Left: Lemma 2.2 in $\mathbb {R}^2$ – the compact convex set $\Delta _0$ intersects the convex hull of $\bigcup _{i=1}^3\Delta _i$, yet it is not surrounded by the compact convex sets $\Delta _1,\Delta _2,\Delta _{d+1}$. Hence, there is a hyperplane (i.e., a line) that intersects $\Delta _0$ and at least 2 sets among $\Delta _1,\Delta _2$ and $\Delta _3$. In particular, the family $\{\Delta _0,\Delta _1,\Delta _2,\Delta _3\}$ is not separated. Right: Lemma 2.3 in $\mathbb {R}^2$ – moving a common transversal hyperplane (i.e., a line) *H* to $\Sigma =\{\Delta _1,\Delta _2,\Delta _3\}$ to a position in which it contains a pair of vertices while still intersecting every closed simplex $\Delta _i\in \Sigma$

Let $\Delta _1,\ldots ,\Delta _{d+1}$ be d+1 distinct compact convex sets in $\mathbb {R}^{d}$, and $\Delta _0$ be a compact convex set in $\mathbb {R}^d$ that intersects ${{\texttt{conv}}}\left( \bigcup _{i=1}^{d+1} \Delta _i\right)$, and yet is *not* surrounded by $\Delta _1,\ldots ,\Delta _{d+1}$. Then there exists a hyperplane that intersects $\Delta _0$ together with at least *d* among the remaining sets $\Delta _i$, with $1\le i\le d+1$. Refer to Fig. 4 (left).

#### Lemma 2.3

Let $\Sigma =\{\Delta _1,\ldots ,\Delta _k\}$ be a family of k≥1 closed simplices $\Delta _i$ in $\mathbb {R}^d$ with pairwise disjoint vertex sets $V(\Delta _i)$, and so that the point set $\biguplus _{i=1}^k V(\Delta _i)$ is in general position, and has cardinality at least *d*. Furthermore, suppose that Σ admits a transversal by a hyperplane. Then there is such a hyperplane that passes through some *d* distinct vertices of $\biguplus _{i=1}^k V(\Delta _i)$, and intersects every simplex in Σ. See Fig. 4 (right).

#### Corollary 2.4

Let $\Delta _0,\Delta _1,\ldots ,\Delta _{d+1}$ be d+2 distinct compact convex sets in $\mathbb {R}^{d}$, so that $\Delta _0$ intersects ${{\texttt{conv}}}\left( \bigcup _{i=1}^{d+1} \Delta _i\right)$. Then $\Delta _0$ is surrounded by $\Delta _1,\ldots ,\Delta _{d+1}$ if and only if the family $\{\Delta _0,\Delta _1,\ldots ,\Delta _{d+1}\}$ is separated.

For the sake of brevity, we say that a *point* $q\in \mathbb {R}^d$ is *surrounded* by d+1 sets $\Delta _1,\ldots ,\Delta _{d+1}\subseteq \mathbb {R}^{d}$ if the singleton set $\Delta _0:=\{q\}$ is surrounded by $\Delta _1,\ldots ,\Delta _{d+1}$.

### Simplicial Hypergraphs

Our improved weak ϵ-net construction relies on the intrinsic structure of certain simplicial hypergraphs that are induced by the ground set *P* (and by certain $\lceil \epsilon n\rceil$-size subsets within *P*).

#### Definition

Let $1\le k\le d$. A *k-uniform hypergraph in* $\mathbb {R}^d$ is a pair (*P*, *E*), where *P* is a finite point set in a general position in $\mathbb {R}^d$, and $E\subseteq {P\atopwithdelims ()k}$ is a family of *k*-subsets which can be identified with (k-1)-dimensional simplices whenever $1\le k\le d+1$. In the vast majority of the instances in the sequel, we have k=d, so *E* consists of (d-1)-dimensional simplices.

**Joins.** Given a collection $\Pi \subseteq {P\atopwithdelims ()d-1}$ of (d-2)-dimensional simplices, and a finite point set $X\subseteq \mathbb {R}^d\setminus P$ (in general position with respect to the underlying set *P*), we define their *join* as $\Pi *X:=\{{{\texttt{conv}}}(\tau \cup \{p\})\mid \tau \in \Pi ,p\in X\}$. That is, Π∗X consists of all the (d-1)-dimensional simplices so that d-1 of their vertices form a hyperedge of Π, and the remaining vertex belongs to *X*.

**Fig. 5:**
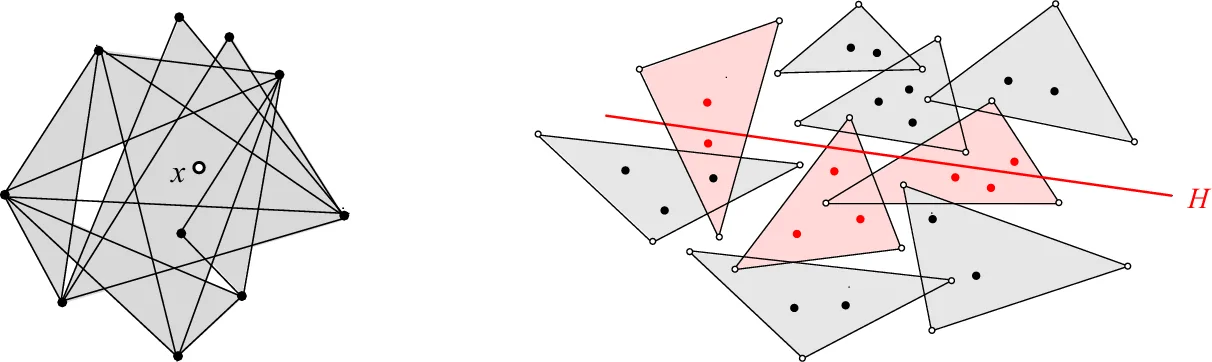
Left: The Second Selection Theorem. Depicted is a 3-uniform simplicial hypergraph (*P*, *E*), in dimension d=2; the edge set *E* consists of at least $h{n\atopwithdelims ()3}$ triangles, and the theorem yields a point $x\in \mathbb {R}^2$ that pierces $\Omega \left( h^{\beta _2}{n\atopwithdelims ()3}\right)$ of these triangles. Right: Matoušek’s Simplicial Partition in dimension d=2 (the zone of the hyperplane *H* is colored red)

**Selection theorems.** At the heart of our improved construction lies the following crucial property of simplicial hypergraphs, due to Alon, Bárány, Füredi, and Kleitman [1]. See [43, Sect. 9] and [54] for a comprehensive exposition, and Fig. 5 (left) for an illustration in dimension 2.

#### Theorem 2.5

(“The Second Selection Theorem”) For every dimension d≥1 there exists a constant $\beta _d>0$ with the following property:

Let (*P*, *E*) be a (d+1)-uniform simplicial hypergraph in $\mathbb {R}^d$ with $|E|=h{n\atopwithdelims ()d+1}$ edges, then there is a point $x\in \mathbb {R}^d$ that pierces at least $\Omega \left( h^{\beta _d}{n\atopwithdelims ()d+1}\right)$ of the *d*-simplices of *E*.

While the original proof of Alon et al. yields $\beta _d\le (4d+1)^{(d+1)}$, the best known estimate for the second selection theorem (due to the author [54]) yields the exponent $\beta _d\le d^5+O\left( d^4\right)$, and even better ad-hoc bounds for $\beta _d$ are known in dimension 2; see, e.g., [31, 50].^5^

### Matoušek’s Simplicial Partition Theorem

Let *P* be a set of *n* points in $\mathbb {R}^d$, and r>0 be an integer. A *simplicial* *r-partition* $\mathcal {P}$ of *P* is a collection $\{(P_i,\Delta _i)\mid 1\le i\le r\}$ of *r* pairs, where for each $1\le i\le r$ we have that $P_i\subset P$ and $\Delta _i$ is a simplex of dimension at most *d* in $\mathbb {R}^d$, so that the following properties are satisfied^6^:

1. $$P=\biguplus _{i=1}^r P_i$$.
2. For each $1\le i\le r$ so that $P_i\ne \emptyset$, the cardinality $n_i:=|P_i|$ of $P_i$ satisfies $\lceil n/r\rceil \le n_i<2\lceil n/r\rceil .$
3. For each $1\le i\le r$, the set $P_i$ is contained in the relative interior of $\Delta _i$.

#### Definition

In the sequel, we refer to the simplices $\Delta _i$, that enclose the parts $P_i$ in $\mathcal {P}$, as the *cells* of the partition $\mathcal {P}$.^7^ For each set $P_i$ in an *r*-partition $\mathcal {P}=\{(P_i,\Delta _i)\mid 1\le i\le r\}$ of *P*, and each point $p\in P_i$, we refer to $\Delta _i$ as the *ambient cell* of *p* in $\mathcal {P}$.

For any hyperplane *H* in $\mathbb {R}^d$, we say that a point $x\in \mathbb {R}^d$ *lies in the zone of* *H* *within* $\mathcal {P}$ if its ambient cell is intersected by *H*.

#### Theorem 2.6

(The Simplicial Partition Theorem) For any d≥2 there is a constant $c_{ sp}=c_{ sp}(d)$ with the following property. For any *n*-point set, and any $1\le r\le n$, there is a simplicial *r*-partition $\{(P_i,\Delta _i)\mid 1\le i\le r\}$ so that any hyperplane crosses at most $c_{ sp}\cdot r^{1-1/d}$ cells $\Delta _i$, for $1\le i\le r$. See Fig. 5 (right). Furthermore, such a partition $\mathcal {P}$ can be computed in time $O^*(n)$.^8^

Thus, the zone of any hyperplane within the partition of Theorem 2.6 encompasses a total of $O\left( n/r^{1/d}\right)$ points of *P*.

For any d≥2, any *n*-point set $P\subset \mathbb {R}^{d}$, and any $1\le r\le n$, we fix a unique partition $\mathcal {P}=\mathcal {P}_d(P,r)$ as in Theorem 2.6, which we simply denote by $\mathcal {P}(P,r)$ when the ambient dimension of *P* is clear from the context.

If the points of the underlying set *P* are in a general position, it can be assumed that all the cells $\Delta _i$ in Theorem 2.6 are *d*-dimensional; furthermore, their vertices can be perturbed in a general position with respect to one another, and with respect to the point set *P*. As a result, it can be assumed that no hyperplane *intersects* more than $c_{ sp}\cdot r^{1-1/d}$ of the cells $\Delta _i$.

### Arrangements of Hyperplanes in $\mathbb {R}^d$

**Supporting hyperplanes.** Any *d*-point set (i.e., a (d-1)-dimensional simplex) *S* in a general position in $\mathbb {R}^d$ determines a unique hyperplane $H(S)={{\texttt{aff}}}(S)$. We then say that *H*(*S*) is *spanned* by *S*, while the point set (or the simplex) *S* is *supported* by *H*(*S*).

Given a subset $\Pi \subset {A\atopwithdelims ()d}$ of (d-1)-dimensional simplices that are determined by a point set *A* in general position, we use ${\mathcal {H}}(\Pi )$ to denote the set $\{H(S)\mid S\in \Pi \}$ of all the hyperplanes that support the elements of Π.

**Hyperplane arrangements.** Any finite family ${\mathcal {H}}$ of *m* hyperplanes in $\mathbb {R}^d$ induces the *arrangement* $\mathcal {A}({\mathcal {H}})$ – the partition of $\mathbb {R}^d$ into open faces. The *d*-dimensional faces of $\mathcal {A}({\mathcal {H}})$ are called *cells*, and they are maximal connected regions of $\mathbb {R}^d\setminus \left( \bigcup {\mathcal {H}}\right)$. Each cell is a convex polyhedron whose boundary is comprised of *k*-dimensional faces of $\mathcal {A}({\mathcal {H}})$, for $0\le k\le d-1$; each of these faces is a *k*-dimensional polyhedron which lies within a *k*-dimensional plane – an intersection of some d-k of the hyperplanes of ${\mathcal {H}}$. Specifically, the (d-1)-dimensional faces of $\mathcal {A}({\mathcal {H}})$ are (d-1)-dimensional polyhedral portions of the hyperplanes of ${\mathcal {H}}$. The 0-dimensional faces are called *vertices*, and each of them is an intersection of some *d* hyperplanes in ${\mathcal {H}}$.

It is known that an arrangement $\mathcal {A}({\mathcal {H}})$ of *n* hyperplanes encompasses a total of $O\left( n^d\right)$ faces of all dimensions (and this bound is tight for ${\mathcal {H}}$ in a general position).

The *complexity* $\mathsf{{\varpi }}(\Delta )$ of a polyhedral cell Δ of $\mathcal {A}({\mathcal {H}})$ is the total number of faces, whose dimensions may vary from 0 and d-1, that lie on its boundary. See, e.g., [43, Sect. 6] for a more comprehensive background on hyperplane arrangements.

One can restrict the unbounded faces in $\mathcal {A}({\mathcal {H}})$ by augmenting ${\mathcal {H}}$ with a pair of imaginary hyperplanes that are orthogonal to the *i*-th axis, $H^+_{i}:=\{X_i=\infty \},H^-_i:=\{X_i=-\infty \}$, for each $1\le i\le d$. With this adjustment, every original cell in $\mathcal {A}({\mathcal {H}})$ now corresponds to exactly one bounded *polytopal* cell in the augmented arrangement $\mathcal {A}({\mathcal {H}})$. In what follows, we can restrict our analysis to these latter cells.

#### Theorem 2.7

Let ${\mathcal {H}}$ be a family of *m* hyperplanes in $\mathbb {R}^d$, and 0<r≤m integer. Then, with probability at least 1/2, an *r*-sample $\mathcal {R}\in {{\mathcal {H}}\atopwithdelims ()r}$ of ${\mathcal {H}}$ crosses every open segment in $\mathbb {R}^d$ that is intersected by at least c(m/r)logr hyperplanes of ${\mathcal {H}}$.^9^ Here c>0 is a sufficiently large constant depending only on *d*, but not on *m* or *r*.

The proof of Theorem 2.7 can be found, e.g., in [23]. It is established by applying the Epsilon Net Theorem [36] to the range space of hypergraphs in which every vertex set is a finite family ${\mathcal {H}}$ of hyperplanes in $\mathbb {R}^d$, and every edge consists of all the hyperplanes in ${\mathcal {H}}$ that are crossed by a certain segment with $\mathbb {R}^d$.

**The bottom-vertex triangulation.** Every polytopal cell Δ of $\mathcal {A}({\mathcal {H}})$ can be decomposed into a collection $\mathcal {D}_\Delta$ of interior-disjoint *d*-dimensional simplices that share the lowermost boundary vertex *v* of Δ.^10^ Each of these simplices is of the form $\tau ={{\texttt{conv}}}\left( \{v\}\cup \kappa \right)$ where κ is a (d-1)-dimensional simplex that is not adjacent to *v*, and arises in a recursive triangulation of some (d-1)-dimensional face on the boundary of Δ. Let $\mathcal {D}({\mathcal {H}})$ denote the resulting collection of interior-disjoint simplices $\bigcup _{\Delta \in \mathcal {A}({\mathcal {H}})}\mathcal {D}_\Delta$ that comprise the bottom-vertex triangulations of all the *d*-cells of $\mathcal {A}({\mathcal {H}})$. (A more comprehensive exposition of this fundamental structure can be found, e.g., in [21, 25].)

An easy consequence of Theorem 2.7 is that, with probability at least 1/2, every cell of the bottom-vertex triangulation $\mathcal {D}(\mathcal {R})$, that is determined by such a sample $\mathcal {R}$, is crossed by at most (d+1)C(m/r)logr hyperplanes of ${\mathcal {H}}$; in other words, it serves as an $\left( \frac{(d+1)C\log r}{r}\right)$*-cutting of* ${\mathcal {H}}$ [43, Sect. 4.6]. (This is because every hyperplane of ${\mathcal {H}}$ that crosses a simplex τ of $\mathcal {D}(\mathcal {R})$ must also cross at least *d* of the d+12 1-dimensional faces, or edges, of τ.)

**Zones.** Let $\mathcal {C}$ be a family of polytopal cells in $\mathbb {R}^d$ (e.g., cells in the above arrangement $\mathcal {A}({\mathcal {H}})$, the *d*-dimensional simplices of its refinement $\mathcal {D}({\mathcal {H}})$, or the cells in Matoušek’s partitioning theorem). The *zone* of any set $X\subset \mathbb {R}^d$ in $\mathcal {C}$ is the subset of all the cells in $\mathcal {C}$ that intersect *X*.

The following elementary property can be instantly deduced through induction on the ambient dimension *d*, and it is explicitly established by Aronov, Pellegrini, and Sharir [10, Lemmas 2.2 and 2.3] as the basis for a more general bound; also see [55].

#### Lemma 2.8

Let ${\mathcal {H}}$ be a collection of *n* hyperplanes in $\mathbb {R}^d$, and $K\subset \mathbb {R}^d$ a convex set. Then the boundary ∂K of *K* intersects $O\left( n^{d-1}\right)$ cells in the arrangement $\mathcal {A}({\mathcal {H}})$.

**The complexity of many cells.** Note that for any collection ${\mathcal {H}}$ of *n* hyperplanes, the overall complexity of all the cells in their arrangement $\mathcal {A}({\mathcal {H}})$ is $O\left( n^d\right)$. (This is because, after perturbing ${\mathcal {H}}$ into a general position, every *k*-dimensional face is adjacent to at most $2^{d-k}$ cells.) However, if $\mathcal {C}$ is a subset of cells in $\mathcal {A}({\mathcal {H}})$ whose cardinality *m* is much smaller than $n^d$, then the overall complexity $\mathsf{{\varpi }}(\mathcal {C})=\sum _{\Delta \in \mathcal {C}}\mathsf{{\varpi }}(\Delta )$ is $o\left( n^d\right)$.

#### Theorem 2.9

(Aronov, Matousek, Sharir 1994 [8]; Aronov, Sharir 2004 [9]) Let ${\mathcal {H}}$ be a collection of *n* hyperplanes in $\mathbb {R}^d$. Then for any collection $\mathcal {C}$ of *m* cells in $\mathcal {A}({\mathcal {H}})$, their overall complexity $\mathsf{{\varpi }}(\mathcal {C})$ is $O\left( m^{1/2}n^{d/2}\log ^{\zeta _d}n\right) ,$ where $\zeta _d=\left( \lfloor d/2\rfloor -1\right) /2$.

### A Recursive Framework for Bounding $f_d(\epsilon )$

Let us now lay down a more formal framework for the recursive analysis of the weak ϵ-numbers $f_d(\epsilon )$, in which the proof of Theorem 1.1 will be cast.

#### Definition

For a finite point set *P* in $\mathbb {R}^d$ and ϵ>0, let ${{\mathcal {K}}}(P,\epsilon )$ denote the family of all the ϵ-heavy convex sets with respect to *P*. Theorem 1.1 will be established in a divide-and-conquer manner, by subdividing the elements of ${{\mathcal {K}}}(P,\epsilon )$ into several finer sub-classes ${{\mathcal {K}}}$, and then constructing a separate net for each class ${{\mathcal {K}}}$. For the sake of brevity, we say that $N\subset \mathbb {R}^d$ is *a weak* ϵ*-net* for a family ${{\mathcal {K}}}$ of convex sets in $\mathbb {R}^d$ if it pierces every in ${{\mathcal {K}}}$ that is ϵ-heavy with respect to the *P*. If the parameter ϵ>0 is fixed, we simply say that *N* pierces ${{\mathcal {K}}}$ (thereby assuming, with no loss of generality, that all sets in ${{\mathcal {K}}}$ are ϵ-heavy).

**Recurrence in** ϵ **and** *P*. To simplify the exposition, every set $K\in {{\mathcal {K}}}(P,\epsilon )$ will be assigned a unique *principal subset* $P_K\subseteq P\cap K$ of exactly $\lceil \epsilon n\rceil$ points. Furthermore, for the sake of our inductive analysis, every finite set *P* in $\mathbb {R}^d$, and every $0<\epsilon \le 1$, will be assigned a unique weak ϵ-net N(P,ϵ) whose cardinality is at most $f_d(\epsilon )$.

To bound the quantity $$f_d(\epsilon )$$, the previous recursive arguments (e.g., by Chazelle et al. [24] for points in convex position in $$\mathbb {R}^2$$, and Matoušek and Wagner [45] for the general case) proceed by fixing the ground set $$P\subset \mathbb {R}^d$$, and constructing a net *N* for the induced family $${{\mathcal {K}}}(P,\epsilon )$$. An upper bound on |*N*| then serves as an upper bound on $$f_d(\epsilon )$$. This goal is achieved in a top-down fashion, by tackling a number of more “basic” instances $${{\mathcal {K}}}(P',\epsilon ')$$ which typically involve *smaller* ground sets $P^{\prime }\subset P$ yet *larger* parameters $$\epsilon '>\epsilon $$.

**Towards a sharper recurrence: constraining the simplices of** ${P_K\atopwithdelims ()d}$. At the center of our improved construction lies the existence of $o\left( 1/\epsilon ^d\right)$-size nets for certain “simplex-constrained” sub-families of ϵ-heavy convex sets. Each of these finer sub-families within ${{\mathcal {K}}}(P,\epsilon )$ is further constricted by a certain subset Π of $o\left( n^d\right)$ simplices within Pd, and consists of the convex sets $K\in {{\mathcal {K}}}(P,\epsilon )$ whose induced subcollections ${P_K\atopwithdelims ()d}$ of (d-1)-simplices are either contained in, or have a considerable overlap with Π.

Thus, to further amplify the resulting $o(1/\epsilon ^d)$ estimate on $f_d(\epsilon )$, a basic sub-family of convex sets *K* in our recurrence will be characterized not only by the ground set *P* and the ratio ϵ>0, but also by a certain collection $\Pi \subseteq {P\atopwithdelims ()d}$, which must encompass almost ${\lceil \epsilon n\rceil \atopwithdelims ()d}$ of the (d-1)-simplices that are spanned by the principal subset $P_K$. As a result, our recurrence will advance not only by increasing the ratio ϵ>0, but also by restricting our convex sets to “include” many simplices from a progressively *sparser* sub-family Π within Pd.

**The families** ${{\mathcal {K}}}(P,\Pi ,\epsilon ,\sigma )$. Let $0<\sigma \le 1$ and $0<\epsilon \le 1$, let *P* be an *n*-point set in $\mathbb {R}^d$, and let Π be a subset of (d-1)-simplices within Pd. We say that a convex set $K\in {{\mathcal {K}}}(P,\epsilon )$ is $(\epsilon ,\sigma )$*-restricted* to the *d*-uniform hypergraph (P,Π) if its induced subset of (d-1)-simplices ${P_K\atopwithdelims ()d}$ contains at least $\sigma {\lceil \epsilon n\rceil \atopwithdelims ()d}$ elements of Π.

In each recursive step, we seek to construct a weak ϵ-net *N* that pierces a certain family ${{\mathcal {K}}}={{\mathcal {K}}}(P,\Pi ,\epsilon ,\sigma )$, which is determined by ϵ>0, a finite point set $P\subset \mathbb {R}^d$, a set of hyperedges $\Pi \subseteq {P\atopwithdelims ()d}$, and a threshold $0<\sigma \le 1$, and is comprised of all the ϵ-heavy convex sets $K\in {{\mathcal {K}}}(P,\epsilon )$ that are $(\epsilon ,\sigma )$-restricted to the hypergraph (P,Π).

In what follows, we refer to the hypergraph (P,Π) (or, simply, to Π, once the ground set *P* is clear from the context) as the *restriction hypergraph*, and to σ as the *restriction threshold* of the family ${{\mathcal {K}}}(P,\Pi ,\epsilon ,\sigma )$.

All the recursive instances in the sequel will involve positive thresholds σ that are either constant, or bounded from below by an arbitrary small (albeit, positive) power of ϵ.^11^

**The improved recurrence for** $f_d(\epsilon )$. For any choice of $0<\epsilon ,\sigma \le 1$ and $0<\rho \le 1$, we use $f(\epsilon ,\rho ,\sigma )$ to denote the smallest number *f* so that for any finite point set *P* in $\mathbb {R}^d$, and any subset $\Pi \subseteq {P\atopwithdelims ()d}$ of density $|\Pi |/{n\atopwithdelims ()d}\le \rho$, there is point transversal of size at most *f* to ${{\mathcal {K}}}(P,\Pi ,\epsilon ,\sigma )$. In addition, we set $f(\epsilon ,\rho ,\sigma )=1$ whenever ϵ=1.

Since the underlying dimension *d* is fixed, for the sake of brevity we use f(ϵ) to denote the quantity $f_d(\epsilon )=f(\epsilon ,1,1)$, and note that the trivial bound $f(\epsilon ,\rho ,\sigma )\le f(\epsilon )$ always applies.

To establish Theorem 1.1, in Sect. 4 we first derive a recurrence for the quantity $f(\epsilon ,\rho ,\sigma )$, which takes the following more general form:

2 $$\begin{aligned} f(\epsilon ,\rho ,\sigma )\le f\left( \epsilon ,\rho /h,\sigma /2\right) +\sum _{i=1}^l r_i \cdot f\left( \epsilon \cdot r_i^{1/a_i}\right) +O\left( 1/\epsilon ^{\alpha _d+\eta }\right) . \end{aligned}$$

Here η>0 is an arbitrary small constant which is far smaller than our target constant γ>0 in Theorem 1.1, $l=O(\log (1/\epsilon ))$, $\alpha _d$ is the exponent in Theorem 1.1, $a_i\le \alpha _d+\eta$ for all $1\le i\le l$, while *h* and all the remaining parameters $r_i$ are various positive powers of 1/ϵ that are far smaller than $1/\epsilon ^{\alpha _d}$.

To bound the quantity f(ϵ), we will invoke the general recurrence (2) with $\rho =\sigma =1$. This double recurrence in ϵ and ρ will bottom out when either (i) the maximum density ρ of Π falls below ϵ, or (ii) ϵ bypasses a certain constant threshold $\epsilon _0$ that is determined in the sequel. In the former case, our 1-dimensional reduction in Sect. 3 will yield an “$O\left( 1/\epsilon ^{d-1}\right)$-type” bound of the form $f(\epsilon ,\rho ,\sigma )=O^*\left( r\cdot f\left( \epsilon \cdot r^{1/(d-1)} \right) +\frac{1}{\epsilon ^{d-1}}\right)$. In the latter case, we can use the $\tilde{O}\left( \left( 1/\epsilon _0\right) ^{d}\right) =O(1)$ bound of Chazelle et al. [24]. By keeping the main parameter ϵ>0 fixed, and following through $\log _h(1/\epsilon )=O(1)$ applications of (2) to its first term, one can get rid of the term $f(\epsilon ,\rho ,\sigma )$. The remaining recurrence (in ϵ alone) will then solve to $\displaystyle f(\epsilon )=O\left( (1/\epsilon )^{\alpha _d+\gamma }\right)$ using the standard substitution methodology, as described in [45, 57] and [58, Section 7.3.2].

### A Roadmap to the Proof of Theorem 1.1

Here is a more comprehensive outline of our improved weak ϵ-net construction, and its subsequent analysis. Recall that every convex set *K* that is ϵ-heavy with respect to the underlying point set *P*, has been assigned a unique *principal subset* $P_K\subseteq P\cap K$ of $\lceil \epsilon n\rceil$ points, which determine a collection of ${|P_K|\atopwithdelims ()d}=\Theta \left( \epsilon ^dn^d\right)$ of (d-1)-dimensional simplices.

As was mentioned in the Introduction, these simplex families $${P_K\atopwithdelims ()d}$$ are quintessential to our reduction of the weak ϵ-net problem, in any dimension d≥3, to a small number of 1-dimensional strong $$\Theta ^*\left( \epsilon ^{\alpha _d}\right) $$-nets *with respect intervals of* $$\mathbb {R}^1$$. Notice that the reduction is incomplete and relegates certain “leftover” convex sets $$K\in {{\mathcal {K}}}(P,\epsilon )$$ to recursive instances $${{\mathcal {K}}}(P',\epsilon ')$$ which entail larger density values $\epsilon^{\prime }>0$ and smaller-size subsets $P^{\prime }$ of *P*.

#### A Warm-Up in $\mathbb {R}^2$

**Fig. 6:**
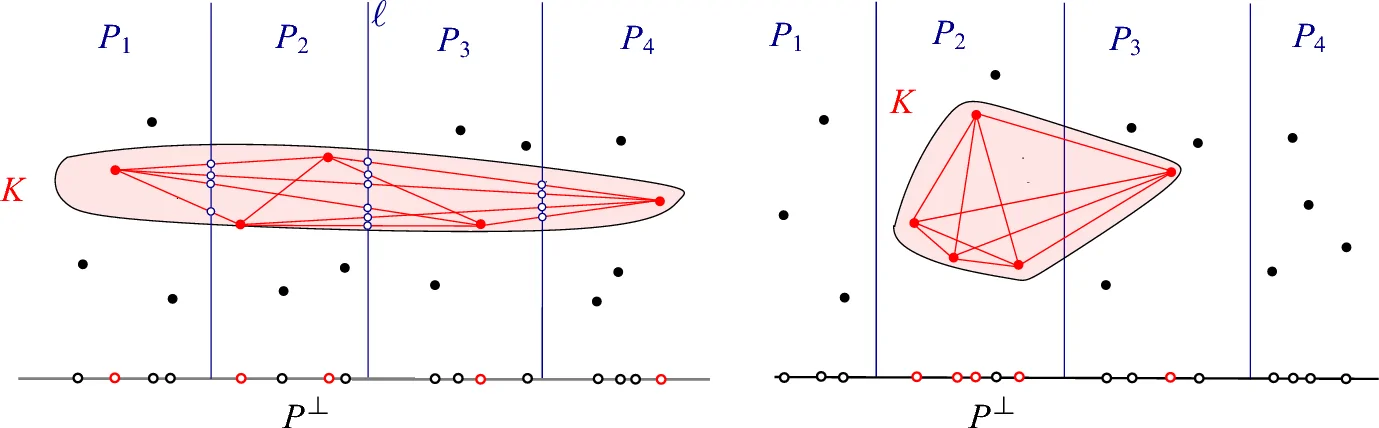
Finding a small canonical line set $\mathcal {{L}}$ of *s* lines in $\mathbb {R}^2$ (with s=3). The set *P* is divided by $\mathcal {{L}}$ into s+1 parts $P_1,\ldots ,P_{s+1}$ of cardinality at most $\lceil n/(s+1)\rceil$ each. The induced partition of the projected set $P^\perp$ in $\mathbb {R}^1$ is also depicted. Left: If no part $P_i$ contains more than $|P_i|/4$ of the points of $P_K$ (which are marked as red), then the majority of the edges in ${P_K\atopwithdelims ()2}$ must cross one or more lines of $\mathcal {{L}}$. Right: If some part $P_i$ contains a significant fraction of $P_K$, then we have that $|P_i\cap K|/|P_i|\gg \epsilon s$

Let us first illustrate this phenomenon in $\mathbb {R}^2$. To establish the bound $f_2(\epsilon )=O^*\left( 1/\epsilon ^{3/2}\right)$ [53], the author fixes a small integer parameter s>0, and uses a family $\mathcal {{L}}$ of *s* vertical lines to subdivide the plane into s+1 slabs $\Delta _1,\ldots ,\Delta _{s+1}$, each inducing a subset $P_i=P\cap \Delta _i$ of at most $\lceil n/(s+1)\rceil$ points of *P*.^12^ See Fig. 6.

Let $K\in {{\mathcal {K}}}(P,\epsilon )$. If no slab $\Delta _i$ contains more than, say, $|P_K|/4$ of the points of $P_K$ (which happens if the projected points of $P^\perp _K$ are reasonably “well distributed” in the linear ordering of $P^\perp$, as depicted in Fig. 6 (left)), then the vast majority of the edges in ${P_K\atopwithdelims ()2}$ must cross one or more lines $\ell \in \mathcal {{L}}$ – each time within the respective cross-section $K\cap \ell$. In particular, there must exist such a line $\ell \in \mathcal {{L}}$ crossing $\Omega \left( \epsilon ^2n^2/s\right)$ of the edges in ${P_K\atopwithdelims ()2}$. Thus, convex sets *K* of this sort can be pierced by constructing, for each $\ell \in \mathcal {{L}}$, a strong $\Theta \left( \epsilon ^2/s\right)$-net with respect to the 1*-dimensional* ground set $X_\ell \subset \ell$ which is comprised of $O(n^2)$ (non-empty) ℓ-intercepts $pq\cap \ell$ of the edges $pq\in {P\atopwithdelims ()2}$.

On the other hand, if a sizable fraction of the $\lceil \epsilon n \rceil$ points in $P_K$ fall into a single slab $\Delta _i$ of $\mathbb {R}^2\setminus \left( \bigcup \mathcal {{L}}\right)$ (as depicted in Fig. 6 (right)), then *K* belongs to the family ${{\mathcal {K}}}(P_i,\epsilon ')$, with $\epsilon '\gg \epsilon n/|P_i|=\Theta (\epsilon \cdot s)$. As a result, sets *K* of this kind can be dispatched by constructing a weak $\Theta (\epsilon \cdot s)$-net for each part $P_i$, which adds only a “linear” term $O\left( s\cdot f_2(\epsilon \cdot s)\right)$ to the overall “quadratic” recurrence $f_2(\epsilon )=O\left( s\cdot f_2(\epsilon \cdot s)+s^2/\epsilon ^2\right)$ in ϵ.^13^

By specializing to a certain class of “line-like” sets *K*, the family P2 can be effectively replaced with a sparser subset of $O^*\left( \sqrt{\epsilon }\cdot n^2\right)$ edges, which allows for considerably smaller ground sets $Y_\ell \subseteq X_\ell$. As a result, the cardinality of our 1-dimensional nets can be reduced to $O^*\left( 1/\epsilon ^{3/2}\right)$. (The remaining, substantially “fat” sets are dispatched by an auxiliary net whose cardinality is too bounded by $O^*\left( 1/\epsilon ^{3/2}\right)$.)

#### Dealing with Higher-Dimensional Point Sets

**Fig. 7:**
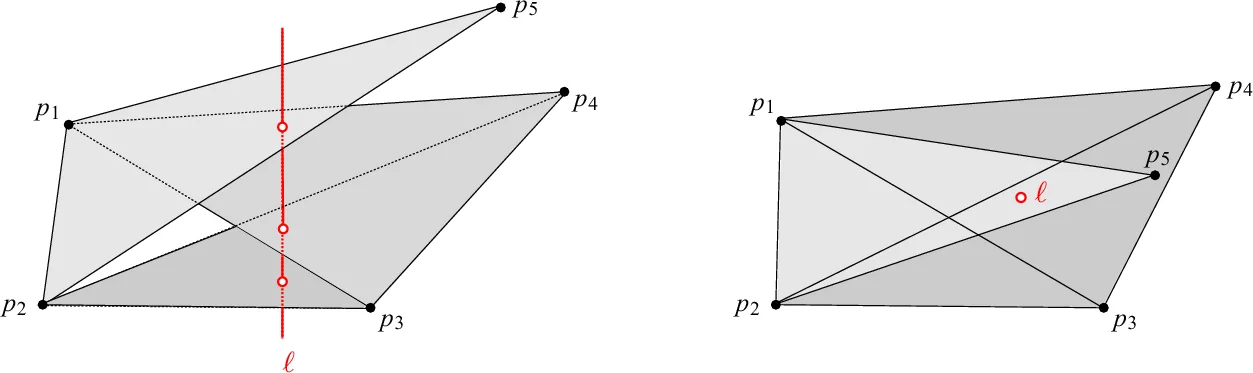
The reduction to 1-dimensional nets illustrated in $\mathbb {R}^3$. The vertical line ℓ pierces the triangles $\triangle p_1p_2p_5, \triangle p_3p_4p_1,\triangle p_3p_4p_2$, which are determined by $P_K=\{p_1,\ldots ,p_5\}$ (left), if and only if the projection of ℓ pierces the projections of the 3 triangles (right). Note that the ℓ-intercepts of the triangles lie within $\ell \cap {{\texttt{conv}}}(p_1,\ldots ,p_5)\subseteq K\cap \ell$

To adapt the outlined strategy to dimensions d≥3, let us fix an ϵ-heavy convex set $K\in {{\mathcal {K}}}(P,\epsilon )$. Let ℓ be any vertical line that pierces $\xi {|P_K|\atopwithdelims ()d}$ of the simplices of ${P_K\atopwithdelims ()d}$, for some constant ξ that depends only on the dimension *d*. Then, by the convexity of *K*, the interval $K\cap \ell$ must contain $\xi {|P_K|\atopwithdelims ()d}$ of the ℓ-intercepts ${{\texttt{conv}}}(\tau )\cap \ell$ of the simplices $\tau \in {P_K\atopwithdelims ()d}$. See Fig. 7 (left). Hence, all such sets *K* can be pierced by including in our net $N=N_\ell$ every $\lceil C\epsilon ^dn^d\rceil$-th point of the set $X_\ell =\left\{ {{\texttt{conv}}}(\tau )\cap \ell \mid \tau \in {P\atopwithdelims ()d}\right\}$, whose cardinality is at most ${n\atopwithdelims ()d}=O\left( n^d\right)$; here C>0 is a constant that depends on ξ. In other words, we construct *a strong* ν*-net* with respect to the *1-dimensional set* $X_\ell \subseteq \ell$, and with

3 $$\begin{aligned} \nu =\Omega \left( \frac{{|P_K|\atopwithdelims ()d}}{|X_\ell |}\right) =\Omega \left( \frac{{\epsilon n\atopwithdelims ()d}}{{n\atopwithdelims ()d}}\right) =\Omega \left( \epsilon ^d\right) . \end{aligned}$$

By using a *small* family $\mathcal {{L}}$ of canonical vertical lines ℓ, and effectively replacing the set Pd in the definition of $X_\ell$ with its more elaborately defined subset of $O^*(\epsilon ^{d-\alpha _d}n^d)$ simplices (which would still suit the above 1-dimensional reduction), our divide-and-conquer strategy will increase the density parameter ν from $\epsilon ^d$ to roughly $\epsilon ^{\alpha _d}$, and ultimately yield a system of ν-nets $N_\ell \subset \ell$ of overall cardinality $O^*(1/\nu )=O^*\left( 1/\epsilon ^{\alpha _d}\right)$.

**Crossing** (d-1)**-simplices with vertical lines.** For a fixed $K\in {{\mathcal {K}}}(P,\epsilon )$, the existence of *at least one* such vertical line ℓ that would pierce many of the (d-1)-simplices in ${P_K\atopwithdelims ()d}$, is guaranteed by the point-selection theorem of Alon et al. [1] (i.e., Theorem 2.5). To see this, it is enough to consider the vertical projection $P_K^\perp$ of $P_K$ (see Fig. 7 (right)), apply the lemma to the complete *d*-uniform hypergraph ${P_K^\perp \atopwithdelims ()d}$ (with h=1), and lift the resulting point *x* back to a vertical line $x^*$ which now pierces a large fraction of the simplices of ${P_K\atopwithdelims ()d}$.^14^

However, finding a *complete* canonical set $\mathcal {{L}}$ of vertical lines, so that at least one of them would suit *every* ϵ-heavy convex set *K*, is at least as hard as finding a weak ϵ-net with respect to the (d-1)-dimensional projection of *P*. (In other words, such a canonical family $\mathcal {{L}}$ may require at least $f_{d-1}(\epsilon )$ vertical lines, for some ground sets *P*.)

##### Remark

To get around this problem, Chazelle et al. [24] cross the simplices of Pd using a fairly large canonical family $\mathcal {{L}}$ of vertical lines. Each line $\ell \in \mathcal {{L}}$ is assigned a carefully selected subset $\Pi _\ell \subseteq {P\atopwithdelims ()d}$ of simplices (all of them crossed by ℓ); its ν-net $N_\ell$ is then constructed with respect to the 1-dimensional set $X_\ell :=\{{{\texttt{conv}}}(\tau )\cap \ell \mid \tau \in \Pi _\ell \}$, and with $\nu =\tilde{\Omega }\left( \epsilon ^dn^d/|\Pi _\ell |\right)$ that depends on $|\Pi _\ell |$. Despite the considerable size of the canonical set $\mathcal {{L}}$, the ultimate cardinality of the net $\bigcup _{\ell \in \mathcal {{L}}}N_\ell$ is shown to be only $\tilde{O}\left( 1/\epsilon ^d\right)$. While this method readily yields the bound $f_d(\epsilon )=\tilde{O}\left( 1/\epsilon ^d\right)$, it is of little use to our more elaborate scheme, which calls for a *small* canonical line set $\mathcal {{L}}$.

#### Finding a Small Canonical Set of Vertical Lines

As is shown in Sect. 3, the desired *small-size* canonical set $\mathcal {{L}}$ of vertical lines can be attained given that, for each convex set at hand, the vertical projection $P^\perp _K$ of its principal set $P_K$ is reasonably “well-spread” within the projection $P^\perp \subseteq \mathbb {R}^{d-1}$ of the entire ground set *P*. (This condition is met, e.g., if the points of $P^\perp _K$ are chosen uniformly and at random from $P^\perp$.)

To this end, the distribution of $P^\perp _K$ within $P^\perp$ can be quantified by again fixing a small integer parameter s>0, and using Theorem 2.6 to obtain the simplicial *s*-partition $\mathcal {P}_{d-1}\left( P^\perp ,s\right)$ of the projected set $P^\perp$ within $\mathbb {R}^{d-1}$. Lifting the sets of $\mathcal {P}_{d-1}(P^\perp ,s)$ back to $\mathbb {R}^d$ yields a *d*-dimensional partition $\mathcal {V}(P,s)=\{\left( P_i,\Delta _i\right) \mid 1\le i\le s\}$ of *P* so that each subset $P_i$ is contained in the vertical prism $\Delta _i^*$ above the respective simplicial cell $\Delta _i\subset \mathbb {R}^{d-1}$, and satisfies $|P_i|\le 2\lceil n/s \rceil$. In the sequel, we simply refer to $\mathcal {V}(P,s)$ as the *vertical* *s-partition* (or, briefly, as the *vertical partition*).

**Fig. 8:**
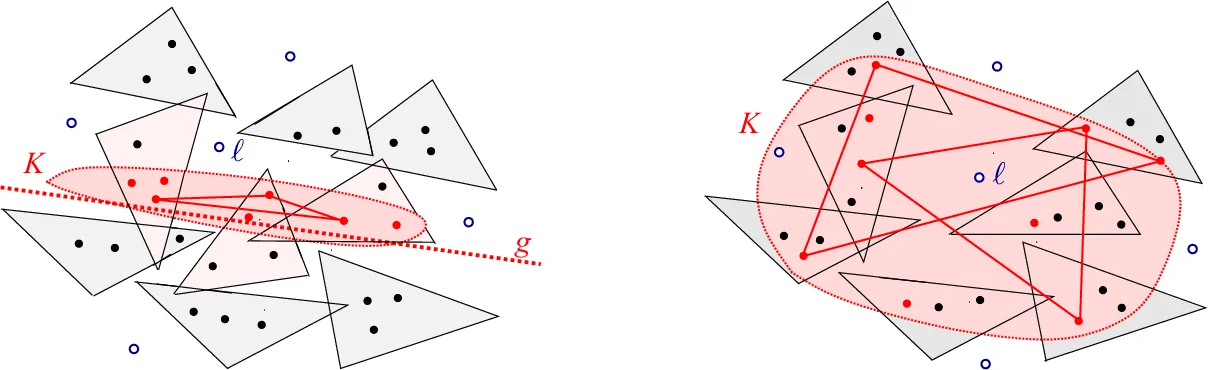
Using the vertical partition $\mathcal {V}(P,s)$ in $\mathbb {R}^3$ – view from above. Left: The set *K* (red) is *narrow*, so the points of $P_K$ project to the zone of the line $g\subseteq \mathbb {R}^2$ (purple), in which case the triangles of ${P_K\atopwithdelims ()3}$ are likely to be missed by our canonical set of vertical lines $\ell \in \mathcal {{L}}$. Right: The set *K* is *spread*, so many triangles in ${P_K\atopwithdelims ()3}$ can be crossed by a line $\ell \in \mathcal {{L}}$

We say that an ϵ-heavy convex set *K* is *narrow* if a significant fraction of the points of $P_K$ fall into only $O\left( s^{1-1/(d-1)}\right)$ parts of $\mathcal {V}(P,s)$, whose ambient cells can be crossed by a single (d-2)-flat *g* in $\mathbb {R}^{d-1}$; see Fig. 8 (left). Otherwise, we say that *K* is *spread* in $\mathcal {V}(P,s)$; see Fig. 8 (right).^15^ Though the simplex families ${P_K\atopwithdelims ()d}$, that are determined by narrow sets *K*, may prove hard for interception by few vertical lines, such sets *K* can be pierced by an auxiliary net whose cardinality is bounded in Lemma 3.1 by an essentially “$O^*\left( 1/\epsilon ^{d-1}\right)$-type” recursive term $O\left( s\cdot f_d\left( \epsilon \cdot s^{1/(d-1)}\right) \right)$.^16^

Specializing to the remaining convex sets *K*, whose principal subsets $P_K$ are more evenly spread among the parts of our vertical partition $\mathcal {V}(P,s)$, our principal line-selection result – Theorem 3.2 – will yield the desired canonical collection $\mathcal {{L}}=\mathcal {{L}}(P,s)$ of at most $s^{(d-1)^2}$ vertical lines, and with the following property: *For each “well-spread”* ϵ*-heavy convex set* *K* *as above, there exists a line* $\ell \in \mathcal {{L}}$ *that pierces a fixed fraction of the simplices of* ${P_K\atopwithdelims ()d}$. See Fig. 8 (right).

Hence, with the aforementioned exception of the narrow sets, all the ϵ-heavy sets *K* can be pierced by a combination of at most $s^{(d-1)^2}$ 1-dimensional strong ν-nets $N_\ell \subset \ell$, for $\ell \in \mathcal {{L}}$ and $\nu =\Omega \left( \epsilon ^d\right)$. Most of our analysis is, therefore, devoted to bringing the effective density ratios ν in the previously described 1-dimensional instances above $\epsilon ^{\alpha _d}$.

#### Attaining $\nu =\Omega ^*\left( \epsilon ^{\alpha _d}\right)$

Informally, to reduce the enormous denominator nd in (3), we seek to replace the complete collection Pd of (d-1)-simplices with some *smaller* subset $\tilde{\Pi }\subset {P\atopwithdelims ()d}$. However, in order for our $\Theta ^*\left( {\epsilon n\atopwithdelims ()d}/|\Pi |\right)$-net to still pierce a given ϵ-heavy convex set *K*, it is essential for such a smaller-size substitute $\tilde{\Pi }$ of Pd to include a large enough fraction of the (d-1)-simplices of ${P_K\atopwithdelims ()d}$. Unfortunately, no single and *small-size* replacement $\tilde{\Pi }$ of Pd can simultaneously suit *all* the ϵ-heavy convex sets $K\in {{\mathcal {K}}}(P,\epsilon )$.

**Flat convex sets are easy to pierce.** It is well known that hypergraphs induced by hyperplane ranges have Vapnik-Chervonenkis dimension *d* and, therefore, admit strong ϵ-nets of cardinality $O\left( \frac{1}{\epsilon } \log \frac{1}{\epsilon }\right)$ [36]. However, strong ϵ-nets are no longer possible if the points of *P* are slightly perturbed in a way that transforms many of the ϵ-heavy hyperplanes *H* into arbitrary thin, albeit strictly convex, sets. Instead, most bounds on point-hyperplane incidences, including the original proof [59] of the tight Szemeredi-Trotter estimate in the plane, stem from the subtler fact that, for any ϵ that is significantly larger than 1/*n*, any *n*-point set in $\mathbb {R}^d$ determines only $o(n^d)$ such (d-1)-simplices whose supporting hyperplanes are “ϵ-heavy” and contain $\Theta (\epsilon n)$ points.

**Fig. 9:**
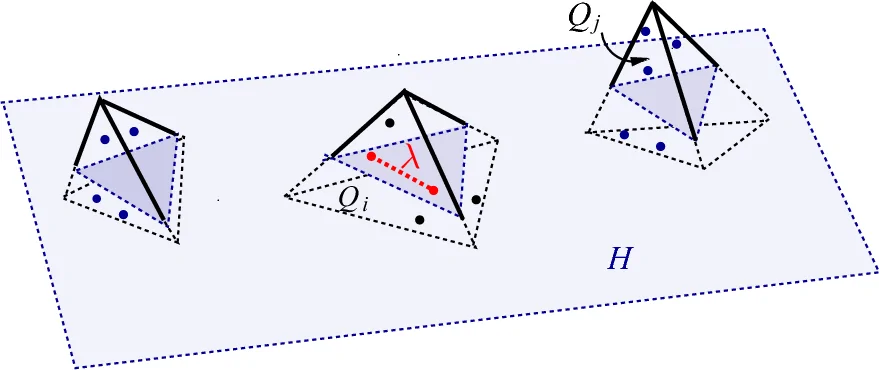
Characterizing a “flat” set *K* in $\mathbb {R}^3$. For a fixed fraction of the short 2-edges $\lambda \in {P_K\atopwithdelims ()2}$, there exists such a hyperplane *H* through λ whose zone in $\mathcal {P}(P,t)$ encompasses almost $\Omega (\epsilon n)$ points of $P_K$

To define a more robust notion of flatness, in the sequel we construct a *d*-dimensional simplicial *t*-partition $\mathcal {P}(P,t)$ of *P*,^17^ with $t=(1/\epsilon )^{3d/4+o_d(1)}=o\left( (1/\epsilon )^{d}\right)$, and take into account the possible “co-planarities” between the *ambient cells* of the points of $P_K$. We say that a 2-edge $\lambda \in {P_K\atopwithdelims ()2}$ is *short* if both of its endpoints fall in the same part $Q\in \mathcal {P}(P,t)$. Informally, an ϵ-heavy convex set *K* will be called *flat* if a fixed fraction of all short 2-edges $\lambda \in {P_K\atopwithdelims ()2}$ lie in hyperplanes $H=H_\lambda$ whose respective zones (in the partition $\mathcal {P}(P,t)$) encompass a large fraction of the points of $P_K$. See Fig. 9.

In what follows, we obtain a subset $\tilde{\Pi }\subseteq {P\atopwithdelims ()d}$ of only $O^*\left( \epsilon ^{d-\alpha _d} n^d\right)$ simplices which, nevertheless, encompasses almost $\Theta \left( \epsilon ^dn^d\right)$ of the simplices within any simplex family ${P_K\atopwithdelims ()d}$ that is induced by a flat convex set $K\in {{\mathcal {K}}}(P,\epsilon )$. Plugging this sparser set $\tilde{\Pi }$, or rather the 1-dimensional cross-sections $\left\{ {{\texttt{conv}}}(\tau )\cap \ell \mid \tau \in \tilde{\Pi }\right\}$ with the canonical lines $\ell \in \mathcal {{L}}$, into our 1-dimensional reduction, will ultimately yield an $O^*\left( 1/\epsilon ^{\alpha _d}\right)$-size net piercing all the flat (and sufficiently spread) sets *K* within ${{\mathcal {K}}}(P,\epsilon )$.

**Piercing “thick” sets.** All the remaining convex sets *K*, whose principal subsets $P_K$ are not sufficiently “co-aligned” with their short edges $\lambda \in {P_K\atopwithdelims ()2}$, will be dispatched through a careful combination of (for most, simpler) nets. For example, the strong net of Haussler and Welzl [36] (see Lemma A.1) can be used to get rid of such convex sets that contain a sufficiently heavy convex “(2*d*)-hedron” – a convex polytope with at most 2*d* vertices. Furthermore, if some part $Q\in \mathcal {P}(P,t)$ contains a relatively large fraction of the points in $P_K$, then the set *K* can be again relegated to a subsidiary recursive sub-problem which is defined over *Q*. On the other hand, if the zone of *K* in the simplicial partition $\mathcal {P}(P,t)$ is relatively “fat” (which happens, e.g., if each part of $\mathcal {P}(P,t)$ contains relatively few points of $P_K$), then the points of $P_K$ can be effectively replaced, for the sake of our 1-dimensional reduction, by the vertices of their ambient cells, again leading to a larger density parameter ν.

#### Piercing δ-Hollow Sets

**Fig. 10:**
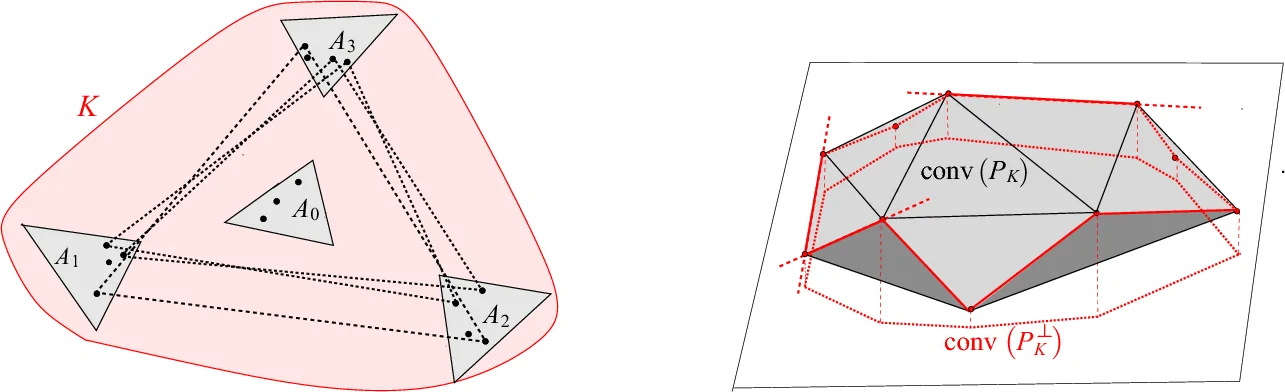
The two classes of convex sets in $\mathbb {R}^3$. Left: The principal subset $P_K$ is δ-punctured (view from above). Hence, it contains 4 subsets $A_0,A_1,A_2,A_3$, of cardinality almost ϵn, which satisfy $p_0^\perp \in {{\texttt{conv}}}(p_1^\perp ,p_2^\perp ,p_3^\perp )$ for all $p_0\in A_0,p_1\in A_1,p_2\in A_2$ and $p_3\in A_3$. Right: The principal subset $P_K$ is 0-hollow; the edges of the silhouette of ${{\texttt{conv}}}(P_K)$ are highlighted, along with their vertical projections. Notice that the directions of the silhouette edges may diverge, even for arbitrary “slim” ${{\texttt{conv}}}(P_K)$

To facilitate the outlined construction, a “typical” convex set $K\in {{\mathcal {K}}}(P,\epsilon )$ will be assigned in Sect. 5 a so called *principal* (d+1)*-tuple* $A_0,A_1,\ldots ,A_{d}\subseteq P_K$ of pairwise disjoint subsets whose respective cardinalities are close to ϵn, and so that $A_0^\perp$ is surrounded, within $\mathbb {R}^{d-1}$, by $A_1^\perp ,\ldots ,A_d^\perp$. (Namely, every choice of d+1 points $p_0\in A_0,\ldots ,p_{d}\in A_{d}$ must yield a punctured set $\{p_0,\ldots ,p_{d}\}$ with $p_{0}^\perp \in {{\texttt{conv}}}\left( p^\perp _1,\ldots ,p^\perp _{d}\right)$, as depicted in Fig. 10 (left).)

However, such a (d+1)-tuple $A_0,\ldots ,A_{d}$ can possibly exist only if the principal set $P_K$ is δ-punctured, where δ>0 is a very small (albeit, positive) power of ϵ that will be determined in the sequel. Indeed, if the set $P_K$ is δ-hollow, then the vast majority of the projected (d+1)-tuples $\left\{ p_1^\perp ,\ldots ,p_{d+1}^\perp \right\} \subseteq P^\perp _K$ are in convex position. Informally, the δ-hollow principal subsets $P_K$ pose a singular challenge to our machinery due to the highly divergent nature of the directions of the short 2-edges of ${{\texttt{conv}}}\left( P_K\right)$ in the vicinity of the vertical silhouette of ${{\texttt{conv}}}(P_K)$; see Fig. 10 (right). As a result, the delicate interplay between the simplices of ${P_K\atopwithdelims ()d}$ and the short 2-edges of ${P_K\atopwithdelims ()2}$, may break down while the convex hull ${{\texttt{conv}}}(P_K)$ remains overly “slim” (and, therefore, difficult to pierce).

To address this problem (which did not arise in $\mathbb {R}^2$), in Sect. 6 we describe a separate net for piercing all such convex set $K\in {{\mathcal {K}}}(P,\epsilon )$ whose principal subsets $P_K$ are δ-hollow. Instead of using the general 1-dimensional reduction, it exploits the structure of the δ-hollow subsets $P_K$, and uses the cutting machinery of Sect. 2.5 to derive a favourable recurrence in the ground set *P* and ϵ>0. This $O\left( \frac{\log r}{r}\right)$-cutting will be constructed by the means of Theorem 2.7 in the arrangement $\mathcal {A}\left( {\mathcal {H}}\right)$ of certain hyperplanes that support the simplices of Pd, and its analysis will invoke Theorem 2.9 to bound the complexity of multiple cells in the arrangement $\mathcal {A}(\mathcal {R})$ of an *r*-sample $\mathcal {R}\in {{\mathcal {H}}\atopwithdelims ()r}$.

#### Fast Forward

**Fig. 11:**
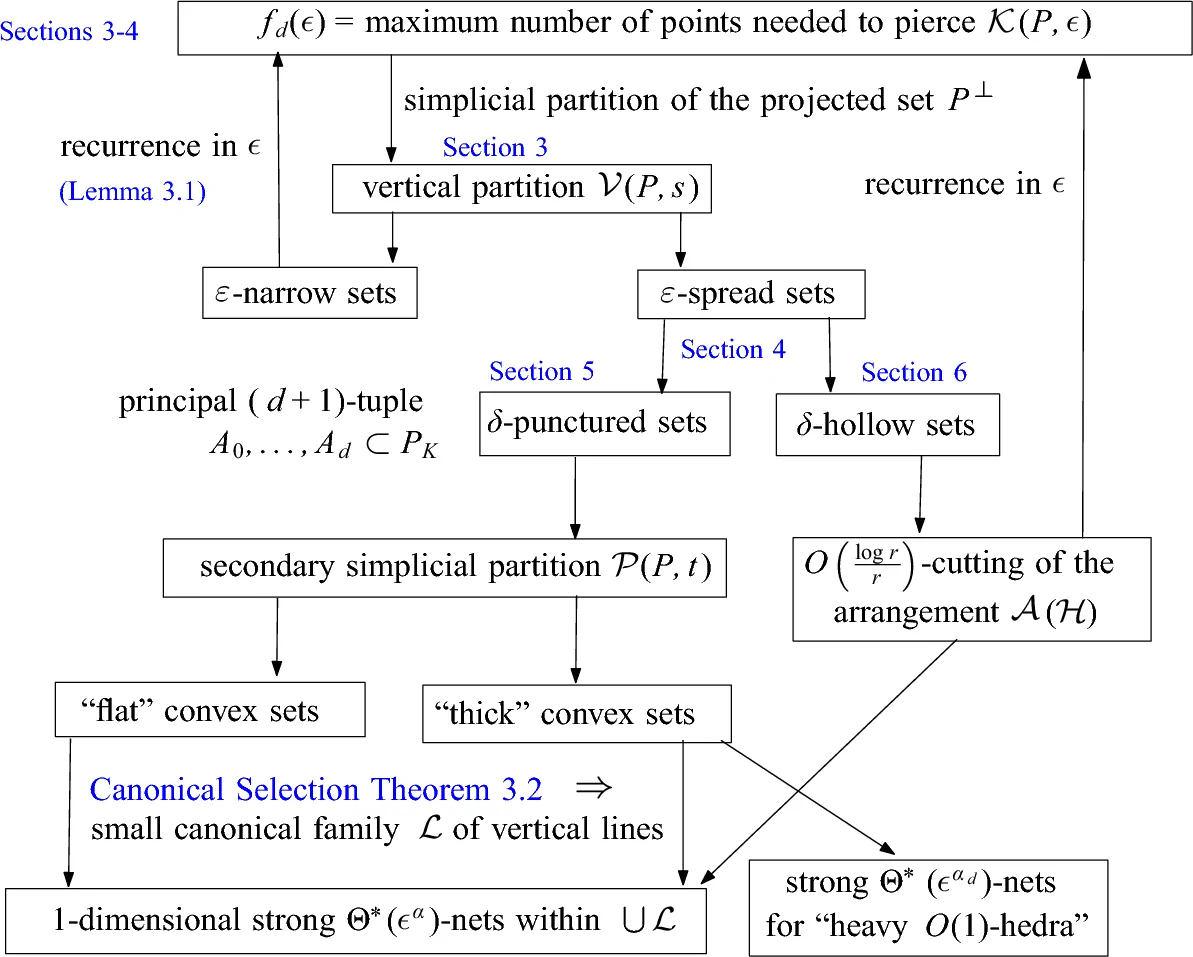
Proof of Theorem 1.1 – a schematic outline of the main steps

The main steps in the proof of Theorem 1.1 are schematically outlined in Fig. 11. In Sect. 3 we formalize the key reduction of the weak ϵ-net problem to a sequence of 1-dimensional nets, including the vertical partition $\mathcal {V}(P,s)$ which underlies the essential distinction between the narrow and well-spread sets of ${{\mathcal {K}}}(P,\epsilon )$. This machinery is then used in Sect. 4 to develop an efficient recurrence for the quantity $f_d(\epsilon )$ and, thereby, establish Theorem 1.1. To facilitate the overall recurrence in Sect. 4, in Sects. 5 and 6 we describe separate nets for the just described key categories of the ϵ-heavy convex sets *K* whose principal subsets $P_K$ are, respectively, δ-punctured and δ-hollow.

## Piercing Many Simplices with Few Canonical Lines

Let us now cast the missing details into the key reduction of the weak ϵ-net problem in dimension d≥3 to a system of 1*-dimensional nets* which are constrained to a *small-size* canonical family $\mathcal {{L}}(P,s)$ of vertical lines. As was emphasized in Sect. 2.7, any such reduction must leave out certain “narrow” sets $K\in {{\mathcal {K}}}(P,\epsilon )$, to be dispatched by a separate recursive construction, at the expense of introducing an additional recursive term of the form $O\left( s\cdot f_d\left( \epsilon \cdot s^{1/(d-1)}\right) \right)$.

### The Vertical Partitions $\mathcal {V}(P,s)$

To quantify the relative distributions of the projected principal subsets $P_K^\perp$, we vertically project the entire point set *P* onto the copy of $\mathbb {R}^{d-1}$, which is spanned by the first *d* coordinates, and construct the simplicial partition of Theorem 2.6 of the projected set $P^\perp \subset \mathbb {R}^d$.

#### Definition

Let *P* be an *n*-point set in a general position in $\mathbb {R}^d$, and *s* an integer that satisfies $1\le s\le n$. We say that $\{(P_i,\Delta _i)\mid 1\le i\le s\}$ is the *vertical* *s-partition* (or, simply, the vertical partition, if the parameter *s* is clear from the context) if $\{(P^\perp _i,\Delta _i)\mid 1\le i\le s\}$ is the simplicial *s*-partition $\mathcal {P}_{d-1}(P^\perp ,s)$ of the projected set $P^\perp$ in $\mathbb {R}^{d-1}$ that was described in Theorem 2.6 (and *uniquely* fixed for each point set *P* and each integer *s* so that $1\le s\le |P|$.).

In particular, every cell $\Delta _i$ is a (d-1)*-dimensional* simplex in $\mathbb {R}^{d-1}$, and every subset $P_i$ is contained in the vertical prism $\Delta ^*_i$ that is raised over the respective (d-1)-dimensional simplicial cell $\Delta _i$.

By definition, for each finite point set $P\subset \mathbb {R}^d$ in general position, and each parameter $1\le s\le |P|$, there is a unique such vertical partition $\{(P_i,\Delta _i)\mid 1\le i\le s\}$, which we denote by $\mathcal {V}(P,s)$. For each $1\le i\le s$, and each point $p\in P_i$, we refer to $\Delta _i$ as *the ambient cell* of *p* in $\mathcal {V}(P,s)$, which we denote by Δ(p).

#### Definition

Let ε>0. We say that a convex set $K\subseteq \mathbb {R}^d$ is ε*-narrow* with respect to the vertical partition $\mathcal {V}(P,s)=\{(P_i,\Delta _i)\mid 1\le i\le s\}$ if there exists a (d-2)-flat *g* within $\mathbb {R}^{d-1}$ whose zone within the respective (d-1)-dimensional partition $\mathcal {P}_{d-1}(P^\perp ,s)=\{(P_i^\perp ,\Delta _i)\mid 1\le i\le s\}$ encompasses at least εn of the projected points of $(P\cap K)^\perp$. See Fig. 8 (left). If the convex set $K\subseteq \mathbb {R}^d$ is not ε-narrow with respect to a vertical partition $\mathcal {V}(P,s)$, we say that *K* is ε*-spread* in $\mathcal {V}(P,s)$.^18^ (See Fig. 8 (right).)

As a rule, the narrowness threshold ε will be either $\Theta (\epsilon )$, or only marginally smaller than the parameter ϵ in Theorem 1.1.

### Piercing the ε-Narrow Convex Sets

As was mentioned in Sect. 2.7, the simplices in the ε-narrow sets of ${{\mathcal {K}}}(P,\epsilon )$ may prove hard for interception with few vertical lines. Fortunately, a sizable fraction of their points can be localized in only $O\left( s^{1-1/(d-1)}\right)$ parts $P_i$ of the vertical partition $\mathcal {V}(P,s)$. Hence such sets can still be pierced by a net whose cardinality is bounded by an essentially “$O\left( 1/\epsilon ^{d-1}\right)$-type” recursive term which is close to $O\left( s\cdot f\left( \epsilon \cdot s^{1/(d-1)}\right) \right)$.

#### Lemma 3.1

Let *P* be a finite point set in $\mathbb {R}^d$, and ε>0. Let s>0 be an integer that satisfies $1\le s\le |P|$, and $\mathcal {V}(P,s)$ be the vertical *s*-partition of *P* as described in Sect. 3.1. Then there exists a set $N_{nar}(P,s,\varepsilon )\subset \mathbb {R}^d$, of cardinality $O\left( s\cdot f\left( \varepsilon \cdot s^{1/(d-1)}\right) \right)$, that pierces every convex set *K* that is ε-narrow with respect to $\mathcal {V}(P,s)$.^19^

#### Proof

For every $1\le i\le s$ we construct a net $N_i:=N\left( P_i,\tilde{\epsilon }\right)$ for the local problem ${{\mathcal {K}}}\left( P_i,\tilde{\epsilon }\right)$, where

$$ \tilde{\epsilon }:=c\cdot \varepsilon {s^{1/(d-1)}}=\Theta \left( \frac{\varepsilon n/\left( s^{1-1/(d-1)}\right) }{n/s}\right) , $$

and c>0 is a suitably small fixed constant that does not depend on ϵ, ε or *s* (but may depend on *d*. Notice that each $N_i$ is a *d*-dimensional net of cardinality $|N_i|\le f\left( \tilde{\epsilon }\right)$.

It suffices to show that the union $N_{nar}(P,s,\varepsilon ):=\bigcup _{1\le i\le s} N_i$ pierces all the ε-narrow convex sets. Indeed, let *K* be such a narrow convex set with respect to $\mathcal {V}(P,s)$, and *g* be a “witness” (d-2)-flat within $\mathbb {R}^{d-1}$ that certifies the ε-narrowness of *K*. That is, at least εn points of P∩K belong to such sets $P_i$ whose ambient (d-1)-dimensional cells $\Delta _i\subset \mathbb {R}^{d-1}$ are crossed by *g*. Since the number of the latter simplices is only $O\left( s^{1-1/(d-1)}\right)$ (via Theorem 2.6), the pigeonhole principle yields such a set $P_i$ that contains $\Omega \left( \varepsilon n/\left( s^{1-1/(d-1)}\right) \right)$ of the points of P∩K. A suitable choice of the constant c>0 guarantees that *K* is pierced by the respective net $N_i$. □

### The Canonical Line-Selection Theorem

Specializing to the remaining sets, which are not pierced by the recursive net of Sect. 3.2, we obtain a so called *canonical set* $\mathcal {{L}}=\mathcal {{L}}(P,s)$ of $s^{O\left( d^2\right) }$ vertical lines, with the following property: for any $\Omega ^*(\epsilon )$-spread convex set $K\in {{\mathcal {K}}}(P,\epsilon )$, there exists a subset of roughly $\Omega \left( \epsilon ^dn^d\right)$ simplices of ${P_K\atopwithdelims ()d}$ that can be crossed by a single line of $\mathcal {{L}}$. Before we proceed with the more formal definition of such canonical families $\mathcal {{L}}$ of lines, and the proof of their existence, further notation is in order.

#### Definition

We say that a vertical line ℓ in $\mathbb {R}^d$ is *surrounded* by some *d* sets $\Delta _1,\ldots ,\Delta _d\subseteq \mathbb {R}^{d-1}$ if the vertical intercept $\ell ^\perp$ of ℓ in $\mathbb {R}^{d-1}$ is surrounded by $\Delta _1,\ldots ,\Delta _d$ according to the definition in Sect. 2.2.

Note that, if a vertical line ℓ is surrounded by some d-1 cells $\Delta _{i_1},\ldots ,\Delta _{i_d}\subset \mathbb {R}^{d-1}$ that arise in the vertical *r*-partition $\mathcal {V}(P,r)$ of Sect. 3.1, for $1\le i_1\ne i_2\ne \ldots \ne i_d\le r$, then it pierces the interior of *every* simplex ${{\texttt{conv}}}(p_1,\ldots ,p_d)$ with $p_j\in P_{i_j}$.

#### Definition

Let ϑ>0, and let $\beta _{d-1}$ denote the constant selection exponent that suits Theorem 3.4 in dimension d-1. (One can choose $0<\beta _{d-1}\le d^5+O(d^4)$ [54].)

**Fig. 12:**
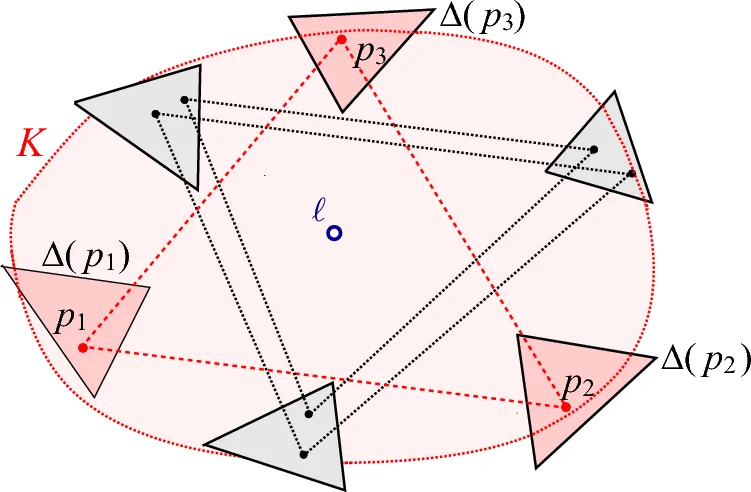
The Canonical Line-Selection Theorem in dimension d=3 (view from above). The set $K\in {{\mathcal {K}}}(P,\epsilon )$ is $(\vartheta \sigma ^{\beta _{d-1}}\epsilon )$-spread in the vertical partition $\mathcal {V}(P,s)$. The ϑ-canonical family $\mathcal {{L}}(P,s)$ contains a vertical line ℓ piercing at least $\vartheta \sigma ^{\beta _{2}}{\lceil \epsilon n\rceil \atopwithdelims ()3}$ simplices $\{p_1,p_2,p_3\}\in \Pi _K$, such that the ambient cells $\Delta (p_1),\Delta (p_2),\Delta (p_3)\subset \mathbb {R}^2$ of their vertices $p_1,p_2$, and $p_3$ surround ℓ

Let *P* be a set of *n* points in general position in $\mathbb {R}^d$, and $1\le s\le n$. We say that a family $\mathcal {{L}}$ of vertical lines is ϑ*-canonical* with respect to the vertical partition $\mathcal {V}(P,s)$ if it satisfies the following property:

*For any* $0\le \epsilon ,\sigma \le 1$, *any* $(\vartheta \sigma ^{\beta _{d-1}}\epsilon )$*-spread set* $K\in {{\mathcal {K}}}(P,\epsilon )$ *with a principal subset* $P_K$ *of exactly* $\lceil \epsilon n\rceil$ *points, and any subset* $\Pi _K\subseteq {P_K\atopwithdelims ()d}$ *of at least* $\sigma {\lceil \epsilon n\rceil \atopwithdelims ()d}$ (d-1)*-simplices, there is a line* $\ell \in \mathcal {{L}}$ *with a subset* $\Pi _K(\ell )$ *of at least* $\vartheta \sigma ^{\beta _{d-1}}{\lceil \epsilon n\rceil \atopwithdelims ()d}$ *simplices within* $\Pi _K$, *that are pierced by* ℓ. *Furthermore, for every simplex* $\{p_1,\ldots ,p_d\}\in \Pi _{K}(\ell )$, *this line* ℓ *is surrounded by the* *d* *ambient cells* $\Delta (p_1),\ldots ,\Delta (p_d)$ *in* $\mathcal {V}(P,s)$. See Fig. 12.

#### Theorem 3.2

(*The Canonical Selection Theorem*) There is a constant ϑ>0 so that for any finite point set *P*, and any $1\le s\le |P|$, there exists a ϑ-canonical set $\mathcal {{L}}$ of $O\left( s^{(d-1)^2}\right)$ vertical lines with respect to $\mathcal {V}(P,s)$. Furtheremore, such a set $\mathcal {{L}}=\mathcal {{L}}(P,s)$ can be constructed from $\mathcal {V}(P,s)$ in time $O\left( s^{(d-1)^2}\right)$.

In what follows, every finite point set *P*, and every integer *s* that satisfies $1\le s\le |P|$, will be assigned a unique ϑ-canonical family $\mathcal {{L}}(P,s)$.

Before proceeding with the proof of Theorem 3.2, let us demonstrate its usefulness to piercing the finer “edge-constrained” sub-families ${{\mathcal {K}}}={{\mathcal {K}}}(P,\Pi ,\epsilon ,\sigma )$, which have been introduced in Sect. 2.6. To this end, the subsets $\Pi _K$ will be set to $\Pi \cap {P_K\atopwithdelims ()d}$, which clearly guarantees that $|\Pi _K|\ge \sigma {\lceil \epsilon n\rceil \atopwithdelims ()d}$ holds for all $K\in {{\mathcal {K}}}$. Therefore, specializing to the $(\vartheta \sigma ^{\beta _{d-1}}\epsilon )$-spread sets *K*, the complete collection Pd of the (d-1)-dimensional simplices can be replaced, for the sake of our 1-dimensional reduction, by its potentially sparser subset Π. Combined with the use of Lemma 3.1 for the $(\vartheta \sigma ^{\beta _{d-1}}\epsilon )$-narrow sets, this yields a more accurate recursive bound on the quantity $f(\epsilon ,\rho ,\sigma )$ whenever the density parameter ρ is significantly smaller than 1 (and the restriction threshold σ remains near-constant).

#### Theorem 3.3

Let $0<\epsilon , \sigma \le 1$, *P* be an *n*-point set in $\mathbb {R}^d$, *s* an integer that satisfies $1\le s\le n$, and $\Pi \subseteq {P\atopwithdelims ()d}$. Then the family ${{\mathcal {K}}}(P,\Pi ,\epsilon ,\sigma )$ can be pierced by a net of size

4 $$\begin{aligned} O\left( \frac{s^{(d-1)^2}|\Pi |}{\sigma ^{\beta _{d-1}}\epsilon ^dn^d}+s\cdot f\left( \sigma ^{\beta _{d-1}}s^{1/(d-1)}\cdot \epsilon \right) \right) . \end{aligned}$$

Thus, we have that

$$ f(\epsilon ,\rho ,\sigma )=O\left( \frac{s^{(d-1)^2}\rho }{\sigma ^{\beta _{d-1}}\epsilon ^d}+s\cdot f\left( \sigma ^{\beta _{d-1}}s^{1/(d-1)}\cdot \epsilon \right) \right) $$

for all $0<\epsilon ,\sigma \le 1$, $0\le \rho \le 1$, and $1\le s\le 1/\epsilon$.

#### Remark

In particular, for *s* that is an arbitrarily small (albeit fixed) power of 1/ϵ, $\rho =\epsilon$, and $\sigma =\Theta (1)$, Theorem 3.3 yields a “$1/\epsilon ^{d-1}$-type” estimate $f(\epsilon ,\epsilon ,\sigma )=O^*\left( \frac{1}{\epsilon ^{d-1}}+s\cdot f\left( s^{1/(d-1)}\cdot \epsilon \right) \right)$.

#### Proof of Theorem 3.3

If $n\le 1/\epsilon$, then the entire family ${{\mathcal {K}}}(P,\epsilon )$ is pierced by the net N=P. Hence, the second part of the theorem is an immediate corollary of its first part.

To see the first part, we construct the vertical partition $\mathcal {V}(P,s)\!\!=\!\! \{(P_1,\Delta _1),\ldots , (P_s,\Delta _s)\}$ that was described in Sect. 3.2. We then invoke Lemma 3.1, with $\varepsilon =\vartheta \sigma ^{\beta _{d-1}}\epsilon$, to construct a net $N_{nar}(P,s,\varepsilon )$ of cardinality $O\left( s\cdot f\left( \varepsilon \cdot s^{1/(d-1)}\right) \right)$, and that pierces all the ε-narrow sets $K\in {{\mathcal {K}}}(P,\Pi ,\epsilon ,\sigma )$ with respect to $\mathcal {V}(P,s)$. (To this end, we stick with the same constant ϑ>0 as in Theorem 3.2.)

Applying the line-selection of Theorem 3.2 to $$\mathcal {V}(P,s)$$ yields a ϑ-canonical family $$\mathcal {{L}}=\mathcal {{L}}(P,s)$$ of at most $$s^{(d-1)^2}$$ vertical lines. For each line $$\ell \in \mathcal {{L}}(P,s)$$ we construct the set $$X_\ell :=\{{{\texttt{conv}}}(\tau )\cap \ell \mid \tau \in \Pi \}$$, which consists of at most |Π| intersection points of ℓ with the simplices of Π, and then select a 1-dimensional strong ν-net $$N_\ell $$ over the set $$X_\ell $$ with respect to intervals, with $$\nu =c\sigma ^{\beta _{d-1}}\epsilon ^d n^d/|\Pi |$$. Here c>0 is a suitably small constant that may depend on *d* (but not on ϵ). Lastly, we define $$N':=(\bigcup _{\ell \in \mathcal {{L}}(P,s)}N_\ell )\cup N_{nar}(P,s,\varepsilon )$$. Since the cardinality of $N^{\prime }$ is obviously bounded by (4), it suffices to show that every convex set $$K\in {{\mathcal {K}}}(P,\Pi ,\epsilon ,\sigma )$$ is pierced by $N^{\prime }$.

Indeed, if *K* is ε-spread with respect to $\mathcal {V}(P,s)$, there must be a vertical canonical line $\ell \in \mathcal {{L}}(P,s)$ that pierces $\Omega \left( \sigma ^{\beta _{d-1}}{\lceil \epsilon n\rceil \atopwithdelims ()d}\right)$ simplices of $\Pi _K:=\Pi \cap {P_K\atopwithdelims ()d}$, each time at some point of $X_\ell$ within the segment $K\cap \ell$. Therefore, a suitable choice of the constant c>0 guarantees that *K* is pierced by the respective net ν-net $N_\ell \subset N'$.

On the other hand, if *K* is ε-narrow with respect to $\mathcal {V}(P,s)$, then it must be pierced by the net $N_{nar}(P,s,\varepsilon )$ of Lemma 3.1. □

### A Useful Lemma

The proof of Theorem 3.2 relies on the following multiset variant of Theorem 2.5.

#### Lemma 3.4

Let (*V*, *E*) be a (d+1)-uniform hypergraph so that |V|=n and $|E|\ge h{n\atopwithdelims ()d+1}$, and $\chi :V\rightarrow \mathbb {R}^d$ be a mapping of *V* to some point set X=χ(V) in general position in $\mathbb {R}^d$.^20^ Then there exists a point $x\in \mathbb {R}^d$ that satisfies the following conditions:

1. There exist $\Omega \left( h^{\beta _d}{n\atopwithdelims ()d+1}\right)$ hyperedges f∈E so that $x\in {{\texttt{conv}}}\left( \chi (f)\right)$.
2. There exist k≤d pairwise disjoint subsets $A_1,\ldots ,A_k\subseteq X$, each of cardinality $|A_i|\le d$, with the property that $\{x\}=\bigcap _{i=1}^k{{\texttt{conv}}}(A_i)$.

#### Proof

We apply an arbitrary small generic perturbation to the mapping $$\chi : V\rightarrow \mathbb {R}^d$$. To this end, we fix a sufficiently small δ>0, and replace the image χ(v) of every vertex v∈V by a point $\chi^{\prime }\left(v\right)$ that is chosen uniformly, independently, and at random from the δ-neighborhood $$B(\chi (v),\delta )$$. Then, with probability 1, the perturbed image $\chi^{\prime }\left(V\right)$ is comprised of *n* distinct points in general position. Thus, Theorem 2.5 yields a point $$x\in \mathbb {R}^d$$, with $$\Omega \left( h^{\beta _d}{n\atopwithdelims ()d+1}\right) $$ edges f∈E, so that $$x\in {{\texttt{conv}}}(\chi '(f))$$. Due to an arbitrary small choice of δ>0, the standard compactness argument yields such a point *x* that meets condition (1) with respect to the original mapping χ.

Let $E^{\prime }$ denote the subset of all such edges f∈E that satisfy $$x\in {{\texttt{conv}}}(\chi (f))$$. Then it can be assumed, with no loss of generality, that *x* is a vertex of the polytope $$\bigcap _{f\in E'}{{\texttt{conv}}}\left( \chi (f)\right) $$. As such, it is an intersection of some *k* boundary faces $$\mu _1,\ldots ,\mu _k$$ of some *k* distinct simplices $$\tau _1,\ldots ,\tau _k\in \{{{\texttt{conv}}}(\chi (f))\mid f\in E'\}$$ whose dimensions may vary between 0 and d-1. Choosing the minimal such representation (which involves the smallest possible overall number of points) guarantees that the faces $$\mu _1,\ldots ,\mu _k$$ have pairwise disjoint vertex sets, which we respectively denote by $$A_1,\ldots ,A_k$$, so that $$x=\bigcap _{i=1}^k {{\texttt{conv}}}(A_i)$$. □

### Proof of Theorem 3.2

We fix an *n*-point set *P* with an integer *s* that satisfies $1\le s\le n$, which together determine the vertical *s*-partition $\mathcal {V}(P,s)$ of *P*.

**Constructing the canonical line family** $\mathcal {{L}}(P,s)$. We pick a generic proxy point $x_i\in \mathbb {R}^{d-1}$ within every (d-1)-dimensional cell $\Delta _i$ of the vertical partition $\mathcal {V}(P,s)$. This yields the set $X:=\{x_i\mid 1\le i\le s\}$, whose *s* points in general position will be used to generate a significantly larger point set *Y* within $\mathbb {R}^{d-1}$.

**Fig. 13:**
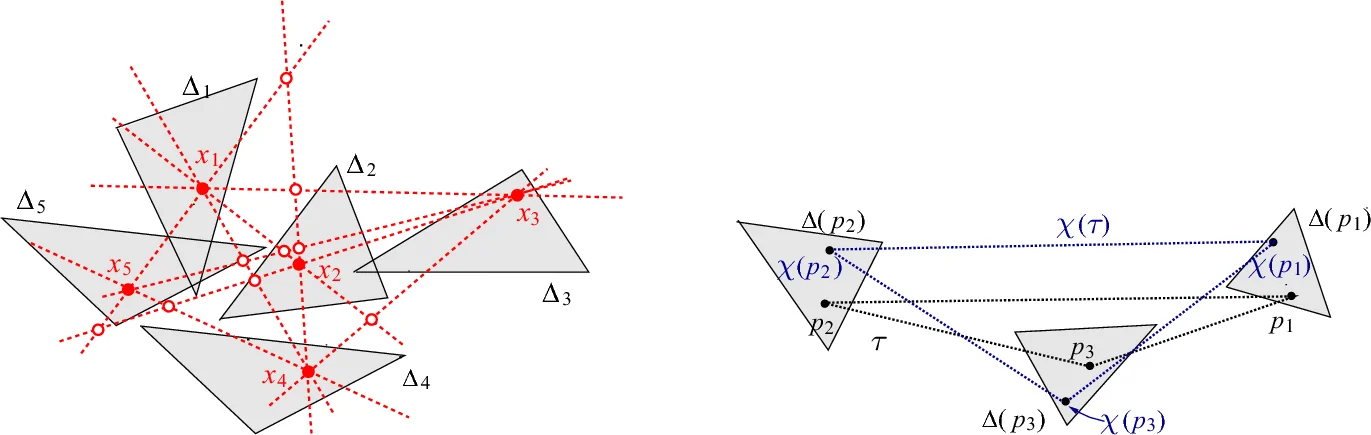
Proof of Theorem 3.2 in dimension d=3. Left: The set $X=\{x_1,\ldots ,x_r\}$ with (part of) the induced set *Y* in $\mathbb {R}^2$. Right: The triangle $\tau ={{\texttt{conv}}}(p_1,p_2,p_3)$ in the vertical projection, and its representative $\chi (\tau )$ under the mapping $\chi : P_K\rightarrow \mathbb {R}^2$

To this end, for every $1\le k\le d-1$, and every choice $A_1,\ldots ,A_k$ of k≤d-1 non-empty and pairwise disjoint sets $A_1,\ldots ,A_k\in {X\atopwithdelims ()\le (d-1)}$ that satisfy $\sum _{i=1}^k(d-|A_i|)=d-1$ and $\bigcap _{i=1}^d{{\texttt{conv}}}(A_i)\ne \emptyset$, we add to *Y* the unique point *y* so that $\{y\}=\bigcap _{i=1}^k {{\texttt{conv}}}(A_i)$; see Fig. 13 (left).^21^

We then set $\mathcal {{L}}(P,s):=\{y^*\mid y\in Y\}$. Namely, $\mathcal {{L}}(P,s)$ is the set of all the vertical lines that result from lifting the points of *Y*.

**Analysis.** By definition, we have $|\mathcal {{L}}(P,s)|=|Y|=O\left( s^{(d-1)^2}\right)$, so it remains to show that, given a suitably small constant ϑ>0, the family $\mathcal {{L}}(P,s)$ is ϑ-canonical with respect to $\mathcal {V}(P,s)$. To this end, let us fix the parameters $0<\epsilon ,\sigma \le 1$. Let $K\in {{\mathcal {K}}}(P,\epsilon )$ be a convex set which is endowed with a principal set $P_K\subseteq P$ of $\lceil \epsilon n\rceil$ points, and a subset $\Pi _K\subseteq {P_K\atopwithdelims ()d}$ of at least $\sigma {\lceil \epsilon n\rceil \atopwithdelims ()d}$ (d-1)-simplices.

To show that $\mathcal {{L}}(P,s)$ is ϑ-canonical, with some ϑ>0 that depends only on *d*, it is enough to show that at least one of the following statements holds with the previous choice of *K*, $P_K$, and $\Pi _K$:

(i) there is a line $\ell \in \mathcal {{L}}(P,s)$ that pierces the relative interiors of at least $\Omega \left( \sigma ^{\beta _{d-1}}{|P_K|\atopwithdelims ()d}\right)$ simplices $\tau \in \{p_1,\ldots ,p_d\}\in \Pi _K$ whose ambient cells $\Delta (p_i)$ surround ℓ, or
(ii) the set *K* is $\Omega \left( \sigma ^{\beta _{d-1}}\epsilon \right)$-narrow in $\mathcal {V}(P,s)$.

To this end, it can be henceforth assumed that $|P_K|\ge d$ (or, else, the claim holds emptily with ${P_K\atopwithdelims ()d}=\Pi _K=\emptyset$).

*The mapping* $\chi :P_K\rightarrow X$. For each $1\le i\le s$, and each $p\in P_i\cap P_K$, we map every point $p\in P_K$ to the proxy point $\chi (p)=x_i$ within the ambient cell $\Delta _i=\Delta (p)\subseteq \mathbb {R}^{d-1}$. See Fig. 13 (right). As a result, every simplex $\tau =\{p_1,\ldots ,p_d\}\in \Pi _K$ maps to the set $\chi (\tau )=\{\chi (p_i)\mid 1\le i\le d\}\in {X\atopwithdelims (){\le d}}$. We say that this simplex $\tau \in \Pi _K$ is *crowded* if $|\chi (\tau )|<d$, and we say that τ is *split* otherwise.

**Case 1.** At least $|\Pi _K|/3$ of the hyperedges in $\Pi _K$ are crowded. The pigeonhole principle yields a subset $\kappa =\{p_1,\ldots ,p_{d-1}\}\in {P_K\atopwithdelims ()d-1}$ with at least $|\Pi _K|/\left( 3{\lceil \epsilon n\rceil \atopwithdelims ()d-1}\right)$ hyperedges $\tau =\{p_1,\ldots ,p_{d-1},p\}\in \Pi _K$ that satisfy $\chi (\tau )=\chi (\kappa )$. Up to relabeling, it can be assumed that at least $|\Pi _K|/\left( 3d{\lceil \epsilon n\rceil \atopwithdelims ()d-1}\right) =\Omega \left( \sigma \epsilon n\right)$ of the latter simplices τ satisfy $\chi (p)=\chi (p_1)$. In other words, the ambient subset $P_i$ of $p_1$ in $\mathcal {V}(P,s)$ must encompass $\Omega (\sigma \epsilon n)$ points $p\in P_K$. Hence, the set *K* must be $\Omega (\sigma \epsilon )$-narrow in $\mathcal {V}(P,s)$.

**Case 2.** Assume then that the previous scenario does not occur for *K* and $\Pi _K$. Let $\Pi '_K$ denote the subset of all the split simplices in $\Pi _K$, which now must encompass at least $2|\Pi _K|/3$ simplices. Applying Lemma 3.4 for the *d*-uniform hypergraph $(P_K,\Pi '_K)$, with the previous embedding function $\chi :P_K\rightarrow X\subseteq \mathbb {R}^{d-1}$, yields a point y∈Y and a subset $\Pi '_K(y)\subseteq \Pi '_K$ of $\Omega \left( \sigma ^{\beta _{d-1}}{|P_K|\atopwithdelims ()d}\right)$ simplices within $\Pi '_K$, with the property that $y\in {{\texttt{conv}}}\left( \chi (\tau )\right)$ for each $\tau \in \Pi '_K(y)$.

**Fig. 14:**
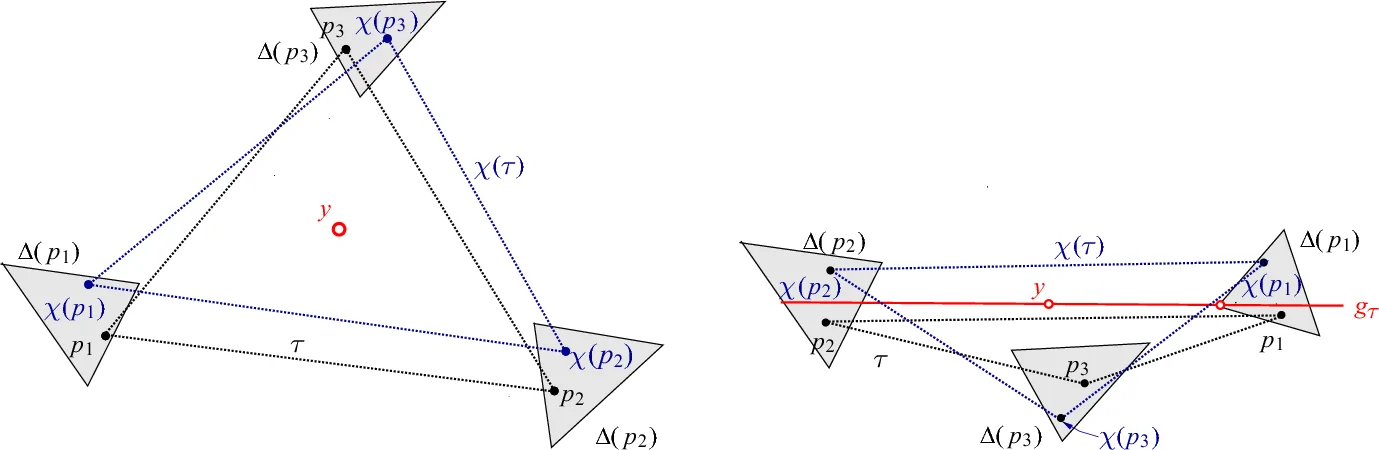
Proof of Theorem 3.2 (in dimension d=3). Left: Case (2a) – the simplex $\tau ={{\texttt{conv}}}(p_1,p_2,p_3)$ (shown in the vertical projection) belongs to $\Pi ''_K(y)$, so that *y* is surrounded by $\Delta (p_1),\Delta (p_2)$, and $\Delta (p_3)$; in particular, τ is crossed by the vertical line $\ell =y^*$ over *y*. Right: Case (2b) – the simplex $\tau ={{\texttt{conv}}}(p_1,p_2,p_3)$ belongs to $\Pi '_K(y)\setminus \Pi ''_K(y)$, so that *y* is not surrounded by $\Delta (p_1),\Delta (p_2)$, and $\Delta (p_3)$. The line $g_\tau$ passes through *y* and a vertex of $\Delta (p_1)$, and crosses $\Delta (p_2)$

Let $\Pi ''_K(\ell )\subseteq \Pi '_K(y)$ denote the subset of all the (d-1)-simplices $\tau =\{p_1,\ldots ,p_d\}$ in $\Pi '_K(y)$, so that *y* is surrounded by the ambient cells $\Delta (p_1),\ldots ,\Delta (p_d) \subset \mathbb {R}^{d-1}$ of their *d* vertices $p_1,\ldots ,p_d$. To complete the proof of Theorem 3.2, we distinguish between two possible scenarios (see Fig. 14).

**Case 2a.** If $|\Pi ''_K(y)|\ge |\Pi '_K(y)|/2=\Omega \left( \sigma ^{\beta _{d-1}}{|P_K\atopwithdelims ()d}\right)$ then $y^*$ constitutes the desired canonical line in $\mathcal {{L}}(P,s)$ (with a suitably small choice of ϑ>0).

**Case 2b.** Suppose, then, that the set $\Pi '_K(y)\setminus \Pi ''_K(y)$ encompasses at least $|\Pi '_K(y)|/2=\Omega \left( \sigma ^{\beta _{d-1}}{|P_K|\atopwithdelims ()d}\right)$ simplices. It suffices to show that such a convex set *K* must be $\Omega \left( \sigma ^{\beta _{d-1}}\epsilon \right)$-narrow in $\mathcal {V}(P,s)$.

To this end, we fix $\tau ={{\texttt{conv}}}\left( p_1,\ldots ,p_d\right) \in \Pi '_K(y)\setminus \Pi ''_K(y)$. Notice that the point *y* is *not* surrounded in $\mathbb {R}^{d-1}$ by the *d* ambient cells $\Delta (p_1),\ldots ,\Delta (p_d)$ of the vertices of τ, and yet it lies within ${{\texttt{conv}}}\left( \bigcup _{i=1}^d \Delta (p_i)\right)$ (and, specifically, within the simplex ${{\texttt{conv}}}(\chi (\tau ))$, whose vertices $\chi (p_i)$ are chosen from the respective cells $\Delta (p_i)$). Hence, Lemma 2.2 yields a (d-2)-flat *g* through *y*, within $\mathbb {R}^{d-1}$, that intersects some d-1 of the cells $\Delta (p_i)$, say $\Delta (p_1),\Delta (p_2),\ldots ,\Delta (p_{d-1})$. Furthermore, applying Lemma 2.3 with $\Sigma =\{\Delta (p_1),\ldots ,\Delta (p_{d-1}),\{y\}\}$ yields such a (d-2)-flat $g=g_\tau$ that contains *y* and, in addition, passes through some d-2 boundary vertices of $\Delta (p_1),\Delta (p_2),\ldots ,\Delta (p_{d-1})$.

Notice that the supporting vertices of $g_\tau$ belong to at most d-2 simplices which, with no loss of generality, can be assumed to be amongst $\Delta (p_1),\ldots ,\Delta (p_{d-2})$. Also note that, for a fixed y∈Y, the (d-2)-flat $g_\tau$ can be “guessed” (using Lemma 2.3) from $p_1,\ldots ,p_{d-2}$ in at most $${\left( \begin{array}{lll}(d-2)d\\ d-2\end{array}\right) }$$ different ways from among the vertices of the d-2 ambient cells $\Delta (p_i)$. We, therefore, assign to τ this characteristic (d-2)-subset $S_\tau =\{p_1,\ldots ,p_{d-2}\}\subset P_K$.

We repeat the above argument for each simplex $\tau \in \Pi '_K(y)\setminus \Pi ''_K(y)$. Since the overall number of distinct characteristic subsets $S_\tau$, over all the simplices $\tau \in \Pi '_K(y)\setminus \Pi ''_K(y)$, is at most ${\lceil \epsilon n \rceil \atopwithdelims ()d-2}$, by the pigeonhole principle there must be such a (d-2)-subset $S\in {P_K\atopwithdelims ()d-2}$ that has been assigned to $\Omega \left( \sigma ^{\beta _{d-1}}{{\lceil \epsilon n\rceil }\atopwithdelims ()2}\right)$ of the (d-1)-simplices $\tau \in \Pi '_K(y)\setminus \Pi ''_K(y)$ (which all satisfy $S_\tau =S$). For each of these simplices τ, one or both of the two vertices in $\tau \setminus S$ must belong to such partition sets $P_i$ whose ambient cells $\Delta _i$ are intersected (in $\mathbb {R}^{d-1}$) by the (d-2)-flat $g_\tau$. Since the (d-2)-flat $g_\tau$ can be chosen via Lemma 2.3 from among $${\left( \begin{array}{lll}d(d-2)\\ d-2\end{array}\right) }=O(1)$$ possible candidates, at least $\Omega \left( \sigma ^{\beta _{d-1}}{{\lceil \epsilon n\rceil }\atopwithdelims ()2}\right)$ of these latter simplices τ must also “share” the same (d-2)-flat $g=g_\tau$. Using that any point of $P_K$ belongs to at most $\lceil \epsilon n \rceil$ distinct pairs $\tau \setminus S$, with $\tau \in \Pi '_K(y)\setminus \Pi ''_K(y)$, we conclude that the projections of at least $\Omega \left( \sigma ^{\beta _{d-1}}\lceil \epsilon n\rceil \right)$ points in $P_K$ must fall, within the partition $\mathcal {P}_{d-1}(P^\perp ,s)=\{(P_1^\perp ,\Delta _1),\ldots ,(P_s^\perp ,\Delta _s)\}$, in the zone of the *same* (d-2)-flat *g*. In other words, the set *K* must be $\Omega \left( \sigma ^{\beta _{d-1}}\epsilon \right)$-narrow. □

## Proof of Theorem 1.1

In this section we will use the machinery that has been assembled in Sects. 2 and 3, in order to establish the main result of this paper – Theorem 1.1. To this end, we fix an arbitrary constant γ>0, and derive a complete recurrence formula for the quantity $f(\epsilon )=f_d(\epsilon )$, which will ultimately solve to $f(\epsilon )=O\left( 1/\epsilon ^{\alpha _d+\gamma }\right)$. It can, furthermore, be assumed with no loss of generality that γ<1/100.

To facilitate the asymptotic analysis of the weak ϵ-numbers f(ϵ), we let $\eta :=\gamma ^{2d}/(100d)$, and adopt the following useful notation. For y>1, we denote $x\lll _\eta y$ whenever $x=O\left( y^{\frac{\eta }{100d^4\beta _{d-1}}}\right)$, where $\beta _{d-1}\le d^5(1+o(1))$ denotes the fixed selection exponent that Theorem 2.5 yields in dimension d-1. For 0<y<1, we accordingly denote $x\lll _\eta y$ whenever $1/y\lll _\eta 1/x$.

### The Setup

As was mentioned in Sect. 2.6, we first develop an efficient recurrence for the more general quantity $f(\epsilon ,\rho ,\sigma )$, with $0<\epsilon ,\sigma ,\rho \le 1$. Recall that $f(\epsilon ,\sigma ,\rho )$ denotes the smallest possible number of points that would suffice to pierce *any* “edge-constrained” family ${{\mathcal {K}}}={{\mathcal {K}}}(P,\Pi ,\epsilon ,\sigma )$ which is determined by a finite point set *P* in general position, and a subset $\Pi \subseteq {P\atopwithdelims ()d}$ of at most $\rho {|P|\atopwithdelims ()d}$ (d-1)-dimensional simplices.

Unless stated otherwise, in what follows we will use $\Pi _K$ to denote the sets ${P_K\atopwithdelims ()d}\cap \Pi$ that are induced by the elements of ${{\mathcal {K}}}$; hence, every family ${{\mathcal {K}}}={{\mathcal {K}}}(P,\Pi ,\epsilon ,\sigma )$ under consideration is comprised of all the ϵ-heavy convex sets $K\in {{\mathcal {K}}}(P,\epsilon )$ that satisfy $|\Pi _K|\ge \sigma {|P_K|\atopwithdelims ()d}$.

It can be assumed, with no loss of generality, that ϵ>0 is bounded from above by a sufficiently small absolute constant $\epsilon _0=\epsilon (\gamma ,d)>0$, which is smaller than 1/100.^22^

Otherwise, if $\epsilon \ge \epsilon _0$, we can use, e.g., the bound $f(\epsilon ,\rho ,\sigma )\le f(\epsilon )=O\left( 1/\epsilon _0^{d+1}\right) =O(1)$ due to Alon et al. [1].

In addition to $\epsilon ,\rho$ and σ, our recurrence formula for $f(\epsilon ,\rho ,\sigma )$ will involve five integer parameters *h*, *r*, *s*, *u*, and *t*, which depend on ϵ>0 (and also on the constants γ and *d*). Specifically, *t* assumes a fixed power $t=\Theta \left( 1/\epsilon ^{\theta _d}\right)$ of 1/ϵ which satisfies $\Big \lceil 1/\epsilon ^{d/2}\Big \rceil \le t\le \Big \lceil 1/\epsilon ^{\alpha _d}\Big \rceil$, and it will be set in what follows to $(1/\epsilon )^{3d/4+o_d(1)}$ so as to “optimize” the eventual recurrence for $f(\epsilon ,\rho ,\sigma )$. The remaining four parameters, *h*, *r*, *s* and *u*, are very small (positive) powers of 1/ϵ that satisfy

$$ h\lll _\eta r\lll _\eta s\lll _\eta u \lll _\eta 1/\epsilon . $$

Specifically, we denote $\tilde{\eta }:=\eta /(100d^4\beta _{d-1})$, and set $h:=\Big \lceil (1/\epsilon )^{\tilde{\eta }^4}\Big \rceil$, $r:=\Big \lceil (1/\epsilon )^{\tilde{\eta }^3}\Big \rceil$, $s:=\Big \lceil (1/\epsilon )^{\tilde{\eta }^2}\Big \rceil$, and $u:=\Big \lceil (1/\epsilon )^{\tilde{\eta }}\Big \rceil$.

As was explained in the end of Sect. 2.6, our main recurrence “launches” with $f(\epsilon ,1,1)=f_d(\epsilon )$, and has only constant depth in the second parameter ρ. Though the restriction threshold σ>0 may decrease in the course of this recurrence in ρ, for the rest of this paper it will be assumed that σ is bounded from below by a positive constant $\sigma _0$ that satisfies the inequality $\sigma _0\le 2^{-\lceil \log _h (1/\epsilon )\rceil }$ for any ϵ<1/100. (The condition is clearly met by $\sigma _0\ge 2^{-cd^{40}/\eta ^4}$, where c>0 denotes some absolute constant which does not depend on η or *d*.)

It can, furthermore, be assumed that $\rho >\epsilon$ for, otherwise, Theorem 3.3 yields the estimate

5 $$\begin{aligned} f(\epsilon ,\rho ,\sigma )\le f(\epsilon ,\epsilon ,\sigma )=O\left( \frac{s^{(d-1)^2}}{\sigma ^{\beta _{d-1}}\epsilon ^{d-1}}+s\cdot f\left( \sigma ^{\beta _{d-1}}s^{1/(d-1)}\cdot \epsilon \right) \right) = \end{aligned}$$

$$ =O\left( \frac{s^{(d-1)^2}}{\epsilon ^{d-1}}+s\cdot f\left( s^{1/(d-1)}\cdot \epsilon \right) \right) . $$

To facilitate our analysis, we also introduce two auxiliary fractional parameters $0<\varepsilon \le 1$ and $0<\delta \le 1$, which too depend on ϵ. The first parameter δ is a very small, yet positive, power of ϵ which is given by

6 $$\begin{aligned} \delta =\delta (\epsilon ):=\frac{1}{r^{d(d+1)+1}}. \end{aligned}$$

The second parameter ε>0 is only marginally smaller than ϵ, and satisfies

7 $$\begin{aligned} \varepsilon :=\frac{\vartheta ^2\sigma _0^{\beta _{d-1}}\delta }{10^6\cdot 2^{\beta _{d-1}}d^2(d+1){(d-1)d\atopwithdelims ()d-1}r}\cdot \epsilon =\Omega \left( \epsilon ^{1+\eta }\right) , \end{aligned}$$

where ϑ>0 is the constant in the Canonical Line-Selection Theorem 3.2.

To bound the quantity $f(\epsilon ,\rho ,\sigma )$ for $\epsilon <\min \{\rho ,\epsilon _0\}$, we fix a finite point set *P* in a general position in $\mathbb {R}^d$, with a subset Π of at most $\rho {P\atopwithdelims ()d}$ simplices within Pd, and construct a small-size net *N* for the induced “edge-constrained” sub-family ${{\mathcal {K}}}={{\mathcal {K}}}(P,\Pi ,\epsilon ,\sigma )$ within ${{\mathcal {K}}}(P,\epsilon )$. The cardinality of *N* will then serve as an upper bound on $f(\epsilon ,\rho ,\sigma )$.

In the rest of this proof, it can be assumed, with no loss of generality, that the ground point set *P* is larger than a certain threshold $n_0(\epsilon )$ which satisfies

8 $$\begin{aligned} n_0(\epsilon )=\max \left\{ 100\cdot s\cdot \lceil 1/\epsilon ^{\alpha _d}\rceil ,\frac{100ds}{\varepsilon } \right\} \ge 100st. \end{aligned}$$

If this is not the case, then our net N=P for ${{\mathcal {K}}}$ consists of $|P|\le n_0(\epsilon )=O\left( 1/\epsilon ^{\alpha _d+\eta }\right) =O\left( 1/\epsilon ^{\alpha _d+\gamma }\right)$ points. In the sequel, it can be explicitly assumed that $|\Pi |\ge \epsilon {n\atopwithdelims ()d}$ or, else, such a family ${{\mathcal {K}}}$ is again pierced by a net whose maximum cardinality $f(\epsilon ,\epsilon ,\sigma )$ has been estimated in (5).

### The Net *N*

**Piercing the** ε**-narrow sets.** Since $|P|>n_0(\epsilon )\ge s$, the point set *P* admits the vertical *s*-partition $\mathcal {V}(P,s)=\{(P_1,\Delta _1),\ldots ,(P_s,\Delta _s)\}$, which has been described in Sect. 3.1. According to Theorem 3.2, this partition $\mathcal {V}(P,s)$ is endowed with the ϑ-canonical set $\mathcal {{L}}(P,s)$ of $O\left( s^{(d-1)^2}\right)$ lines. Lemma 3.1 yields the net $N_{nar}(P,s,\varepsilon )$ whose cardinality satisfies

$$ |N_{nar}(P,\epsilon ,\varepsilon )|\le s\cdot f\left( s^{1/(d-1)}\varepsilon \right) =s \cdot f\left( \epsilon \cdot \frac{s^{1/(d-1)}}{r^{d(d+1)+2}}\right) \ll s\cdot f\left( \epsilon \cdot s^{1/(d-1)-\eta }\right) , $$

and that pierces every convex set *K* in ${{\mathcal {K}}}(P,\epsilon )$ that is ε-narrow with respect to the vertical partition $\mathcal {V}(P,s)$.

#### Definition

We say that a set $K\in {{\mathcal {K}}}(P,\epsilon )$ is δ*-punctured* (resp., δ*-hollow*) if its principal subset $P_K$ are δ-punctured (resp., δ-hollow) according to the definitions in Sect. 2.2.

Thus, every remaining, ε-spread set in the family ${{\mathcal {K}}}={{\mathcal {K}}}(P,\Pi ,\epsilon ,\sigma )$ will be pierced by the combination $N_{>\delta }\cup N_{\le \delta }$ of the following two nets:

(i) The net $N_{>\delta }$ for the family ${{\mathcal {K}}}_{>\delta }={{\mathcal {K}}}_{>\delta }(P,\epsilon )$, which is comprised of all the δ-punctured sets $K\in {{\mathcal {K}}}(P,\epsilon )$ that are ε-spread with respect to $\mathcal {V}(P,s)$.^23^
(ii) The net $N_{\le \delta }$ for the family ${{\mathcal {K}}}_{\le \delta }={{\mathcal {K}}}_{\le \delta }(P,\Pi ,\epsilon ,\sigma )$, which encompasses all the δ-hollow sets *K* in the family ${{\mathcal {K}}}$ that are ε-spread with respect to $\mathcal {V}(P,s)$.

**The net** $N_{>\delta }$. In Sect. 5 we use the 1-dimensional reduction of Sect. 3 in order to establish the following statement.

#### Theorem 4.1

Let d≥3. One can fix a constant $d/2\le \theta _d\le \alpha _d$ so that, with the previous choice of the constant η>0, the parameters $0<\epsilon \le 1$ and $\delta =\delta (\epsilon )$, and the finite point set *P* in $\mathbb {R}^d$, the induced family ${{\mathcal {K}}}_{>\delta }={{\mathcal {K}}}_{>\delta }(P,\epsilon )$ within ${{\mathcal {K}}}(P,\epsilon )$ can be pierced by a net $N_{>\delta }$ whose cardinality satisfies

$$ |N_{>\delta }|\le {t}^{1+\eta }\cdot f\left( \epsilon \cdot {t}^{(1/\alpha _d)-\eta }\right) +{{u}}^{1+\eta }\cdot f\left( {{u}}^{\frac{1}{d-1}-\eta }\cdot \epsilon \right) +O\left( 1/\epsilon ^{\alpha _d+\eta }\right) . $$

Here $t=\Big \lceil (1/\epsilon )^{\theta _d}\Big \rceil$, whereas *s* and *u* denote the previously selected (and small) powers of 1/ϵ which satisfy $s\lll _\eta u\lll _\eta 1/\epsilon$.

As was outlined in the end of Sect. 2, the key to the proof of Theorem 4.1 lies in obtaining an $O^*\left( \epsilon ^{d-\alpha _d}n^{d}\right)$-size subset $\tilde{\Pi }\subset {P\atopwithdelims ()d}$ of (d-1)-simplices that can be plugged into Theorem 3.3 instead of Pd. Since such a sparse substitute $\tilde{\Pi }$ may not exist in absence of further restrictions on the *shape* of the sets in ${{\mathcal {K}}}_{>\delta }$, it will be constructed only for the more favourable category of “hyperplane-like” sets *K* within ${{\mathcal {K}}}_{>\delta }$, which will be called *flat*. To dispose of the remaining sets $K\in {{\mathcal {K}}}_{>\delta }$, which are either “relatively thick” or their principal subsets $P_K$ are not in “sufficiently convex” position, the application of Theorem 3.3 will be preceeded by introducing several auxiliary nets. Both the construction of $N_{>\delta }$ and its analysis will be facilitated by the vertical partition $\mathcal {V}(P,s)$, the canonical line family $\mathcal {{L}}(P,s)$, and secondary simplicial partitions $\mathcal {P}_i=\mathcal {P}(P_i,t)$ which will be constructed for each part $P_i$ in the vertical partition $\mathcal {V}(P,s)$ of *P*.

**The net** $N_{\le \delta }$. Specializing to the sub-family ${{\mathcal {K}}}_{\le \delta }$ *within* ${{\mathcal {K}}}={{\mathcal {K}}}(P,\Pi ,\epsilon ,\sigma )$, in Sect. 6 we obtain a small-size net whose properties are summarized in the following theorem.

#### Theorem 4.2

Let d≥3. With the previous choice of the constants η and $\sigma _0$, of the fractions $0<\epsilon \le 1$, $2\sigma _0\le \sigma \le 1$ and $\delta =1/r^{d(d+1)+1}$, and of the finite point set *P* in $\mathbb {R}^d$, along with $\Pi \subseteq {P\atopwithdelims ()d}$, the induced family ${{\mathcal {K}}}_{\le \delta }$ within ${{\mathcal {K}}}={{\mathcal {K}}}(P,\Pi ,\epsilon ,\sigma )$ can be pierced by a net $N_{\le \delta }$ whose cardinality satisfies

$$ |N_{\le \delta }|\le f(\epsilon ,\rho /h,\sigma /2)+f\left( \epsilon \log r\right) + $$

$$ +O\left( r^{\alpha _d+\eta }\cdot f\left( \epsilon \cdot r^{1-\eta }\right) +\sum _{i=1}^l 2^iw^{\alpha _d+\eta }\cdot f\left( \epsilon \cdot 2^i w\right) +\frac{1}{\epsilon ^{\alpha _d+\eta }}\right) , $$

Here *r* and *s* denote the previously selected and small powers of 1/ϵ which satisfy $r\lll _\eta s\lll _\eta 1/\epsilon$, $w=r^{\sqrt{d^2-2d}-d+2}$, and *l* is a positive integer that satisfies l=O(logr).

The treatment of the family ${{\mathcal {K}}}_{\le \delta }={{\mathcal {K}}}_{\le \delta }(P,\Pi ,\epsilon ,\sigma )$ will overly resemble that of ${{\mathcal {K}}}_{>\delta }$, with the following key differences: (i) instead of using the secondary simplicial partitions $\mathcal {P}_i=\mathcal {P}(P_i,t)$, the underlying point set *P* will be subdivided by the means of a certain $\Theta \left( \frac{\log r}{r}\right)$-cutting $\mathcal {D}$ of the hyperplane family ${\mathcal {H}}={\mathcal {H}}(\Pi )$, and (ii) instead of using Theorem 3.3, in the the last step we will resort to a more elementary recurrence in ϵ>0 and the ground set *P*.

### Wrap-Up

The complete net *N* for the family ${{\mathcal {K}}}={{\mathcal {K}}}(P,\Pi ,\epsilon ,\sigma )$ is, therefore, given by the combination

9 $$\begin{aligned} N:=N_{nar}(P,s,\varepsilon )\cup N_{>\delta }\cup N_{\le \delta }, \end{aligned}$$

whose cardinality, in view of Theorems 4.1 and 4.2, satisfies

10 $$\begin{aligned} |N|\le f(\epsilon ,\rho /h,\sigma /2)+F(\epsilon ), \end{aligned}$$

with

$$\begin{aligned} F(\epsilon )&=O\left( f(\epsilon \cdot \log r)+{s}\cdot f\left( \epsilon \cdot {s}^{\frac{1}{d-1}-\eta }\right) +{t}^{1+\eta }\cdot f\left( \epsilon \cdot {t}^{(1/\alpha _d)-\eta }\right) \right. +\\&\quad \left. +\,{{u}}^{1+\eta }\cdot f\left( {{u}}^{\frac{1}{d-1}-\eta }\cdot \epsilon \right) + r^{\alpha _d+\eta }\cdot f\left( \epsilon \cdot r^{1-\eta }\right) \right. +\\&\quad \left. + \sum _{i=1}^l 2^iw^{\alpha _d+\eta }\cdot f\left( \epsilon \cdot 2^i w\right) +1/\epsilon ^{\alpha _d+\eta }\right) . \end{aligned}$$

Here $t=\Big \lceil 1/\epsilon ^{\theta _d}\Big \rceil$ is an integer that depends only on ϵ, and satisfies $\Big \lceil 1/\epsilon ^{d/2}\Big \rceil \le t\le \Big \lceil 1/\epsilon ^{\alpha _d}\Big \rceil$, $w=r^{\sqrt{d^2-2d}-d+2}$, and *l* is a positive integer that satisfies l=O(logr). As was previously mentioned, the same upper bound must also hold for the quantity $f(\epsilon ,\rho ,\sigma )$, which thus satisfies

11 $$\begin{aligned} f(\epsilon ,\rho ,\sigma )\le f(\epsilon ,\rho /h,\sigma /2)+F(\epsilon ). \end{aligned}$$

To obtain a complete recurrence formula for f(ϵ), we begin with $$f(\epsilon )=f(\epsilon ,1,1)$$ and invoke the bound (11) $$j_0=j_0(\epsilon ,\eta ):=\lceil \log _h(1/\epsilon )\rceil =O\left( d^{40}/\eta ^4\right) $$ times, each time only to the first term of the general form $$f(\epsilon ,\rho ',\sigma ')$$, while leaving all the remaining recursive terms $f(\epsilon^{\prime })$ intact. Since each iteration reduces the density ρ by a factor of *h*, and σ by a factor of 2, this yields

12 $$\begin{aligned} f(\epsilon )\le f\left( \epsilon ,1/h^{j_0},\sigma _0\right) +\sum _{i=0}^{j_0-1}F\left( \epsilon \right) , \end{aligned}$$

where $\sigma _0>0$ denotes the aforementioned constant $2^{-cd^{12}/\eta ^4}$ which satisfies $\sigma _0\le 1/2^{j_0}$. Applying Theorem 3.3 to the first term $f\left( \epsilon ,1/h^{j_0},\sigma _0\right)$ of (12), with $\rho \le 1/h^{j_0}\le \epsilon$, yields

13 $$\begin{aligned}&f(\epsilon )\le j_0\cdot F(\epsilon )+O\left( \frac{s^{(d-1)^2}}{\epsilon ^{d-1}}+s\cdot f\left( s^{1/(d-1)}\cdot \epsilon \right) \right) =\nonumber \\&=O\left( f(\epsilon \cdot \log r)+{s}\cdot f\left( \epsilon \cdot {s}^{\frac{1}{d-1}-\eta }\right) +{t}^{1+\eta }\cdot f\left( \epsilon \cdot {t}^{(1/\alpha _d)-\eta }\right) \right. +\nonumber \\&\quad \left. +\, {u}^{1+\eta }\cdot f\left( {{u}}^{\frac{1}{d-1}-\eta }\cdot \epsilon \right) + r^{\alpha _d+\eta }\cdot f\left( \epsilon \cdot r^{1-\eta }\right) \right. +\nonumber \\&\quad \left. + \sum _{i=1}^l 2^iw^{\alpha _d+\eta }\cdot f\left( \epsilon \cdot 2^i w\right) +1/\epsilon ^{\alpha _d+\eta }\right) , \end{aligned}$$

with implicit constants of proportionality that depend only on $\eta =\eta (\gamma )>0$, and the dimension d≥3.

Recall that the recurrence in ϵ>0 bottoms out as soon as ϵ by-passes a certain upper threshold $\epsilon _0>0$. Since η is much smaller than γ, fixing a sufficiently small constant threshold $\epsilon _0=\epsilon _0(d,\gamma )$, and following the routine induction argument which applies to all recurrences of this type (see, e.g., [35, 53, 57] and [58, Section 7.3.2]), yields

14 $$\begin{aligned} f(\epsilon )\le \frac{a_0}{\epsilon ^{\alpha _d+\gamma }}, \end{aligned}$$

where $a_0\ge f(\epsilon _0)$ is a suitably large constant which too depends on *d* and γ>0. For the sake of completeness, the key details of this standard induction are spelled out in Appendix C. This concludes the proof of Theorem 1.1□

## Piercing the δ-Punctured Sets – Proof of Theorem 4.1

We stick with the overall setup of Sect. 4, which includes $0<\epsilon \le 1$, the auxiliary integer parameters *r*, *s*, *u*, which have been defined in Sect. 4 as very small yet positive powers of 1/ϵ which satisfy $r\lll _\eta s\lll _\eta u\lll _\eta 1/\epsilon$, and the *n*-point set *P* within $\mathbb {R}^d$, which satisfies $n=|P|\ge n_0(\epsilon )\ge 100s\lceil 1/\epsilon ^{\alpha _d}\rceil \ge 100st$. Moreover, the proof of Theorem 4.1 will be facilitated by the vertical partition $\mathcal {V}(P,s)$, and the ϑ-canonical family $\mathcal {{L}}(P,s)$ of $O\left( s^{(d-1)^2}\right)$ vertical lines, whose choice is too described in Sect. 4.

To establish Theorem 4.1, we fix the exponent $d/2\le \theta _d\le \alpha _d$, along with the integer parameter $t=\lceil (1/\epsilon )^{\theta _d}\rceil$, which will be selected in the end of this section, and describe a small-size net $N_{>\delta }$ for the family ${{\mathcal {K}}}_{>\delta }$, which is comprised of all such δ-punctured sets $K\in {{\mathcal {K}}}(P,\epsilon )$ that are ε-spread with respect to $\mathcal {V}(P,s)=\{(P_1,\Delta _1),\ldots ,(P_s,\Delta _s)\}$. The construction of $N_{>\delta }$ begins with an empty set, and proceeds through four steps.

In the preparatory “0-step”, we establish the following crucial property of the δ-punctured sets $K\in {{\mathcal {K}}}_{>\delta }$: each of them can be assigned an ordered (d+1)-tuple $\Psi =(P_{j_0},\ldots ,P_{j_{d}})$ of subsets within $\mathcal {V}(P,s)$, so that (i) $P_{j_0}$ is surrounded by the remaining *d* subsets $P_{j_i}$, with $1\le i\le d$, and (ii) each subset $P_{j_i}$ in Ψ contains $\Omega (\delta \epsilon n/s)\gg \epsilon ^{1+\eta }n$ points of $P_K$. In what follows, every convex set $K\in {{\mathcal {K}}}_{>\delta }$ will be assigned a unique such (d+1)-tuple $\Psi (K)=(P_{j_0},\ldots ,P_{j_{d}})$, which will be referred to as the *principal* (d+1)*-tuple of* *K*.

In each of the subsequent three steps $1\le i\le 3$, we will define, and immediately add to $N_{>\delta }$, a new auxiliary net $N_{>\delta ,i}$. Once a set $K\in {{\mathcal {K}}}_{>\delta }$ is pierced by $N_{>\delta ,i}$, it is immediately removed from further consideration, whereas the surviving sets of ${{\mathcal {K}}}_{>\delta }$ are passed on to the subsequent steps. Upon the completion of the second step, every remaining set $K\in {{\mathcal {K}}}_{>\delta }$ will be sufficiently flat in the sense that was outlined in Sect. 2.7. With these preparations, in the third step we will at last construct a sparse subset $\tilde{\Pi }\subseteq {P\atopwithdelims ()d}$ that is “sufficiently dense” over every remaining $\lceil \epsilon n\rceil$-subset $P_K$ that is determined by some $K\in {{\mathcal {K}}}_{>\delta }$, so the third net $N_{>\delta ,3}$ will be obtained through a straightforward application of Theorem 3.3.

### Step 0: Fixing a Principal (d+1)-Tuple

#### Lemma 5.1

**Fig. 15:**
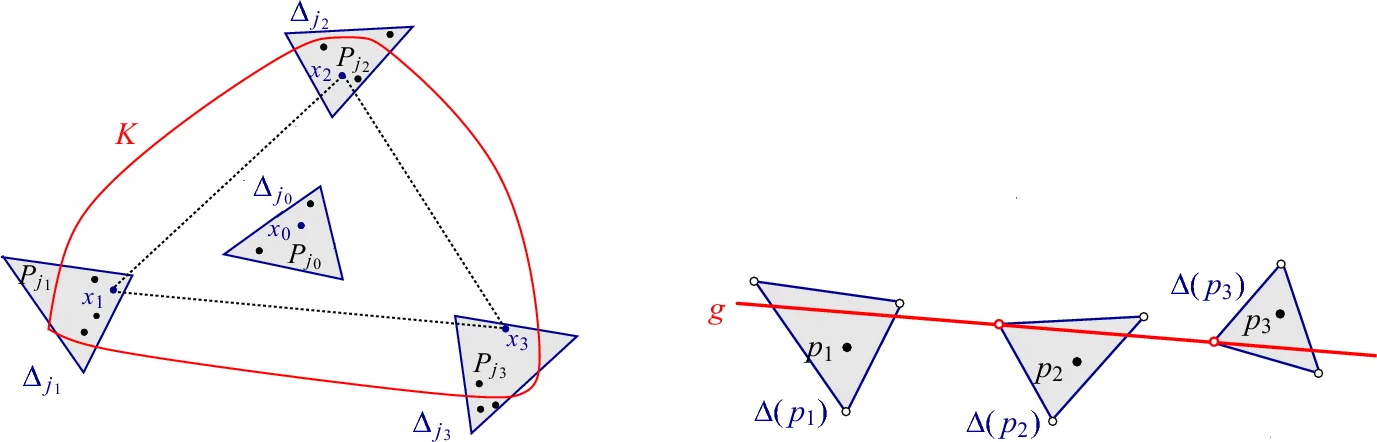
Left: Lemma 5.1 in dimension d=3. The 4-tuple $\Psi =\left( P_{j_0},P_{j_1},P_{j_2},P_{j_3}\right)$ is depicted in the vertical projection. We have that $|P_K\cap P_{j_i}|\ge \frac{\delta |P_K|}{(4d+4)s}$. The cell $\Delta _{j_0}$ is surrounded by the cells $\Delta _{j_1},\Delta _{j_2}$, and $\Delta _{j_3}$: for each selection $x_i\in \Delta _{j_i}$ we have that $x_0\in {{\texttt{conv}}}(x_1,x_2,x_3)$. Right: Proof of Lemma 5.2 in $\mathbb {R}^3$. The depicted bad triple $A=\{p_1,p_2,p_3\}$ within $P_K$ is labeled with (*B*, *g*), where $B=\{p_2,p_3\}$, and the 1-flat (i.e., line) *g* passes through a pair of vertices of $\Delta (p_1)$ and $\Delta (p_2)$, and crosses $\Delta (p_3)$

Let $K\in {{\mathcal {K}}}_{>\delta }$. Then the partition $\mathcal {V}(P,s)=\{(P_1,\Delta _1),\ldots ,(P_s,\Delta _s)\}$ contains an ordered (d+1)-tuple $\Psi =\left( P_{j_0},\ldots ,P_{j_{d}}\right)$ with the following properties (see Fig. 15 (left)):

1. The ambient cell $\Delta _{j_{0}}$ of $P_{j_{0}}$ is surrounded by the respective ambient cells $\Delta _{j_i}$ of the remaining *d* sets $P_{j_i}$, for $1\le i\le d$.
2. For all $0\le i\le d$, we have that
  $$ |P_K\cap P_{j_i}|\ge \frac{\delta |P_K|}{(4d+4)s} $$

#### Proof

Let us begin with introducing a few handy notations. Recall that, according to our definition in Sect. 2.2, a collection of (d-1)-dimensional simplicial cells $\{\Delta _{i_1},\ldots ,\Delta _{i_k}\}$ in $\mathcal {V}(P,s)$, with k≥d and $1\le j_1\ne j_2\ne \ldots \ne j_k\le {s}$, is *separated* if no *d* of its members can be intersected by a single (d-2)-flat in $\mathbb {R}^{d-1}$.

We then say, for k≥d, that a *k*-size subset of points $\{p_1,\ldots ,p_k\}\in {P_K\atopwithdelims ()k}$ is *good* if their ambient cells $\Delta (p_i)$ in the vertical partition $\mathcal {V}(P,s)$ are all distinct, and form a separated collection $\{\Delta (p_1),\ldots ,\Delta (p_k)\}$. In any other case, the *k*-size subset $\{p_1,\ldots ,p_k\}$ will be called *bad*.

For the rest of this proof, it can be assumed that $\lceil \epsilon n\rceil \ge 2d+2$ for, otherwise, there are no ε-spread convex sets in ${{\mathcal {K}}}_{>\delta }$, and there is nothing to prove.

Our proof of Lemma 5.1 relies on the following property.

#### Lemma 5.2

There exist at most $(\delta /(4(d+1)){|P_K|\atopwithdelims ()d}$ bad *d*-size subsets in ${P_K\atopwithdelims ()d}$. Hence, there exist at most $(\delta /4){|P_K|\atopwithdelims ()d+1}$ bad (d+1)-subsets in ${P_K\atopwithdelims ()d+1}$.

#### Proof Lemma 5.2

Clearly, the second property follows from the first one, as any bad (d+1)-tuple in ${P_K\atopwithdelims ()d+1}$ of points can be “charged” to a bad *d*-tuple in ${P_K\atopwithdelims ()d}$, whereas any bad *d*-tuple is part of at most $|P_K|-d$ bad (d+1)-tuples, for a total of at most

$$ \left( |P_K|-d\right) \left( \frac{\delta }{4(d+1)}\right) \frac{|P_K|\cdot (|P_K|-1)\ldots (|P_K|-d+1)}{d!} $$

such bad (d+1)-tuples in ${P_K\atopwithdelims ()d+1}$.

Assume for a contradiction that there exist more than $\frac{\delta }{4(d+1)}{P_K\atopwithdelims ()d}$ bad subsets in ${P_K\atopwithdelims ()d}$. Let $\mathcal {B}_K\subseteq {P_K\atopwithdelims ()d}$ denote the collection of all such bad *d*-size subsets in ${P_K\atopwithdelims ()d}$. Fix any $A\in \mathcal {B}_K$. Then there must be a (d-2)-flat *g* within $\mathbb {R}^{d-1}$ that intersects every simplicial cell in the family $\Gamma _A:=\{\Delta (p_1),\ldots ,\Delta (p_d)\}$, whose cardinality may be smaller than *d*. In particular, if $|\Gamma _A|\le d-1$, so that $\Delta (p_j)=\Delta (p_{j'})$ for some $1\le j\ne j'\le d$, then *every* (d-2)-flat *g* through $A^\perp \setminus \{p^\perp _i\}$ meets every simplex in Σ.

Applying Lemma 2.3 to this family $\Gamma _A$, which is comprised of at least one (d-1)-dimensional simplicial cell in $\mathbb {R}^{d-1}$, yields a (d-1)-subset $B\in {A\atopwithdelims ()d-1}$, and a (d-2)-flat transversal *g* to $\Gamma _A$ that passes through some d-1 boundary vertices of the cells in the family $\Gamma _B=\{\Delta (p)\mid p\in B\}$. See Fig. 15 (right). We thus label *A* with such a pair (*B*, *g*), and repeat the above argument to all the (at least) $\frac{\delta }{4(d+1)}{\lceil \epsilon n\rceil \atopwithdelims ()d}$ such bad *d*-size sets $A\in \mathcal {B}_K$. Since any (d-1)-set $B\in {P_K\atopwithdelims ()d-1}$ participates in at most (d-1)dd-1 of the labels (*B*, *g*), by the pigeonhole principle, and the prior choice (6) of the threshold ε, there must be such a label (*B*, *g*) that is shared by at least

$$ \frac{\delta {\lceil \epsilon n\rceil \atopwithdelims ()d}}{4(d+1){(d-1)d\atopwithdelims ()d-1}{\lceil \epsilon n\rceil \atopwithdelims ()d-1}} \ge \varepsilon n $$

of the degenerate *d*-size sets $A\in \mathcal {D}_K$. The desired contradiction follows because each of these degenerate *d*-sets *A* contributes a distinct point $p_A\in A\setminus B$ whose ambient cell $\Delta \left( p_A\right)$ is intersected by *H*, for a total of at least εn of such points. Hence, *K* must be ε-narrow in $\mathcal {V}(P,s)$ and, thereby, have previously been removed from consideration. □

*Back to the proof of Lemma* 5.1. Since *K* is δ-punctured, the family ${P_K\atopwithdelims ()d+1}$ must contain a sub-family $\mathcal {E}_K$ of at least $\delta {|P_K|\atopwithdelims ()d+1}$ punctured (d+1)-subsets *A*. By the previous Lemma 5.2, at most $(\delta /4){|P_K|\atopwithdelims ()d+1}$ of these (d+1)-subsets in $\mathcal {E}_K$ can be bad.

Let

$$ \mathcal {V}':=\left\{ P_i\mid 1\le i\le {s}, |P_i\cap P_K|\le \frac{\delta }{4{s}(d+1)} |P_K|\right\} . $$

Since the sets of $\mathcal {V}'$ encompass at total of at most $\delta |P_K|/(4d+4)$ points of $P_K$, at most $(\delta /4){|P_K|\atopwithdelims ()d+1}$ of the (d+1)-size sets $A\in \mathcal {E}_K$ can include a point of $P_K\cap \left( \bigcup \mathcal {V}'\right)$.

In particular, there must remain at least one good (d+1)-set $A=\{p_0,\ldots ,p_{d}\}$ in $\mathcal {E}_K$, so that $A\subset \bigcup \left( \mathcal {V}(P,s)\setminus \mathcal {V}'\right)$.^24^ Assume with no loss of generality that $p_{0}^\perp \in {{\texttt{conv}}}\left( p_1^\perp ,\ldots ,p_d^\perp \right)$. Since the simplices $\Delta (p_0),\ldots ,\Delta (p_d)$ are separated in $\mathbb {R}^{d-1}$, and $\Delta (p_0)$ clearly intersects ${{\texttt{conv}}}\left( \bigcup _{i=1}^d \Delta (p_i)\right)$ at the point $p_0$, it follows via Corollary 2.4 that $\Delta \left( p_{0}\right)$ must be surrounded by the cells $\Delta \left( p_{i}\right)$, for $1\le i\le d$. Furthermore, as *A* is contained in $\bigcup \mathcal {P}(s)\setminus \mathcal {V}'$, the respective d+1 subsets $P_{j_i}\ni p_i$ of $\mathcal {V}(P,s)$ are distinct and satisfy $|P_{j_i}|\ge \delta |P_K|/((4d+4){s})$ for all $0\le i\le d$. Hence, the (d+1)-tuple $(P_{j_0},\ldots ,P_{j_{d}})$ meets both criteria (P1) and (P2), which completes the proof of Lemma 5.1. □

#### Definition

We assign to each $K\in {{\mathcal {K}}}_{>\delta }$ a *unique* ordered (d+1)-tuple $\Psi (K)=(P_{j_0},\ldots ,P_{j_d})$ of subsets in $\mathcal {V}(P,s)$ that meet the criteria (P1) and (P2), and refer to Ψ(K) as the *principal* (d+1)*-tuple* of *K* in $\mathcal {V}(P,s)$.

### Step 1

To facilitate the subsequent divide-and-conquer treatment of the family ${{\mathcal {K}}}_{>\delta }$, let us first refine the underlying vertical *s*-partition $\mathcal {V}(P,s)=\{(P_1,\Delta _1),\ldots ,(P_s,\Delta _s)\}$ of *P* by the means of two secondary (and mutually independent) sub-partitions $\mathcal {P}(P,s,t)$ and $\mathcal {W}(P,s,u)$. Note that, in the view of the previous assumption that $n\ge n_0(\epsilon )$, each non-empty part $P_i$ in $\mathcal {V}(P,s)$ satisfies $|P_i|\ge \lceil |P|/s\rceil \ge 10t\ggg _\eta u$.

**Fig. 16:**
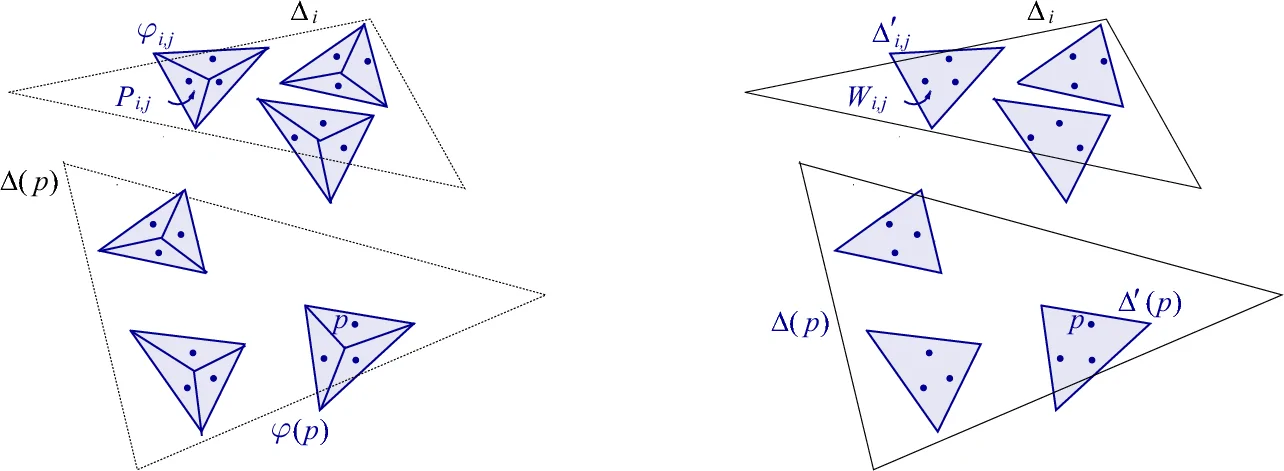
Left: The secondary partition $\mathcal {P}(P,s,t)$ of *P* in $\mathbb {R}^3$ – view from above. Each subset $P_i$ in $\mathcal {V}(P,s)$ is subdivided into pairwise disjoint parts $P_{i,j}$ of size at most $2\lceil |P_i|/t\rceil$, each of them enclosed by a cell $\varphi _{i,j}$ in $\mathbb {R}^3$. Right: The secondary vertical partition $\mathcal {W}(P,s,u)$, which is obtained by constructing the vertical partition $\mathcal {V}(P_i,u)$ for each part $P_i$

**The secondary simplicial partition** $\mathcal {P}(P,s,t)$. We invoke the simplicial partition of Theorem 2.6 for each part $P_i\in \mathcal {V}(P,s)$ so as to obtain the secondary *d*-dimensional simplicial *t*-partition $\mathcal {P}\left( P_i,t\right) =\{(P_{i,j},\varphi _{i,j})\mid 1\le j\le {t}\}$. See Fig. 16 (left). We then denote

$$ \mathcal {P}(P,s,t):=\bigcup _{i=1}^s \mathcal {P}\left( P_i,t\right) =\{\left( P_{i,j},\varphi _{i,j}\right) \mid 1\le i\le s,1\le j\le t\}. $$

For each point $p\in P_i$, its *ambient cell* φ(p) *in* $\mathcal {V}(P,s,t)$ is defined as the ambient cell of *p* in the secondary simplicial partition $\mathcal {P}(P_i,s)$ of $P_i$.

**The secondary vertical partition** $\mathcal {W}(P,s,u)$. We apply the vertical partition of Sect. 3.1 to each part $P_i\in \mathcal {V}(P,s)$, to obtain an auxiliary vertical *u*-partition $\mathcal {V}(P_i,u)=\{(W_{i,k},\Delta '_{i,k})\mid 1\le k\le u\}$ as detailed in Sect. 3.2. See Fig. 16 (right). We then set

$$ \mathcal {W}(P,s,u):=\bigcup _{i=1}^s \mathcal {V}(P_i,u)=\{\left( W_{i,k},\Delta '_{i,k}\right) \mid 1\le i\le s,1\le k\le u\}. $$

For each point $p\in P_i$, the *ambient cell* $\Delta '(p)\subset \mathbb {R}^{d-1}$ *in* $\mathcal {W}(P,s,u)$ is accordingly defined as the ambient cell of *p* in the secondary vertical partition $\mathcal {V}(P_i,s)$ of $P_i$.

**The net** $N_{>\delta ,1}$. Similar to the recursive net $N_{nar}(P,s,\varepsilon )$ in Sect. 3.2, the net $N_{>\delta ,1}$ is a combination of several recursively defined nets, which are constructed over ground sets $P_{i,j}$ (resp., $W_{i,k}$) and are cut out by the subsets in the secondary partitions $\mathcal {P}(P,s,t)$ and $\mathcal {W}(P,s,u)$. Their inclusion in $N_{>\delta }$ will guarantee, for all of the remaining sets $K\in {{\mathcal {K}}}_{>\delta }$, that the points of their principal subsets $P_K$ are sufficiently well-distributed among the parts of $\mathcal {P}(P,s,t)$ and $\mathcal {W}(P,s,u)$.

To this end, let

15 $$\begin{aligned} \epsilon _1:=\cdot \frac{\delta \epsilon }{st^{1-1/\alpha _d}}. \end{aligned}$$

The net $N_{>\delta ,1}$ is comprised of the following ingredients:

1. For each $1\le i\le s$, and each $1\le j\le t$, we construct the net $N\left( P_{i,j},\epsilon _1\cdot st\right)$, which pierces the recursive sub-family ${{\mathcal {K}}}\left( P_{i,j},\epsilon _1\cdot st\right)$. Notice that the overall contribution of these nets $N\left( P_{i,j},\epsilon _1\right)$ to $|N_{>\delta ,1}|$ amounts to
  $$ \left| \bigcup _{1\le i\le s,1\le j\le t}N\left( P_{i,j},\epsilon _1\cdot st\right) \right| \le st\cdot f\left( \epsilon _1\cdot st\right) =st\cdot f\left( \epsilon \cdot t^{1/\alpha _d}\right) . $$
2. For $1\le i\le s$ we construct the net $N_{nar}(P_i,u,\varepsilon )$, as described in Lemma 3.1, which pierces all the convex sets $K\in {{\mathcal {K}}}_{>\delta }$ that are ε-narrow in the secondary vertical subdivision $\mathcal {V}(P_i,u)$ of the set $P_i\in \mathcal {V}(P,s)$. Clearly, the overall contribution of these recursive nets amounts to at most
  $$ \left| \bigcup _{i=1}^s N_{nar}(P_i,u,\varepsilon )\right| \le su\cdot f\left( \varepsilon \cdot u^{1/(d-1)}\right) . $$

**Analysis.** The properties of $N_{>\delta ,1}$ are summarized in the following statement (using that $s\lll _\eta u\lll _\eta 1/\epsilon =O(t)$).

#### Lemma 5.3

1. The cardinality of the net
  $$ N_{>\delta ,1}:=\left( \bigcup _{1\le i\le s,1\le j\le t}N\left( P_{i,j},\epsilon _1\cdot st\right) \right) \cup \left( \bigcup _{i=1}^s N_{nar}(P_i,u,\varepsilon )\right) $$
  satisfies
  $$ N_{>\delta ,1}\le st\cdot f\left( \epsilon \cdot t^{1/\alpha _d}\right) +su\cdot f\left( \varepsilon \cdot u^{1/(d-1)}\right) = $$
  $$ =O\left( t^{1+\eta }\cdot f\left( \epsilon \cdot t^{1/\alpha _d}\right) +u^{1+\eta }\cdot f\left( \varepsilon \cdot u^{1/(d-1)}\right) \right) . $$
2. Upon adding the points of $N_{>\delta ,1}$ to our net $N_{>\delta }$ and, thereby, removing all the convex sets that are pierced so far by it, it can be assumed that every remaining convex set $K\in {{\mathcal {K}}}_{>\delta }$ meets the following criteria
  - *K* is ε-spread in each sub-partition $\mathcal {V}(P_i,u)$, and
  - we have that $|P_K\cap P_{i,j}|\le \epsilon _1 n$ for all $1\le i\le s$ and $1\le j\le t$.

### Step 2

The second net $N_{>\delta ,2}$ that we construct for the δ-punctured sets $K\in {{\mathcal {K}}}_{>\delta }$, is a combination of (i) the strong net of Lemma A.1 with respect to convex “(2*d*)-hedra” (i.e., convex polytopes with at most 2*d* vertices), and (ii) a small number of 1-dimensional nets, whose carefully defined ground sets are contained in certain canonical vertical lines. Together with $N_{>\delta ,1}$, its inclusion in $N_{>\delta }$ will at last guarantee that all of the remaining sets $K\in {{\mathcal {K}}}_{>\delta }$, that are missed by $N_{>\delta ,1}\cup N_{>\delta ,2}$, are sufficiently flat with respect to our secondary simplicial partition $\mathcal {P}(P,s,t)$, and thus can be dispatched using the strategy outlined in the end of Sect. 2.

The construction of $N_{>\delta ,2}$ is facilitated by additional geometric constructs, which rely on the secondary sub-divisions $\mathcal {P}(P,s,t)$ and $\mathcal {V}(P,s,u)$ introduced in Step 1.

**The secondary canonical families** $\mathcal {{L}}_i=\mathcal {{L}}(P_i,u)$. For each $1\le i\le s$ we obtain the ϑ-canonical collection $\mathcal {{L}}_i:=\mathcal {{L}}(P_i,u)$ which exists, according to Theorem 3.2, for the secondary vertical partition $\mathcal {V}(P_i,u)$ of $P_i$ (as part of $\mathcal {W}(P,s,u)$). Altogether, this amounts to a total of

$$ \left| \bigcup _{i=1}^s\mathcal {{L}}_i\right| =O\left( su^{(d-1)^2}\right) $$

vertical lines.

**Clipped cells.** For each p∈P we use $\varphi^{\prime }\left(p\right)$ to denote the restriction of its ambient cell φ(p) in $$\mathcal {P}(P,s,t)$$ to the vertical prism above $$\Delta '(p)\cap \Delta (p)$$. (Here Δ(p) denotes the ambient cell of *p* in the primary vertical partition $$\mathcal {V}(P,s)$$, and $\Delta^{\prime }\left(p\right)$ denotes the ambient cell of *p* in the secondary vertical partition $$\mathcal {W}(P,s,u)$$ of Step 1.) In what follows, we refer to $\varphi^{\prime }\left(p\right)$ as the *clipped cell around* *p*.

Notice that, for each $$1\le i\le s$$, and each $$p\in P_i$$, the clipped cell $\varphi^{\prime }\left(p\right)$ is a convex polytope with at most 3d+1 facets which encloses the point set $$P_{i,j}\cap W_{i,k}$$, where $$P_{i,j}$$ (resp., $$W_{i,k}$$) is the part of the secondary partition $$\mathcal {P}(P_i,t)$$ (resp., $$\mathcal {V}(P_i,u)$$) that contains *p*.

#### Definition

In what follows, we use $V^{\prime }\left(p\right)$ to denote the vertex set of $\varphi^{\prime }\left(p\right)$, and we use $V^{\prime }$ to denote the set $$\bigcup _{p\in P}V'(p)$$ of all the vertices of the clipped cells.^25^ Since $$s\lll _\eta u\lll _\eta t$$, we have that $$|V'|=O(stu)=O\left( t^{1+\eta /{10d^2}}\right) $$.

**The net** $N_{>\delta ,2}$. Denote

16 $$\begin{aligned} \epsilon _2:=\frac{c_{ sp}}{100(4d+4)}\cdot \frac{\delta \epsilon }{st^{1-1/d}}, \end{aligned}$$

where $c_{ sp}>0$ is the constant in Matoušek’s Theorem 2.6.

Let $0<c_2\le 1/100$ be a sufficiently small constant that will be determined in the sequel. Our second net $N_{>\delta ,2}$ for ${{\mathcal {K}}}_{>\delta }$ is comprised of the following non-recursive ingredients:

**Fig. 17:**
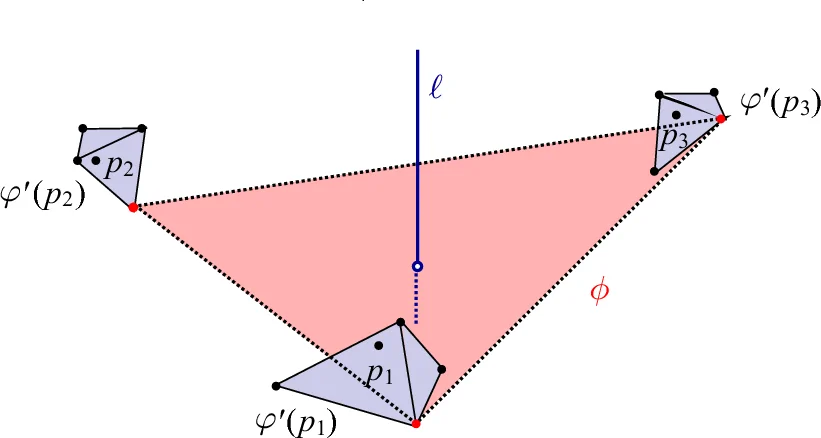
Step 2 in the definition of $N_{>\delta }$ in $\mathbb {R}^3$ – building the ground sets $X'_{\ell }$ for the 1-dimensional nets $N'_\ell$. Depicted are a secondary canonical line $\ell \in \mathcal {{L}}_i$, the clipped cells $\varphi '(p_i)$ around some three points $p_1,p_2,p_3\in P$, and a simplex ϕ spanned by some 3 vertices of $\varphi '(p_1),\varphi '(p_2),\varphi '(p_3)$. Then the ℓ-intercept $\phi \cap \ell$ is added to $X'_\ell$

1. For each $$1\le i\le s$$, and each line $$\ell \in \mathcal {{L}}_i$$, we construct the set $$X'_\ell =\{\phi \cap \ell \mid \phi \in {V'\atopwithdelims ()d}\}$$ of all the ℓ-intercepts of the (d-1)-dimensional simplices $$\phi \in {V'\atopwithdelims ()d}$$, which are spanned by the boundary vertices of the clipped cells $\varphi^{\prime }\left(p\right)$ of the points p∈P. See Fig. 17.
  We then add to $N_{>\delta ,2}$ the net $N'_\ell$ which includes every $\Big \lceil c_2t^{d-d/\alpha _d}/u^{(d-1)^2}\Big \rceil$-th point of $X'_\ell$.
2. Lastly, we add to $N_{>\delta ,2}$ the strong $(c_2\epsilon _2)$-net $N_{strong}\left( P,c_2\epsilon _2\right)$ of Lemma A.1 that pierces every convex polytope with at most 2*d* vertices that is $(c_2\epsilon _2)$-heavy with respect to *P*. Note that $|N_{strong}\left( P,c_2\epsilon _2\right) |=O\left( \left( 1/\epsilon _2\right) \log \left( 1/\epsilon _2\right) \right)$.

#### Remark

Though the choice of the thresholds $\epsilon _2$ and $c_2t^{d-d/\alpha _d}/u^{(d-1)^2}$ may seem arbitrary at this stage, it will become clearer in the subsequent analysis of Sects. 5.4 and 5.5. For the time being, it is instructive to point out that every convex $K\in {{\mathcal {K}}}_{>\delta }$, that has been pierced by $N_{>\delta ,2}$, must fall into one of the following categories:

(i) its principal set $P_K$ is not in “sufficiently convex” position, or
(ii) the set *K* is “fat” with respect to $\mathcal {P}(P,s,t)$,^26^ so its overall shape is far from a hyperplane.

**The analysis.** The cardinality of $N_{>\delta ,2}$ is bounded by the following statement.

#### Lemma 5.4

The net

$$ N_{>\delta ,2}:=\left( \bigcup _{1\le i\le s}\bigcup _{\ell \in \mathcal {{L}}_i} N'_\ell \right) \cup N_{strong}(P,c_2\epsilon _2) $$

satisfies

$$ |N_{>\delta ,2}|\!=\!O\left( s^{d+1}u^{(d-1)^2\!+\!d}t^{d/\alpha _d}\!+\!\frac{s t^{1-1/d}}{\delta \epsilon }\log \frac{1}{\epsilon }\right) \!=\! O\left( t^{d/\alpha _d\!+\!\eta /(10d)}\!+\!\frac{t^{1-1/d}}{\epsilon ^{1+\eta }}\right) . $$

#### Proof

By the previous choice of $\delta =1/r^{d(d+1)}$, and of $r\lll _\eta s\lll _\eta u \lll _\eta 1/\epsilon \ll t$, it follows that the overall cardinality of the nets $N'_\ell$ satisfies

$$\begin{aligned}  &   \left| \bigcup _{1\le i\le s}\bigcup _{\ell \in \mathcal {{L}}_i} N'_\ell \right| =O\left( \left( \sum _{i=1}^s|\mathcal {{L}}_i|\right) \cdot \frac{u^{(d-1)^2}\left| V'\right| ^d}{t^{d-d/\alpha _d}}\right) = \\    &   =O\left( \frac{s\cdot u^{2(d-1)^2}\cdot {sut\atopwithdelims ()d}}{t^{d-d/\alpha _d}}\right) =O\left( t^{d/\alpha _d+\eta /(10d)}\right) , \end{aligned}$$

while

$$ |N_{strong}(P,c_2\epsilon _2)|=O\left( \frac{1}{\epsilon _2}\log \frac{1}{\epsilon _2}\right) =O\left( \frac{s t^{1-1/d}}{\delta \epsilon }\log \frac{1}{\epsilon }\right) =O\left( \frac{t^{1-1/d}}{\epsilon ^{1+\eta }}\right) . $$

□

We add $N_{>\delta ,2}$ to $N_{>\delta }$ and, thereby, remove from ${{\mathcal {K}}}_{>\delta }$ every convex set that is hit by $N_{>\delta ,2}$.

### Step 3

We are at last ready to describe the third and final net $N_{>\delta ,3}$, which uses Theorem 3.3 to pierce all the remaining δ-punctured ε-spread sets $K\in {{\mathcal {K}}}_{>\delta }$, that have been missed by the combination $N_{>\delta ,1}\cup N_{>\delta ,2}$ of the previous two nets. To this end, we will argue that, pending a suitable choice of the constant $c_2>0$ in the previous Step 2, every remaining set $K\in {{\mathcal {K}}}_{>\delta }$ is sufficiently flat with respect to the secondary partition $\mathcal {P}(P,s,t)$ of *P*, in the sense that will be made formal shortly.

#### Definition

Let $K\in {{\mathcal {K}}}_{>\delta }$ be a convex set with the principal (d+1)-tuple $\Psi (K)=(P_{i_0},\ldots ,P_{i_d})$, where $P_{i_j}\in \mathcal {P}(P,s)$ for all $0\le j\le d$. For the sake of brevity, we relabel the elements of Ψ(K) so that $\Psi (K)=(P_0,\ldots ,P_d)$ (where the cell $\Delta _0\supset P_0^\perp$ is surrounded by the remaining *d* cells $\Delta _i\supset P_i^\perp$, with $1\le i\le d$).

For each $0\le i\le d$, we select a subset $P_i(K)\subseteq P_K\cap P_{i}$ of cardinality

$$ |P_i(K)|=\lceil \delta |P_K|/(4d+4)s\rceil . $$

Note that the secondary *t*-partition $\mathcal {P}_0=\mathcal {P}(P_{0},t)=\{P_{0,j}\mid 1\le j\le t\}$ of the part $P_0$ induces the partition $\mathcal {P}_0(K)=\{P_{0,j}(K)\mid 1\le j\le t\}$ of the subset $P_0(K)$, with $P_{0,j}(K):=P_{0,j}\cap P_0(K)$, for all $1\le j\le t$.

#### Definition

We say that the part $P_{0,j}\in \mathcal {P}_0$ is *full* with respect to *K* (or, simply, that the subset $P_{0,j}(K)$ is *full*) if we have that $|P_{0,j}(K)|\ge \epsilon _2n$.

It can be assumed, in what follows, that $\epsilon _2n\ge 1/c_2\ge 100$ for, otherwise, every singleton subset within $P_K$ is $(c_2\epsilon _2)$-heavy, and the entire family ${{\mathcal {K}}}(P,\epsilon )$ must have been pierced by the strong net $N_{strong}(P,c_2\epsilon _2)=P$ in Step 2.

In view of Lemma 5.3, every full subset $P_{0,j}(K)\in \mathcal {P}_0(K)$ must then satisfy

17 $$\begin{aligned} 100\le 1/c_2\le \epsilon _2n \le |P_{0,j}(K)|\le \epsilon _1n. \end{aligned}$$

We say that a 2-edge $\lambda \in {P\atopwithdelims ()2}$ is *short* if both its endpoints lie in the same part $P_{i,j}$ of the secondary partition $\mathcal {P}(P,s,t)$ of *P*.

**Fig. 18:**
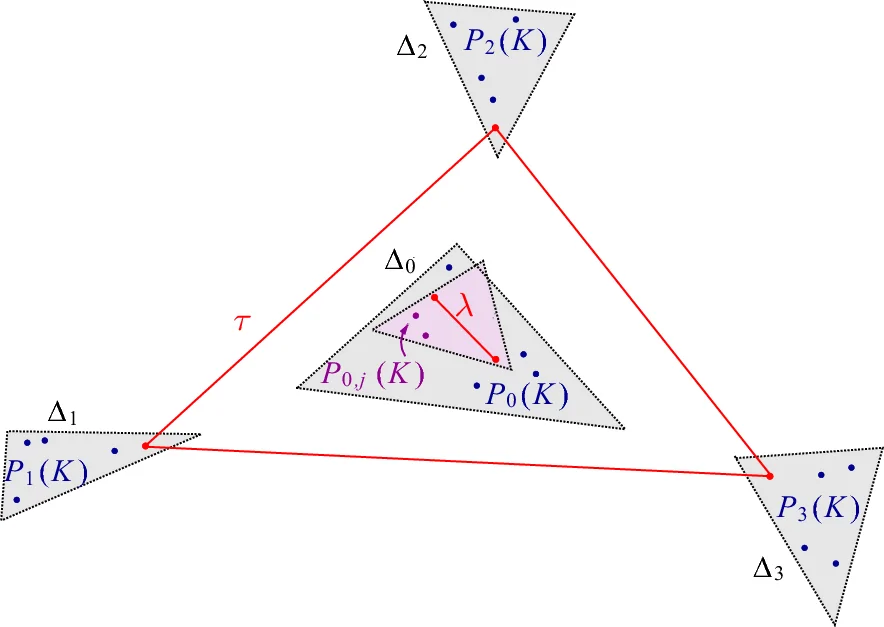
The overall setup for the case analysis of Step 3 – a schematic view from above in dimension d=3. Depicted are the sets $P_i(K)$ together with the enclosing cells $\Delta _i$ in $\mathcal {V}(P,s)$, a (d-1)-simplex $\tau \in \Sigma (K)$, and a short 2-edge $\lambda \in \Lambda _{0,j}(K)$. Notice that $\lambda ^\perp \subseteq \Delta _0\subseteq \tau ^\perp$ holds for any such pair $(\lambda ,\tau )\in \Lambda _{0,j}(K)\times \Sigma (K)$

**Flatness.** At the heart of our construction lies the relation between the following two classes of simplices, which are determined by the remaining convex sets $K\in {{\mathcal {K}}}_{>\delta }$; see Fig. 18.

A. The *t* families
  $$ \Lambda _{0,j}(K):={P_{0,j}(K)\atopwithdelims ()2}, $$
  each comprised of such short 2-edges λ such that both of their vertices come from a given subset $P_{0,j}(K)$ in the induced partition $\mathcal {P}_0(K)$ of $P_0(K)$, with $1\le j\le t$.
B. The family Σ(K) of all such simplices $\tau \in {P_K\atopwithdelims ()d}$ whose *d* vertices $p_i$ are chosen from the *d* different subsets $P_i(K)$ of $P_K$, with $1\le i\le d$. Namely,
  $$ \Sigma (K):\!=\!\left\{ {{\texttt{conv}}}\left( p_1,p_2,\!\ldots \!,p_d\right) \!\mid \! p_1\in P_1(K),p_2\!\in \! P_2(K),\!\ldots \!,p_{d}\in P_{d}(K)\right\} . $$

#### Remark

Since the ambient cell $\Delta _{0}$ of $P_0$ is surrounded by the *d* ambient cells $\Delta _i$ of the remaining sets $P_i$, with $1\le i\le d$, it follows that every pair $(\lambda ,\tau )\in \Lambda _{0,j}(K)\times \Sigma (K)$, with $1\le j\le t$, must satisfy $\lambda ^\perp \subset \Delta _0\subset \tau ^\perp$. See Fig. 18. In other words, the intersection of the supporting hyperplane H(τ) of τ with the vertical prism $\Delta _0^*$ over $\Delta _0$, must lie entirely within τ, so that every point of $P_{0}(K)$ lies either above or below τ.

To gain a little intuition, suppose that the convex set $K\in {{\mathcal {K}}}_{>\delta }$ at hand is a *hyperplane*. Then the respective set $P_0(K)$ (along with all the short 2-edges in $\biguplus _{i=1}^t\Lambda _{0,j}(K)$) is contained in *every* simplex $\tau \in \Sigma (K)$. In particular, the points of $P_{0}(K)$ must lie in at most $c_{ sp}t^{1-1/d}$ simplicial cells $\varphi _{0,j}$, which are crossed by every simplex $\tau \in \Sigma (K)$. Therefore, by the pigeonhole principle the majority of the points in $P_0(K)$ must belong to the full parts $P_{0,j}(K)\in \mathcal {P}_0(K)$, which satisfy

$$ |P_{0,j}(K)|\ge \frac{|P_0(K)|}{100\cdot c_{ sp}\cdot t^{1-1/d}}\ge \epsilon _2n. $$

A suitable infinitesimal perturbation of *P* puts the points of $P_K$ in a convex (and “sufficiently generic”) position, while placing the entire subset $P_0(K)$ either above, or below, of all the simplices $\tau \in \Sigma (K)$. As a result, every short edge $\lambda \in \biguplus _{i=1}^t\Lambda _{0,j}(K)$ is now “parallel” to every simplex $\tau \in \Sigma (K)$, in the sense that its supporting line ${{\texttt{aff}}}(\lambda )$ misses τ.

In what follows, we show that a very similar set of properties can be established for all the remaining convex sets $K\in {{\mathcal {K}}}_{>\delta }$ if (some of) the points in $\biguplus _{i=1}^d P_i(K)$ are replaced by their ambient simplicial cells φ(p) in $\mathcal {P}(P,s,t)$, and the possible “co-planarities” involving such cells φ(p) are taken into account.

#### Definition

For every convex set $K\in {{\mathcal {K}}}_{>\delta }$, and every simplex $\tau =\{p_1,\ldots ,p_d\}\in \Sigma (K)$, we use $\Phi (\tau )$ to denote the convex hull ${{\texttt{conv}}}\left( \bigcup _{i=1}^d\varphi (p_i)\right)$ of the ambient cells of the *d* vertices of τ, and refer to $\Phi (\tau )$ as the *envelope* of τ in $\mathcal {P}(P,s,t)$.^27^

#### Definition

Let $K\in {{\mathcal {K}}}_{>\delta }$ be a δ-punctured set with $\Psi (K)=(P_0,\ldots ,P_d)$, $\tau ={{\texttt{conv}}}(p_1,\ldots ,p_d)$ be a simplex in Σ(K), and λ be short 2-edge in $\Lambda _{0,j}(K)$.

**Fig. 19:**
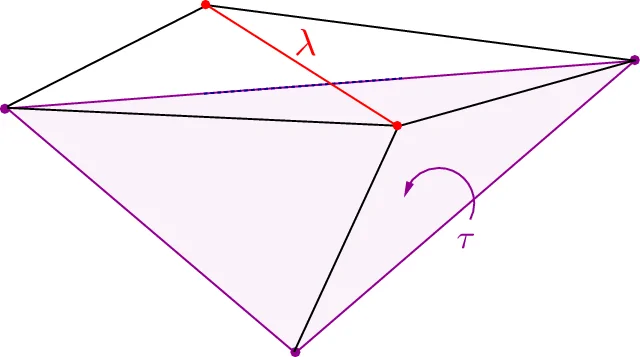
The 2-edge $\lambda \in \Lambda _{0,j}(K)$ is parallel to the simplex $\tau \in \Sigma (K)$ in $\mathbb {R}^3$, so they comprise facets of the convex pentahedron ${{\texttt{conv}}}(\tau \cup \lambda )$

**Fig. 20:**
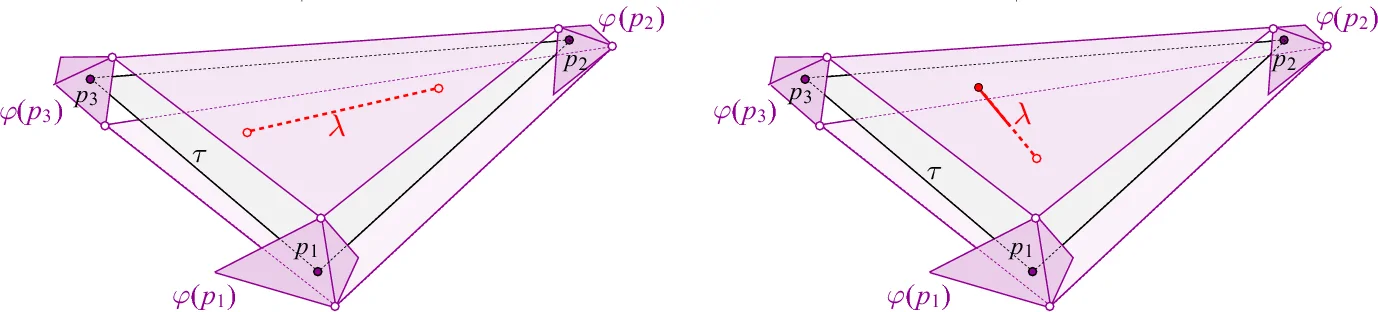
Left: A tight pair $(\lambda ,\tau )$ in $\mathbb {R}^3$. The short 2-edge $\lambda \in \Lambda _{0,j}(K)$ is contained in the envelope $\Phi (\tau )={{\texttt{conv}}}(\varphi (p_1)\cup \varphi (p_2)\cup \varphi (p_3))$. Right: A loose pair $(\lambda ,\tau )$ in $\mathbb {R}^3$. At least one of the vertices of the short 2-edge λ lies either above, or below $\Phi (\tau )$

1. We say that λ and τ are *parallel* if both λ and τ are faces of the convex “(d+2)-hedron” ${{\texttt{conv}}}(\tau \cup \lambda )$ (i.e., a convex polytope with d+2 vertices); see Fig. 19.
  Notice that the fact that $\lambda ^\perp \subset \tau ^\perp$ implies that the 2-edge λ must lie either entirely above, or entirely below τ, so that τ is always a facet of ${{\texttt{conv}}}(\tau \cup \lambda )$.
2. We say that the ordered pair $(\lambda ,\tau )$ is *tight* if λ and τ are parallel and, in addition, λ is contained in the envelope $\Phi (\tau )$ of τ; see Fig. 20 (left). (Notice that the *d* vertices of $\tau =\{p_1,\ldots ,p_d\}$ determine *d* distinct simplicial cells $\varphi (p_i)$, each within the respective secondary partition $\mathcal {P}_i=\mathcal {P}(P_i,t)$.)
  If such a pair $(\lambda ,\tau )\in \Lambda _{0,j}(K)\times \Sigma (K)$ is only parallel yet not tight, we say that it is *loose*, in which case at least one of the two vertices of λ lies either above or below $\Phi (\tau )$; see Fig. 20 (right).

#### Definition

Let $K\in {{\mathcal {K}}}_{>\delta }$ be a δ-punctured set with $\Psi (K)=(P_0,\ldots ,P_d)$.

1. We say that a full subset $P_{0,j}(K)$ in the induced partition $\mathcal {P}_0(K)$ of $P_0(K)$ is *tight* if at least $|\Lambda _{0,j}(K)|\cdot |\Sigma (K)|/80$ of the pairs $(\lambda ,\tau )\in \Lambda _{0,j}(K)\times \Sigma (K)$ are tight.
2. Let $\mathcal {T}_0(K)$ to denote the subset of all the tight parts $P_{0,j}(K)$ within $\mathcal {P}_0(K)$.
3. We say that the set *K* is *flat* if the tight parts $P_{0,j}(K)\in \mathcal {T}_0(K)$ encompass at least $|P_0(K)|/5$ points of $P_0(K)$.

**The net** $N_{>\delta ,3}$. Our small-size net the remaining sets $K\in {{\mathcal {K}}}_{>\delta }$ is an easy corollary of the following two properties, whose respective proofs are postponed to Sects. 5.5 and 5.6.

#### Lemma 5.5

Given a sufficiently small choice of the constant $c_2>0$ in Step 2, every remaining convex set $K\in {{\mathcal {K}}}_{>\delta }$, that was missed by the combination $N_{>\delta ,1}\cup N_{>\delta ,2}$, must be flat.

#### Lemma 5.6

There is a subset $\tilde{\Pi }=\tilde{\Pi }(P,\epsilon ,\delta ,s,t)\subseteq {P\atopwithdelims ()d}$ with the following properties:

(i) We have that
  $$ |\tilde{\Pi }|=O\left( \frac{n^d}{\delta \epsilon t^{2/d}}\right) . $$
(ii) Every flat convex set $K\in {{\mathcal {K}}}_{>\delta }$ satisfies
  18 $$\begin{aligned} \left| {P_K\atopwithdelims ()d}\cap \tilde{\Pi }\right| =\Omega \left( (\delta \epsilon n)^d/{s}^d\right) . \end{aligned}$$

According to Lemma 5.5 every remaining set $K\in {{\mathcal {K}}}_{>\delta }$ is flat and, by the second part of Lemma 5.6, satisfies (18). Hence, the final net $N_{>\delta ,3}$ for the all remaining sets in ${{\mathcal {K}}}_{>\delta }$ is provided by the following lemma, which is established by plugging $\tilde{\Pi }$ into Theorem 3.3, with the threshold $\sigma =\Omega \left( \delta ^d/s^d\right)$, and then using the upper bound on $|\tilde{\Pi }|$ in the first part of Lemma 5.6. (Notice that the last inequality follows by the choice of $s\lll _\eta u\lll _\eta t$, and $\delta =1/r^{d(d+1)+1}$, which in particular implies that $\delta ^d/s^d$ is much larger than $1/u^{\eta }$.)

#### Lemma 5.7

Suppose that the choice of the constant $c_2>0$ in Step 2 meets the criteria of Lemma 5.5. Then the remaining sets of ${{\mathcal {K}}}_{>\delta }$ can be pierced by a net $N_{>\delta ,3}$ of cardinality

19 $$\begin{aligned} \left| N_{>\delta ,3}\right| =O\left( u\cdot f\left( \epsilon \cdot u^{1/(d-1)}\cdot \delta ^{d-1}/s^{d-1}\right) +\frac{u^{(d-1)^2}\cdot s^{d\beta _{d-1}}}{\delta ^{d\beta _{d-1}+1}\epsilon ^{d+1} t^{2/d}}\right) = \end{aligned}$$

$$ =O\left( u\cdot f\left( \epsilon \cdot u^{1/(d-1)-\eta }\right) +\frac{1}{\epsilon ^{d+1+\eta }t^{2/d}}\right) . $$

#### Remark

As was explained in Sect. 2.6, the recursive term in (19) is of the “$O^*\left( 1/\epsilon ^{d-1}\right)$-type”, while the non-recursive term is smaller than $1/\epsilon ^d$ for any value of *t* that is considerably larger than $(1/\epsilon )^{d/2}$. To complete the proof of Theorem 4.1, in Sect. 5.7 we will combine the bounds in Lemmas 5.3, 5.4, and 5.7, and choose a suitable $t=(1/\epsilon )^{3d/4+o_d(1)}$.

### Proof of Lemma 5.5

The proofs of both Lemmas 5.5 and 5.6 will use the following basic property of tangent hyperplanes to convex polytopes.

#### Lemma 5.8

**Fig. 21:**
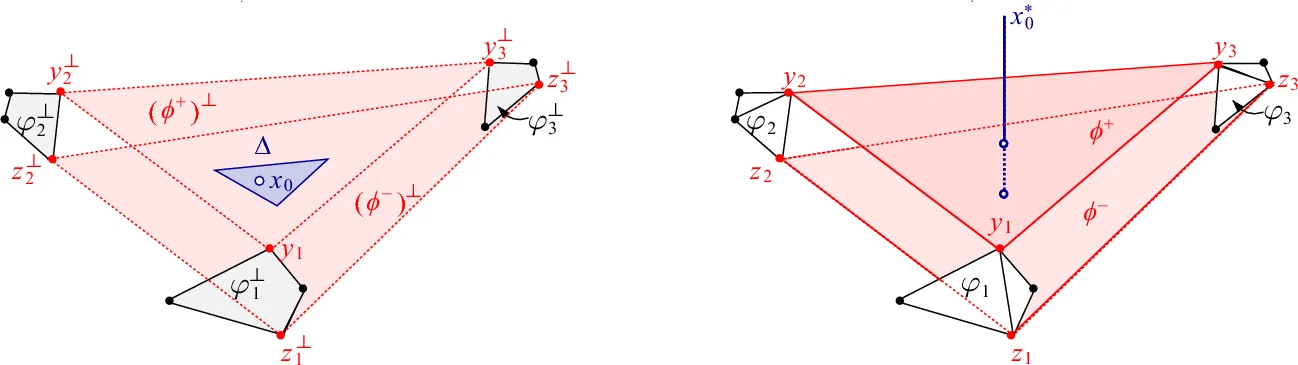
Lemma 5.8. Left: Δ is a subset of $\mathbb {R}^2$ which is surrounded by the vertical projections $\varphi ^\perp _1,\varphi _2^\perp$ and $\varphi ^\perp _3$ of the 3-dimensional polytopes $\varphi _1,\varphi _2$ and $\varphi _3$. Right: Any vertical line $x_0^*$ over a point $x_0\in \Delta$ crosses the same facets $\phi ^+$ and $\phi ^-$ which support, respectively, the upper and the lower common tangents to $\varphi _1,\varphi _2$ and $\varphi _3$

Let $\varphi _1,\ldots ,\varphi _{d}$ be a collection of *d* convex polytopes in $\mathbb {R}^d$ whose vertices are in general position, and Δ be a subset of $\mathbb {R}^{d-1}$ that is surrounded by their respective vertical projections $\varphi _1^\perp ,\ldots ,\varphi _d^\perp$ (see Fig. 21 (left)). Then there is a unique hyperplane $H^+$ (resp., $H^-$) that is tangent to each $\varphi _i$ from above (resp., below) at the respective point $y_i\in \varphi _i$ (resp., $z_i\in \varphi _i$), and so that $\Delta \subset {{\texttt{conv}}}(y_1,\ldots ,y_d)^\perp \cap {{\texttt{conv}}}(z_1,\ldots ,z_d)^\perp$.

In other words, any vertical line through Δ must cross the boundary of the polytope $\Phi :={{\texttt{conv}}}\left( \varphi _1\cup \ldots \cup \varphi _d\right)$ at the facets ${{\texttt{conv}}}(z_1,\ldots ,z_d)$ and ${{\texttt{conv}}}(y_1,\ldots ,y_d)$, which support, respectively, $H^-$ and $H^+$, and in this increasing order of the *d*-th coordinate.

#### Proof of Lemma 5.8

Since Δ is surrounded by $\varphi _i^\perp$, for $1\le i\le d$, it follows via Corollary 2.4 that the family $\Gamma :=\{\varphi ^\perp _1,\ldots ,\varphi ^\perp _d,\Delta \}$ of d+1 compact convex sets in $\mathbb {R}^{d-1}$ is separated. In particular, Δ cannot intersect any of the “(d-1)-wise” hulls ${{\texttt{conv}}}\left( \bigcup _{j\in [d]{\setminus } \{i\}}\varphi _i^\perp \right)$, for $1\le i\le d$ (or, else, some *d* elements of Γ, including Δ, would be intersected by the same (d-2)-flat in $\mathbb {R}^{d-1}$).

Fix a point $x_{0}\in \Delta$. The preceding discussion implies that the vertical line $x_0^*$ through $x_{0}$ must cross the boundary of the polytope $\Phi ={{\texttt{conv}}}\left( \varphi _1\cup \ldots \cup \varphi _d\right)$ at some pair of facets $\phi ^+$ and $\phi ^-$ whose supporting vertices come from *d* *distinct* polytopes $\varphi _i$, as depicted in Fig. 21 (right). Namely, we have that $\phi ^-=(y_1,\ldots ,y_d)$ and $\phi ^+=(z_1,\ldots ,z_d)$, where $y_i,z_i\in \partial \varphi _i$ for all $1\le i\le d$, and Φ lies below $H\left( \phi ^+\right)$ and above $H\left( \phi ^-\right)$.

Since Δ is surrounded by $\varphi _1^\perp ,\ldots ,\varphi _d^\perp$, it must be that $\Delta \subseteq (\phi ^+)^\perp \cap (\phi ^-)^\perp$, and, furthermore, the simplex $\phi ^+$ (resp., $\phi ^-$) supports the unique common upper (resp., lower) tangent $H^+=H(\phi ^+)$ (resp., $H^-=H(\phi ^-))$ to the polytopes $\varphi _1,\ldots ,\varphi _d$, which does not depend on the choice of $x_0$.^28^□

To establish Lemma 5.5, let us fix a convex set $K\in {{\mathcal {K}}}_{>\delta }$, with the principal (d+1)-tuple $\Psi (K)=(P_0,P_1,\ldots ,P_d)$, and assume that *K* is *not* flat. That is, the tight subsets $P_{0,j}(K)\in \mathcal {T}_0(K)$ encompass fewer than $|P_0(K)|/5$ points of $P_0(K)$, whereas the remaining subsets $P_{0,j}(K)\in \mathcal {P}_0(K)\setminus \mathcal {T}_0(K)$ encompass more than $\frac{4}{5}|P_0(K)|$ points of $P_0(K)$. To establish the lemma, it suffices to show that, pending a sufficiently small choice of the constant $c_2>0$ in Step 2, such a set *K* must have been pierced by the combination $N_{>\delta ,1}\cup N_{>\delta ,2}$ of previous nets, and subsequently removed from ${{\mathcal {K}}}_{>\delta }$.

In view of our choice of ε in (7), and the lower bound $n_0(\epsilon )$ on *n* in (8), we have that

20 $$\begin{aligned} |P_0(K)|\ge \frac{\delta \lceil \epsilon n\rceil }{(4d+4)s} \ge \frac{10^6\varepsilon \lceil n/s\rceil }{\vartheta }\ge \max \left\{ 10^5\cdot \frac{\varepsilon |P_0|}{\vartheta },10^6d\right\} . \end{aligned}$$

Also note that Lemma 5.8 yields a unique pair of, respectively, upper and lower common tangent hyperplanes $H^+$ and $H^-$ to the *d* polytopes ${{\texttt{conv}}}\left( P_i(K)\right)$, with $1\le i\le d$. Specifically, $H^+$ (resp., $H^-$) meets the polytopes ${{\texttt{conv}}}\left( P_i(K)\right)$ at the respective vertices $a_i\in P_i(K)$ (resp., $b_i\in P_i(K)$), which determine the simplex $\tau ^+={{\texttt{conv}}}(a_1,\ldots ,a_d)$ (resp., $\tau ^-={{\texttt{conv}}}(b_1,\ldots ,b_d)$) in Σ(K). Also note that the “inner” cell $\Delta _0$, which encloses the projection of $P_0(K)$, lies in the intersection $(\tau ^+)^\perp \cap (\tau ^-)^\perp$ of the respective projections of $\tau ^+$ and $\tau ^-$. Hence, every point $p\in P_0(K)$ is either contained in the polytope $I_0(K)={{\texttt{conv}}}(\tau ^+\cup \tau ^-)\subseteq K$ with at most 2*d* vertices, or it lies to the same side (either above or below) of both simplices $\tau ^+$ and $\tau ^-$.

**Fig. 22:**
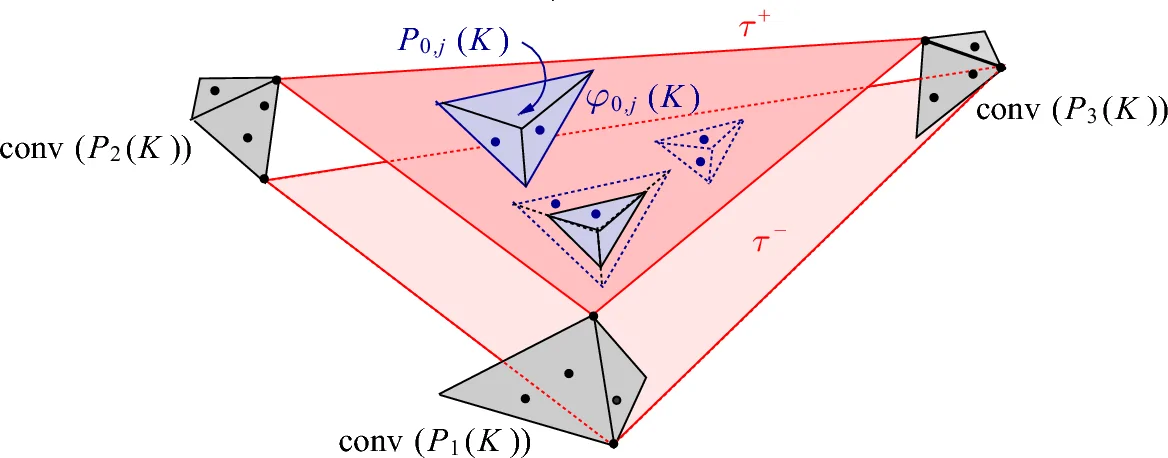
Proof of Lemma 5.5 in $\mathbb {R}^3$. The simplex $\tau ^+$ (resp.., $\tau ^-$) supports the upper (resp., lower) common tangent hyperplane to ${{\texttt{conv}}}\left( P_1(K)\right) ,{{\texttt{conv}}}(P_2(K))$, and ${{\texttt{conv}}}\left( P_3(K)\right)$. Three classes of subsets $P_{0,j}\in \mathcal {P}_0(K)$ are illustrated, together with their enclosing cells $\varphi _{0,j}$

The preceding discussion implies that any part $P_{0,j}(K)$ in the induced partition $\mathcal {P}_0(K)$ of $P_0(K)$ must belong to at least one of the following families (see Fig. 22):

1. the family $\mathcal {I}_0(K)$, which consists of all such subsets $P_{0,j}(K)\in \mathcal {P}_0(K)$ that are contained in the aforementioned “(2*d*)-hedron” $I_0(K)={{\texttt{conv}}}\left( \tau ^+\cup \tau ^-\right)$ within *K*,
2. the family $\mathcal {Z}_0(K)$, which is comprised of all such subsets $P_{0,j}(K)$ that fall in the zone of either one, or both, of the hyperplanes $H^+$ and $H^-$ (i.e., their ambient cells $\varphi _{0,j}$ in $\mathcal {P}_0$ intersect $H^+\cup H^-$),^29^ or
3. the family $\mathcal {P}_0^+(K)$ (resp., $\mathcal {P}_0^-(K)$) which is comprised of all such subsets $P_{0,j}(K)$ that lie above $\tau ^+$ (resp., below $\tau ^-$) and, furthermore, their enclosing cells $\varphi _{0,j}$ in $\mathcal {P}_0$ lie entirely above $H^+$ (resp., entirely below $H^-$).

To proceed, we distinguish between three basic scenarios for $K\in {{\mathcal {K}}}_{>\delta }$, based on the distribution of the points of $P_0(K)$ within its partition $\mathcal {P}_0(K)$ that is induced by $\mathcal {P}_0=\mathcal {P}(P_0,t)$.

**Case (a).** If at least $c_2\epsilon _2n$ points of $P_0(K)$ belong to $\bigcup \mathcal {I}_0(K)$ (in particular, they are “sandwiched” between $\tau ^+$ and $\tau ^-$), then the polytope $I_0(K)$ is $(c_2\epsilon _2)$-heavy, so *K* must have been pierced in Step 2 by the net $N_{strong}(P,c_2\epsilon _2)$.

Hence, it can be assumed, from now on, that case (a) does not occur, that is,

21 $$\begin{aligned} \left| \bigcup \mathcal {I}_0(K)\right| <c_2\epsilon _2n. \end{aligned}$$

**Case (b).** If the *non-full* parts of $\mathcal {P}_0(K)$ encompass at least $|P_0(K)|/10$ points of $P_0(K)$, we argue that, pending a suitable choice of $c_2>0$, such a convex set $K\in {{\mathcal {K}}}_{>\delta }$ must have been pierced by some 1-dimensional net $N'_{\ell _0}\subseteq N_{>\delta ,2}$, which has been constructed Step 2 for some line $\ell _0$ in the secondary ϑ-canonical subset $\mathcal {{L}}_0=\mathcal {{L}}(P_0,u)$ of $P_0$.

To this end, note that, according to Theorem 2.6, the zones of the hyperplanes $H^+$ and $H^-$ together encompass at most $2c_{ sp}t^{1-1/d}$ non-full parts of $\mathcal {Z}_0(K)$, whose ambient cells $\varphi _{0,j}$ cross $H^+\cup H^-$, so that the union of these non-full parts accounts for fewer than $2c_{ sp}t^{1-1/d}\epsilon _2n$ points of $P_0(K)$. Together with (20) and (21) (and with our prior choice (16) of $\epsilon _2$, and of $c_2<1/100$), this implies that the non-full parts $P_{0,j}(K)$ of $\mathcal {P}_0^+(K)\uplus \mathcal {P}_0^-(K)$ account for at least

$$ \frac{|P_0(K)|}{10}\!-\!c_2\epsilon _2n\!-\!2c_{ sp}t^{1-1/d}\epsilon _2n\!\ge \! \frac{|P_0(K)|}{10}\!-\!\frac{\delta \epsilon n\left( c_2\!+\!2c_{ sp}\!\cdot \! t^{1-1/d}\right) }{c_{ sp}\cdot (100d\!+\!100)st^{1-1/d}}\!\ge \! \frac{|P_0(K)|}{20} $$

points of $$P_0(K)$$.

It thus can be assumed, with no loss of generality, that the non-full parts of $\mathcal {P}_0^+(K)$ encompass a subset $P_0^+(K)$ that lies entirely above $\tau ^+$, and whose cardinality satisfies

$$ |P_0^+(K)|\ge |P_0(K)|/40\ge \max \left\{ \varepsilon |P_0|/\vartheta ,10^4d\right\} , $$

with the last inequality following from the previous lower estimate (20) on $|P_0|$. Also note that $P^+_0(K)$ determines, within ${P_0\atopwithdelims ()d}$, the non-empty sub-family $\Pi _0^+(K):={P_0^+(K)\atopwithdelims ()d}$ whose cardinality satisfies $|\Pi _0^+(K)|\ge 80^{-d}{|P_0(K)|\atopwithdelims ()d}$.

**Fig. 23:**
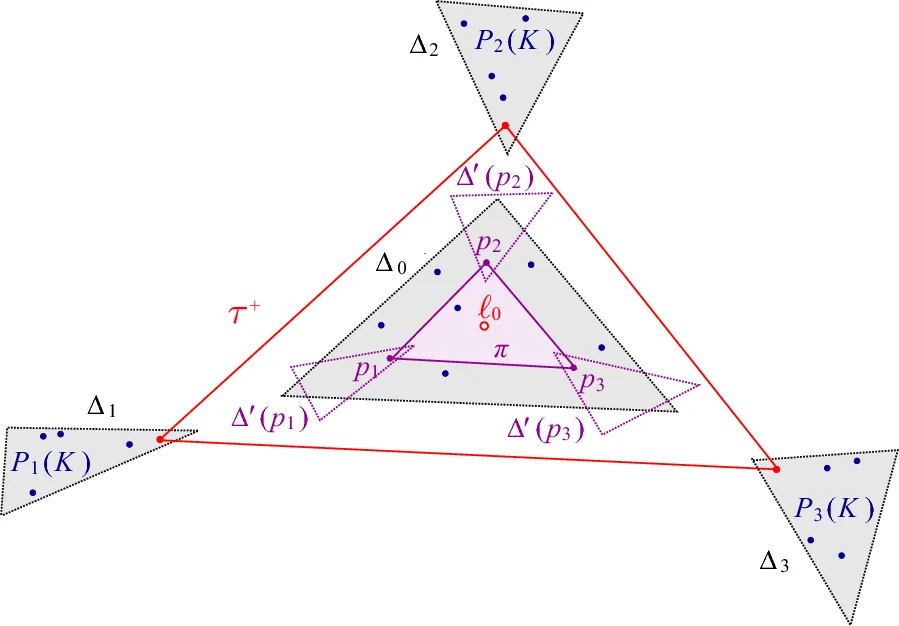
Proof of Lemma 5.5 in $\mathbb {R}^3$. Case (b) – a schematic view from above. Depicted are the vertical line $\ell _0\in \mathcal {{L}}(P_0,u)$, a simplex $\pi ={{\texttt{conv}}}(p_1,p_2,p_3)\in \Pi ^+_{\ell _0}$. Since all of the vertices of π lie above $\tau ^+$, the intersection $\ell _0\cap \tau ^+$ lies below $\ell _0\cap \pi$. In the secondary vertical partition $\mathcal {V}(P_0,u)$ of $P_0$, the line $\ell _0$ is surrounded by the ambient cells $\Delta '(p_1),\Delta '(p_2)$ and $\Delta '(p_3)$ of, respectively, $p_1,p_2$ and $p_3$

Consider the secondary partition $\mathcal {V}_0=\mathcal {V}(P_0,u)$ of $P_0$, and the secondary ϑ-canonical line family $\mathcal {{L}}_0=\mathcal {{L}}(P_0,u)$ which has been constructed in Step 2 over $\mathcal {V}(P_0,u)$. Since the set *K* is ε-spread in $\mathcal {V}(P_0,u)$, and at least $\varepsilon |P_0|/\vartheta$ of its points belong to $P_0$, there must exist a line $\ell _0\in \mathcal {{L}}_0$, with a subset $\Pi ^+_{\ell _0}\subseteq \Pi ^+_0(K)$ of at least $\vartheta |\Pi ^+_0(K)|$ simplices, that together meet the following criteria (see Fig. 23):

(i) For each simplex $\pi =\{p_1,\ldots ,p_d\}\in \Pi ^+_{\ell _0}$, the line $\ell _0$ is surrounded, within the secondary vertical partition $\mathcal {V}_0=\mathcal {V}(P_0,u)$, by the ambient (d-1)-dimensional cells $\Delta '(p_1),\ldots ,\Delta '(p_d)$ of the vertices of π.
(ii) $\ell _0$ also crosses $\tau ^+$ at a point which lies below all its intersections with the simplices of $\Pi ^+_{\ell _0}$. (This is because $\ell _0^\perp$ lies in the subset ${{\texttt{conv}}}\left( P_0^\perp \right) \subseteq \Delta _0$, which is contained in $(\tau ^+)^\perp$, while all of the simplices π in $\Pi _{\ell _0}^+$ are determined by the set $\bigcup \mathcal {P}_0^+(K)$, which lies above $\tau ^+$.)

Fix a simplex $\pi ={{\texttt{conv}}}(p_1,\ldots ,p_d)$ of $\Pi ^+_{\ell _0}$. Note that, by the choice of $\mathcal {P}_0^+(K)$, and of the point set $P_0^+(K)\subseteq \bigcup \mathcal {P}_0^+(K)$, the *d* ambient clipped cells $\varphi '_1=\varphi '(p_1),\ldots ,\varphi '_d=\varphi '(p_d)$ of the vertices of π, must lie above $\tau ^+$, as each of them is contained in the prism $\Delta ^*_0$ over $\Delta _0$. Furthermore, the vertical projections of $\varphi '_1,\ldots ,\varphi '_d$ must surround $\ell _0$, as each of these cells $\varphi '_i$ has been “clipped” to the vertical prism $(\Delta '(p_i))^*$ over the ambient (d-1)-dimensional cell $\Delta '(p_i)$ of $p_i$ in $\mathcal {V}(P_i,u)$.^30^

**Fig. 24:**
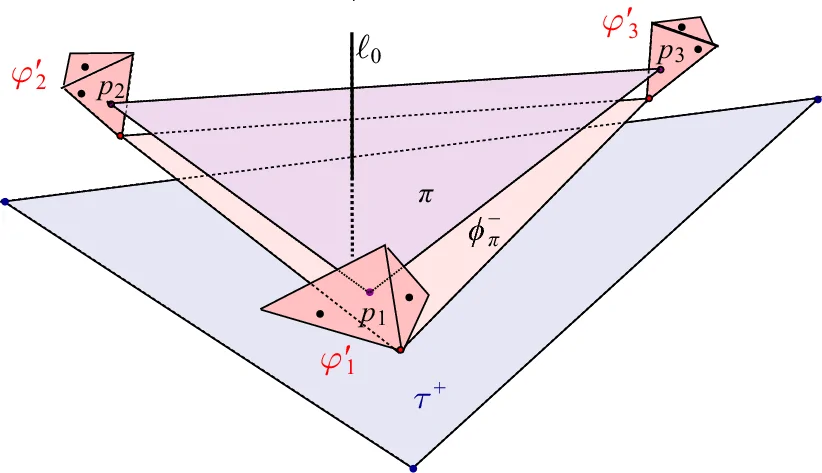
Proof of Lemma 5.5 in $\mathbb {R}^3$ – case (b). Showing that the vertical line $\ell _0\in \mathcal {{L}}_i$ crosses $\phi ^-_\pi$ within $K\cap \ell _0$. The line $\ell _0$ crosses the simplices $\pi \in \Pi ^+_{\ell _0}$, $\phi ^-_\pi$, and $\tau ^+$ in this decreasing order of the last coordinate

Consider the polytope $\Phi '(\pi ):={{\texttt{conv}}}\left( \varphi '_1\cup \ldots \cup \varphi '_d\right)$. Applying Lemma 5.8 (with $\Delta =\{\ell ^\perp _0\}$) yields a boundary (d-1)-simplex $\phi ^-_\pi$ of $\Phi '(\pi )$ whose supporting hyperplane is tangent to $\varphi '_1,\ldots ,\varphi '_d$ from below, and whose vertical projection $(\phi ^-_\pi )^\perp$ contains $\{\ell _0^\perp \}$. In particular, this simplex $\phi _\pi ^-$ is too crossed by the vertical line $\ell _0$. See Fig. 24.

Furthermore, recalling the notation of Sect. 5.3, the simplex $\phi ^-_\pi$ belongs to $V'(p_1)\times \ldots \times V'(p_d)$, thus meeting $\ell _0$ at a point of $X'_{\ell _0}$. Since (i) all the vertices of $\phi _\pi ^-\subset {{\texttt{conv}}}\left( \varphi '_1\cup \ldots \cup \varphi '_d\right)$ lie above $H^+$, and (ii) all the vertices of π lie above the lower tangent hyperplane $H\left( \phi ^-_\pi \right)$ to $\Phi '(\pi )$, this intersection point $\phi ^-_\pi \cap \ell _0\in X'_{\ell _0}$ must lie between the points $\ell _0\cap \tau ^+=\ell _0\cap H^+$ and $\ell _0\cap \pi$ and, thus, within the interval $K\cap \ell _0$ (as both τ and π belong ${P_K\atopwithdelims ()d}$).

To recap, every simplex $\pi ={{\texttt{conv}}}(p_1,\ldots ,p_d)\in \Pi ^+_{\ell _0}$ has been assigned the unique simplex $$\phi ^-_\pi \in \left( \begin{array}{l}V' \\ d\end{array}\right) $$ which supports the common lower tangent $H(\phi ^-_\pi )$ to $\varphi '(p_1),\ldots ,\varphi '(p_d)$, and intersects $\ell _0$ at the point $\phi ^-_\pi \cap \ell _0\in X'_{\ell _0}\cap K$. Since any simplex $$\phi ={{\texttt{conv}}}(v_1,\ldots ,v_d)\in \left( \begin{array}{l}V' \\ d\end{array}\right) $$ is “assigned” in this manner to at most $\lfloor \epsilon _2n\rfloor ^d$ simplices $\pi =(p_1,\ldots ,p_d)\in \Pi ^+_{\ell _0}$, which satisfy $\phi ^-_\pi =\phi$ (as the choice of $v_1,\ldots ,v_d$ uniquely determines the *d* ambient *non-full* parts of $p_1,\ldots ,p_d$ within $\mathcal {P}_0^+(K)$), the interval $\ell _0\cap K$ must contain at least

$$ \frac{|\Pi ^+_{\ell _0}|}{\left( \lfloor \epsilon _2 n \rfloor \right) ^d}=\Theta \left( \frac{(\delta \epsilon n/s)^d}{\left( \delta \epsilon n/\left( st^{1-1/d}\right) \right) ^d}\right) =\Theta \left( t^{d-1}\right) \gg \frac{t^{d-d/\alpha _d}}{u^{(d-1)^2}} $$

points $X_{\ell _0}$. Thus, given a sufficiently small choice of the constant $c_2>0$ in Step 2, which underlies the definition of the nets $N'_{\ell }$, the interval $\ell _0\cap K$ must contain a point of the net $N'_{\ell _0}\subset N_{>\delta ,2}$.

**Case (c).** We may from now on assume that the convex set $K\in {{\mathcal {K}}}_{>\delta }$ at hand falls into neither of the previous scenarios (a) and (b). Therefore, the full parts $P_{0,j}(K)$ of $\mathcal {P}_0(K)$ must encompass at least $9|P_0(K)|/10$ points of $P_0(K)$.

Since the polytope $I_0(K)={{\texttt{conv}}}\left( \tau ^-\cup \tau ^+\right)$, within *K*, encompasses at most $c_2\epsilon _2n$ points of $P_0(K)$ (or, else, such a set *K* would have been hit in Step 2 by the net $N_{strong}(P,c_2\epsilon _2)$), each of the full parts $P_{0,j}(K)$ must contain at least $|P_{0,j}(K)|-c_2\epsilon _2n\ge 2|P_{0,j}(K)|/3$ such points that lie outside $I_0(K)$ (either above $\tau ^+$ or below $\tau ^-$). For each of these full parts $P_{0,j}(K)$, it must be that either (i) at least $|P_{0,j}(K)|/3$ of its points lie above the simplex $\tau ^+$ (and, in particular, above the supporting hyperplane $H^+=H(\tau ^+)$), or (ii) at least $|P_{0,j}(K)|/3$ of its points lie below $\tau ^-$ (and, in particular, below $H^-=H(\tau ^-)$).

It can thus be assumed, with no loss of generality, that at least $2|P_0(K)|/5$ of the points of $P_0(K)$ fall in the full subsets $P_{0,j}(K)\in \mathcal {P}_0(K)$ of the former kind (so that at least $|P_{0,j}(K)|/3$ of their points lie above $\tau ^+$ and $H^+$). Let $\mathcal {F}_0(K)$ denote the collection of these subsets $P_{0,j}(K)$, so that

22 $$\begin{aligned} \left| \bigcup \mathcal {F}_0(K)\right| \ge 2|P_0(K)|/5. \end{aligned}$$

For every part $P_{0,j}(K)\in \mathcal {F}_0(K)$, let $P_{0,j}^+(K)$ denote its subset of at least

$$ |P_{0,j}(K)|/3\ge \epsilon _2n/3\ge 30 $$

points that lie *above* $H^+$ and $\tau ^+$ and, therefore, above every simplex $\tau \in \Sigma (K)$. (The second inequality uses (17).)

Any such full part $P_{0,j}(K)$ in $\mathcal {F}_0(K)$ determines, within $\Lambda _{0,j}(K)$, the sub-family

$$ \Lambda ^+_{0,j}(K):={P^+_{0,j}(K)\atopwithdelims ()2} $$

which is comprised of at least $|\Lambda _{0,j}(K)|/20$ short 2-edges that lie above $\tau ^+$. If such a part $P_{0,j}(K)$ belongs to $\mathcal {P}_0(K)\setminus \mathcal {T}_0(K)$ then, in particular, fewer than $\frac{1}{4}|\Lambda ^+_{0,j}(K)|\cdot |\Sigma (K)|$ of the pairs $(\lambda ,\tau )\in \Lambda _{0,j}^+(K)\times \Sigma (K)$ are tight. As a result, at least one of the following two scenarios has to occur for $P_{0,j}(K)$:

(i) at least $|\Lambda _{0,j}^+(K)|\cdot |\Sigma (K)|/2$ of the pairs $(\lambda ,\tau )\in \Lambda _{0,j}^+(K)\times \Sigma (K)$ are not parallel, or
(ii) at least $|\Lambda _{0,j}^+(K)|\cdot |\Sigma (K)|/4$ of the pairs in $(\lambda ,\tau )\in \Lambda _{0,j}^+(K)\times \Sigma (K)$ are parallel, yet not tight.

Let $\mathcal {F}'_0(K)$ (resp., $\mathcal {F}''_0(K)$) denote the sub-family of *all* such full subsets $P_{0,j}(K)\in \mathcal {F}_0(K)$ that satisfy condition (i) (resp., criterion (ii)).^31^ Using that $|\bigcup \mathcal {T}_0(K)|\le |P_0(K)|/5$, $\mathcal {F}_0(K)\setminus \mathcal {T}_0(K)\subseteq \mathcal {F}'_0(K)\cup \mathcal {F}''_0(K)$, and (22), we conclude that

$$ \left| \bigcup \mathcal {F}'_0(K)\cup \mathcal {F}''_0(K)\right| \ge \left| \bigcup \mathcal {F}_0(K)\right| -\left| \bigcup \mathcal {T}_0(K)\right| \ge |P_0(K)|/5. $$

To complete the proof of Lemma 5.5, we distinguish between two possible sub-scenarios for *K*.

**Case (c1).** If at least one subset $P_{0,j}(K)\in \mathcal {F}_0(K)$ belongs to $\mathcal {F}'_0(K)$, we again argue that such a set *K* must have been pierced by the net $N_{strong}(P,c_2\epsilon _2)\subseteq N_{>\delta ,2}$ (again, pending a suitably small choice of the constant $c_2>0$ in Step 2).

**Fig. 25:**
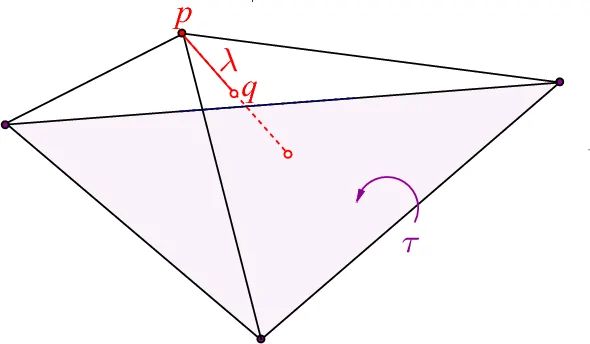
Proof of Lemma 5.5 in $\mathbb {R}^3$ – case (c1). The 2-edge λ lies above τ. Since λ and τ are *not* parallel, a vertex *q* of λ must lie in the convex hull of τ and the remaining vertex of λ

To this end, fix a non-parallel pair $(\lambda ,\tau )$, with $\lambda \in \Lambda ^+_{0,j}(K)$ and $\tau \in \Sigma (K)$. Since τ is still a boundary facet of the polytope ${{\texttt{conv}}}(\tau \cup \lambda )$ (as the short 2-edge λ lies in the halfspace above H(τ)), there must be a vertex $p\in \lambda$ so that ${{\texttt{conv}}}(\tau \cup \kappa )={{\texttt{conv}}}(\tau \cup \{p\})$; see Fig. 25. Thus, ${{\texttt{conv}}}(\tau \cup \kappa )$ must contain the remaining vertex *q* of λ. We, therefore, assign any such non-parallel pair $(\lambda ,\tau )\in \Lambda ^+_{0,j}(K)\times \Sigma (K)$ the “smaller” configuration (p,τ), which involves only d+1 vertices, so that the “missing” vertex *q* of the short 2-edge λ lies in the interior of ${{\texttt{conv}}}\left( \tau \cup \{p\}\right)$.

Since the set $P_{0,j}^+(K)\times \Sigma (K)$ encompasses only $O\left( \left| P_{0,j}^+(K)\right| \cdot \left| \Sigma (K)\right| \right)$ possible configurations, which we have assigned to a total of $\Theta \left( \left| P_{0,j}^+(K)\right| ^2\cdot \left| \Sigma (K)\right| \right)$ pairs $(\lambda ,\tau )$, the pigeonhole principle yields such a configuration (p,τ) that has been assigned to $\Omega \left( \left| P_{0,j}^+(K)\right| \right) =\Omega (\epsilon _2n)$ distinct pairs $(\lambda =\{p,q\},\tau )$, each adding a distinct vertex $q\in P_{0,j}^+(K)$ into the interior of the simplex ${{\texttt{conv}}}\left( \tau \cup \{p\}\right)$. Given a small enough choice of $c_2>0$ in Step 2, this simplex must contain at least $c_2\epsilon _2n$ points of $P_K$. However, such a convex set $K\in {{\mathcal {K}}}_{>\delta }$ must have been pierced by the strong net $N_{strong}(P,c_2\epsilon _2)\subseteq N_{>\delta ,2}$, and removed in Step 2.

**Case (c2).** Let us assume, at last, that $\mathcal {F}'_0(K)=\emptyset$ and, therefore,

$$ \left| \bigcup \mathcal {F}''_0(K)\right| \ge |P_0(K)|/5. $$

Once again, we will find a canonical line $\ell _0\in \mathcal {{L}}_0=\mathcal {{L}}(P_0,u)$ so that, given a suitably small constant $c_2>0$ in Step 2, the interval $K\cap \ell _0$ must contain a point of the net $N'_{\ell _0}\subset N_{>\delta ,2}$.

**Fig. 26:**
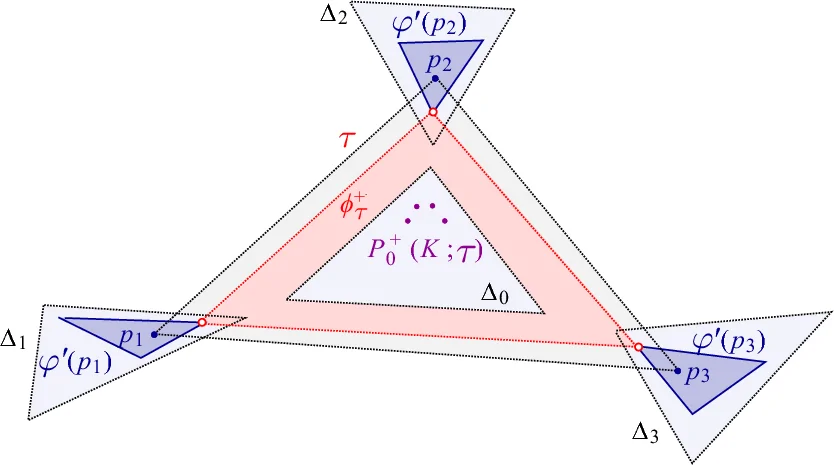
Proof of Lemma 5.5 in $\mathbb {R}^3$. Case (c2) – a schematic view from above. Depicted is a simplex $\tau \in \Sigma (K)$. The projections of the clipped cells $\varphi '(p_1),\varphi '(p_2)$ and $\varphi '(p_3)$ surround the simplicial cell $\Delta _0$, which contains the set $P_0(K)$. Depicted are the “upper” facet $\phi ^+_\tau$ of $\Phi '(\tau ):={{\texttt{conv}}}\left( \varphi '(p_1)\cup \varphi '(p_2)\cup \varphi '(p_3)\right)$, and the subset $P^+_0(K;\tau )$ of all such points in $P^+_0(K)\subseteq P_0(K)$ that lie above $\phi ^+_\tau$

To this end, fix a simplex $\tau ={{\texttt{conv}}}(p_1,\ldots ,p_d)\in \Sigma (K)$, so that $p_i\in P_i(K)$ for all $1\le i\le d$. Refer to Fig. 26. Note that the vertices of τ “mark” *d* ambient clipped cells $\varphi '(p_1),\ldots ,\varphi '(p_d)$, whose respective projections are contained in the respective cells $\Delta _1,\ldots ,\Delta _d$, which surround $\Delta _0$ (so that $\Delta _0$ is, in particular, surrounded by the projections $\varphi '(p_1)^\perp ,\ldots ,\varphi '(p_d)^\perp$).

Once again, let us consider the “clipped envelope” of τ

$$ \Phi '(\tau )={{\texttt{conv}}}\left( \varphi '(p_1)\cup \ldots \cup \varphi '(p_d)\right) , $$

which is obviously contained in $\Phi (\tau )={{\texttt{conv}}}\left( \bigcup _{i=1}^d\varphi (p_i)\right)$. According to Lemma 5.8, $\Phi '(\tau )$ has a unique facet $$\phi ^+_\tau \in {\Bigg (\begin{array}{l}V' \\ d\end{array}\Bigg )}$$ which supports the common upper tangent $H^+_\tau =H(\phi _\tau )$ to $\varphi '(p_1),\ldots ,\varphi '(p_d)$, with the property that $\Delta _0\subseteq (\phi _\tau ^+)^\perp$ (hence, every point of $P_0(K)$ lies either above or below the simplex $\phi ^+_\tau$). We thus assign $\phi ^+_\tau$ to τ, and use $P_{0}^+(K;\tau )$ to denote the subset of all such points in $P_{0}^+(K)$ that lie above the respective simplex $$\phi ^+_\tau \in {\Bigg (\begin{array}{l}V' \\ d\end{array}\Bigg )}$$ of τ.

In view of (17), any simplex of $$\phi \in {\Bigg (\begin{array}{l}V' \\ d\end{array}\Bigg )}$$ can be assigned in this manner at most $\lfloor \epsilon _1 n\rfloor ^d$ simplices $\tau \in \Sigma (K)$, which all satisfy $\phi =\phi _\tau ^+$.^32^ At the center of our argument lies the following property.

#### Lemma 5.9

With the previous choice of the convex set $K\in {{\mathcal {K}}}_{>\delta }$ that falls into case (c2), the family Σ(K) contains a subset $\tilde{\Sigma }(K)$ of at least $|\Sigma (K)|/10^5$ simplices $\tau \in \Sigma (K)$ that each satisfy $\left| P_0^+(K;\tau )\right| \ge \left| P_0(K)\right| /10^5$.

#### Proof of Lemma 5.9

By the pigeonhole principle, it is enough to find $|\Sigma (K)|\cdot |P_0(K)|/10^4$ such pairs $(\tau ,p)\in \Sigma (K)\times P^+_0(K)$ that satisfy $p\in P_0^+(K;\tau )$. Furthermore, as the parts of $\mathcal {F}''_0(K)$ cover at least $|P_0(K)|/5$ points of $P_0(K)$, it suffices to show that any part $P_{0,j}(K)\in \mathcal {F}_0(K)$ determines at least $|\Sigma (K)|\cdot |P_{0,j}(K)|/2000$ pairs $(\tau ,p)\in \Sigma (K)\times P_{0,j}(K)$, in which *p* lies above $\phi _\tau ^+$.

To see the latter claim, we keep the subset $P_{0,j}(K)\in \mathcal {F}''_0(K)$ fixed, and use $\Upsilon _{j}$ to denote the associated subset of at least $\left| \Lambda ^+_{0,j}(K)\right| \cdot \left| \Sigma (K)\right| /4$ parallel, albeit loose pairs $(\lambda ,\tau )\in \Lambda _{0,j}^+(K)\times \Sigma (K)$. The crucial observation is that, in any pair $(\lambda ,\tau )\in \Upsilon _{j}$, at least one of the vertices of λ must lie above $\Phi '(\tau )$ and, thereby, also above $\phi ^+_\tau$, since (i) λ lies above τ, (ii) $\Phi '(\tau )$ is contained in the envelope $\Phi (\tau )$ of τ, which does not contain λ (as the pair $(\lambda ,\tau )$ is loose), and (iii) we have that $\lambda ^\perp \subseteq \Delta _0^\perp \subseteq (\phi ^+_\tau )^\perp$ (so that every vertex of λ that lies outside $\Phi '(\tau )\supset \tau$, has to lie above $\phi ^+_\tau$).

By the pigeonhole principle, there is a sub-collection $\tilde{\Sigma }_j(K)$ of at least |Σ(K)|/16 simplices $\tau \in \Sigma (K)$, so that each of them is part of at least $\left| \Lambda ^+_{0,j}(K)\right| /16$ loose pairs $(\lambda ,\tau )\in \Upsilon _j$. Fix $\tau \in \tilde{\Sigma }_j(K)$. Then any short 2-edge $\lambda \in \Lambda _{0,j}^+(K)$, that completes a loose pair $(\lambda ,\tau )\in \Upsilon _j$ with τ, must include at least one vertex of $P^+_0(K;\tau )$ (as depicted in Fig. 20 (left)). Therefore, to allow at least $$\left| \Lambda ^+_{0,j}(K)\right| /16=\left( \begin{array}{c}|P_{0,j}^+(K)| \\ 2\end{array}\right) /16$$ choices of $\lambda \in \Lambda _{0,j}^+(K)$ as above, the subset $P_{0,j}^+(K;\tau )$ must contain at least $\left| P_{0,j}^+(K)\right| /32\ge \left| P_{0,j}(K)\right| /96$ points which lie above $\phi _\tau ^+$. Repeating this argument for at least |Σ(K)|/16 simplices $\tau \in \tilde{\Sigma }_j(K)$ yields a total of at least $|\Sigma (K)|\times |P_{0,j}(K)|/2000$ pairs $(\tau ,p)\in \Sigma (K)\times P_{0,j}^+(K)$ in which $p\in P_0^+(K,\tau )$. □

**Fig. 27:**
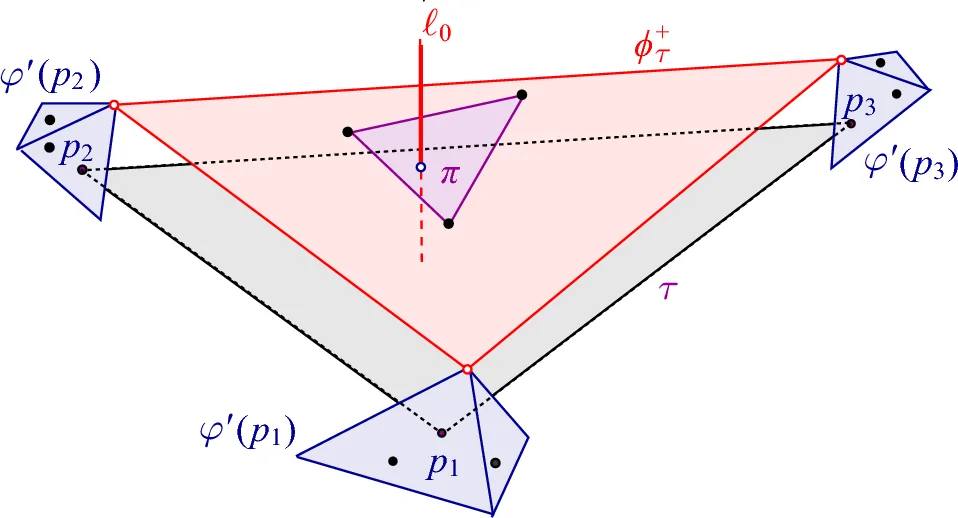
Proof of Lemma 5.5 in $\mathbb {R}^3$ – case (c2). Arguing that the intersection $\phi _\tau ^+\cap \ell _0$ lies in $K\cap \ell _0$. The line $\ell _0$ crosses the triangles $$\pi \in {\Bigg (\begin{array}{c}P^+_0(K,\tau ) \\ 3\end{array}\Bigg )}$$, $\phi _\tau ^+$, and τ in this decreasing order of the last coordinate

In view of the estimate (20), it follows, for each of the simplices $\tau \in \tilde{\Sigma }(K)$ in Lemma 5.9, the cardinality of its respective subset $P^+_0(K;\tau )$ satisfies

$$ |P_0^+(K;\tau )|\ge |P_0(K)|/10^5\ge \max \{\varepsilon |P_0|/\vartheta ,d\}. $$

Let $\tau \in \tilde{\Sigma }(K)$, and consider the family $$\Pi _0^+(K;\tau ):={\Bigg (\begin{array}{c}P_0^+(K;\tau ) \\ d \end{array}\Bigg )}$$ of (d-1)-simplices over the set $P_0^+(K;\tau )$. Using that *K* is ε-spread within $\mathcal {V}(P_0,u)$ in a manner that is similar to case (b), it follows that the ϑ-canonical family $\mathcal {{L}}_0=\mathcal {{L}}\Big (P_0,u\Big )$ must include a line ℓ piercing at least *one* simplex $\pi \in \Pi _0^+(K;\tau )$. Furthermore, since any such simplex $\pi \in \Pi ^+_0(K;\tau )$ lies above the simplex $\phi ^+_\tau$ (whose supporting hyperplane $H\left( \phi ^+_\tau \right)$ lies above $\Phi '(\tau )\supset \tau$), this line ℓ must meet π, $\phi _\tau ^+$, and τ in this decreasing order of the *d*-th axis, and within the interval $K\cap \ell$, as depicted in Fig. 27.

We thus have found a subset $\tilde{\Sigma }(K)$ of at least $|\Sigma (K)|/10^4=\Theta \left( \left( \delta \epsilon n/s\right) ^d\right)$ simplices τ, and assigned each of them a simplex $\phi ^+_\tau \in {V'\atopwithdelims ()d}$ meeting some line $\ell \in \mathcal {{L}}_0$ at a point of $X'_\ell \cap K$. As any simplex $$\phi =(v_1,\ldots ,v_d)\in {\Bigg (\begin{array}{l}V' \\ d\end{array}\Bigg )}$$ has been assigned in this manner to at most $\left( \lfloor \epsilon _1n\rfloor \right) ^d$ simplices $\tau =(p_1,\ldots ,p_d)\in \tilde{\Sigma }(K)$, there must be such a line $\ell _0\in \mathcal {{L}}_0$ whose cross-section $\ell _0\cap K$ with *K* encompasses at least

$$ \frac{|\tilde{\Sigma }(K)|}{\left( \lfloor \epsilon _1 n \rfloor \right) ^d\cdot |\mathcal {{L}}_0|}=\Theta \left( \frac{(\delta \epsilon n/s)^d}{\left( \delta \epsilon n/\left( st^{1-1/\alpha _d}\right) \right) ^d\cdot |\mathcal {{L}}_0|}\right) =\Theta \left( \frac{t^{d-d/\alpha _d}}{u^{(d-1)^2}}\right) $$

points of $X'_{\ell _0}$. (Here the first bound uses the definition (15) of $\epsilon _1>0$ in Step 1.) Thus, provided a sufficiently small choice of $c_2>0$ in the definition of the nets $N'_{\ell }$, the interval $\ell _0\cap K$ must again contain a point of the net $N'_{\ell _0}\subset N_{>\delta ,2}$, which completes the analysis of the last scenario (c2).

In conclusion, any convex set $K\in {{\mathcal {K}}}_{>\delta }$ that fails to meet the criteria of flatness in Step 3, must fall into at least one of the four scenarios (a), (b), (c1), or (c2), that arise in our analysis, and, pending a suitably small choice of the constant $c_2>0$ in Step 2, be dispatched by the combination $N_{>\delta ,1}\cup N_{>\delta ,2}$ of their previous nets. □

### Proof of Lemma 5.6

The proof of Lemma 5.6 comes down to describing a sufficiently sparse subset $\tilde{\Pi }\subseteq {P\atopwithdelims ()d}$ that would still be “locally dense” over every principal subset $P_K$ that is cut out by some flat convex set $K\in {{\mathcal {K}}}_{>\delta }$.

**The subset** $\tilde{\Pi }=\tilde{\Pi }(P,\epsilon ,\delta ,s,t)$. To obtain such a subset $\tilde{\Pi }\subseteq {P\atopwithdelims ()d}$, which would not depend on the flat convex set $K\in {{\mathcal {K}}}_{>\delta }$ at hand, let us first consider the family

$$ \Lambda (P,s,t):=\biguplus _{P_{i,j}\in \mathcal {P}(P,s,t)} {P_{i,j}\atopwithdelims ()2}, $$

which is comprised of all such 2-edges in P2 whose two vertices come from the *same part* $P_{i,j}$ in our secondary partition $\mathcal {P}(P,s,t)$ of Step 1. Notice that

23 $$\begin{aligned} |\Lambda (P,s,t)|=O\left( \sum _{1\le i\le s}\sum _{1\le j\le t}{\lceil n/(st)\rceil \atopwithdelims ()2}\right) =O\left( \frac{n^2}{st}\right) . \end{aligned}$$

#### Definition

For every point p∈P, let us denote

$$\displaystyle \Pi _p:=\left\{ \pi \in {P\atopwithdelims ()d}\mid p\in \pi \right\} $$, and $$\displaystyle \Lambda _p:=\{\lambda \in \Lambda (P,s,t)\mid p\in \lambda \}$$.

Namely, the set $\Pi _p$ (resp., $\Lambda _p$) is comprised of all the (d-1)-dimensional simplices in Pd (resp., 2-edges in Λ(P)) that are adjacent to *p*. Note that every $\pi \in \Pi _p$ must be of the form ${{\texttt{conv}}}(p\cup \pi _p)$, where $\pi _p$ denotes some (d-2)-simplex in $$\left( \begin{array}{c}P\backslash \{p\}\\ d-1\end{array}\right) $$.

The subset $\tilde{\Pi }(P,\epsilon ,\delta ,s,t)\subseteq {P\atopwithdelims ()d}$ will be determined by the means of a delicate “local” relation between the simplices of $\Pi _p$ and the short 2-edges of $\Lambda _p$, that holds for each p∈P.

#### Definition

**Fig. 28:**
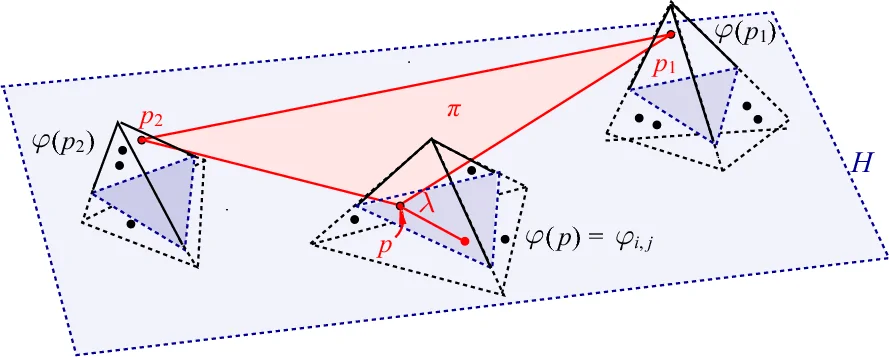
Proof of Lemma 5.6 in $\mathbb {R}^3$. The 2-edge $\lambda \in \Lambda _p$ and the triangle $\pi \in \Pi _p$ are friendly at the shared vertex *p* in $\mathbb {R}^3$. Depicted is the plane *H* through λ that intersects the ambient cells $\varphi (p_i)$ of the remaining 2 vertices of π

Let p∈P. We say that a short 2-edge $\lambda \in \Lambda _p$ and a (d-1)-simplex $\pi ={{\texttt{conv}}}(p\cup \pi _p)\in \Pi _p$ are *friendly* at their shared vertex *p* if there exists a hyperplane *H* through λ whose zone within the secondary partition $\mathcal {P}(P,s,t)$ encompasses the d-1 vertices $p_i$ of $\pi _p={{\texttt{conv}}}(p_1,\ldots ,p_{d-1})$; namely, *H* must contain both vertices of λ, and intersect the ambient cells $\varphi (p_i)$, for $1\le i\le d-1$ (which need not be distinct). See Fig. 28.

Let us now fix a sufficiently small constant $c_3>0$, which will be determined in the end of this proof (with implicit dependence on the constant $c_2>0$ in Step 2), and say that a simplex $\pi \in \Pi _p$ is *rich* at its vertex *p* if there exist at least

24 $$\begin{aligned} c_3\cdot \frac{\delta \epsilon n}{st^{1-1/d}}\gg \epsilon _2 n \end{aligned}$$

simplices $\lambda \in \Lambda _p$ that are friendly with π at *p*.

For each p∈P, let $\tilde{\Pi }_p\subseteq {P\atopwithdelims ()d}$ denote the subset of all such simplices $\pi \in {P\atopwithdelims ()d}$ that are rich at *p*. We then define

$$ \tilde{\Pi }(P,\epsilon ,\delta ,s,t):=\bigcup _{p\in P} \tilde{\Pi }_p. $$

It, therefore, remains to show that the just constructed subset $\tilde{\Pi }(P,\epsilon ,\delta ,s,t)\subseteq {P\atopwithdelims ()d}$ meets both criteria of Lemma 5.6 (pending a sufficiently small choice of the constant $c_3>0$ in (24)).

**Part (i) – bounding** $\left| \tilde{\Pi }(P,\epsilon ,\delta ,s,t)\right|$. To express the cardinality of $\tilde{\Pi }(P,\epsilon ,\delta ,s,t)$ in the terms of the much smaller quantity |Λ(P,s,t)|, we devise a *charging scheme* which assigns every $\pi \in \tilde{\Pi }(P,\epsilon ,\delta ,s,t)$ to multiple short 2-edges in Λ(P,s,t) that share a vertex with π.

For each point p∈P, we *assign* every simplex $\pi \in \tilde{\Pi }_p$ that is rich at *p*, to every short 2-edge $\lambda \in \Lambda _p$ that is friendly with π at *p*; each of these short 2-edges λ *pays* 1 unit of charge to π, via their shared vertex *p*. By definition, every $\pi \in \tilde{\Pi }(P,\epsilon ,\delta ,s,t)$ is rich at (at least) one of its *d* vertices $p\in \tau$ and, therefore, is assigned to at least $c_3\cdot \frac{\delta \epsilon n}{st^{1-1/d}}$

short 2-edges $\lambda \in \Lambda _p$. It, therefore, remains to bound the maximum number of simplices in $\tilde{\Pi }(P,\epsilon ,\delta ,s,t)$ that have been assigned in the above manner, to any given short 2-edge $\lambda \in \Lambda (P,s,t)$.

#### Lemma 5.10

Any short 2-edge $\lambda \in \Lambda (P,s,t)$ is assigned a total of $O\left( n^{d-1}/{t}^{1/d}\right)$ simplices of $\tilde{\Pi }(P,\epsilon ,\delta ,s,t)$.

#### Proof

Fix a short 2-edge $\lambda =\{p,q\}\in \Lambda (P,s,t)$. Note that, for every simplex $\pi ={{\texttt{conv}}}(p,p_1,\ldots ,p_{d-1})={{\texttt{conv}}}\left( p,\pi _p\right) \in \tilde{\Pi }(P,\epsilon ,\delta ,s,t)$, that has been assigned to λ via *p*, the remaining d-1 vertices $p_i$ of $\pi _p=(p_1,\ldots ,p_{d-1})$ lie the zone of some hyperplane *H* through λ. (In other words, *H* must contain both endpoints of λ, and also intersect the ambient cell $\varphi (p_i)$ of every vertex $p_i\in \pi _p$.)

Furthermore, according to Lemma 2.3, there must exist such a hyperplane *H* that passes through *p*, *q*, and some d-2 vertices $v_1,\ldots ,v_{d-2}$ of the (not necessarily distinct) cells $\varphi (p_i)$, with $1\le i\le d-1$. In other words, *H* is fully determined by the “signature” which is comprised of *p*, *q* and some d-2 vertices of the simplicial cells in the secondary partition $\mathcal {P}(P,s,t)$ of Step 2. It can, furthermore, be assumed, up to relabeling, that the vertices $v_1,\ldots ,v_{d-2}$ come entirely from the boundaries of the cells $\varphi (p_1),\ldots ,\varphi (p_{d-2})$. Hence, given *p*, *q*, and the first d-2 vertices $p_1,\ldots ,p_{d-2}$ of $\pi _p$, the hyperplane *H* can be “guessed” using Lemma 2.3 in up to $$O\Bigg (\left( \begin{array}{c}(d+1)(d-2) \\ d-2\end{array}\right) \Bigg )$$ distinct ways via the respective cells $\varphi (p_1),\ldots ,\varphi (p_{d-2})$.

Lastly, since any hyperplane meets only $O(st^{1-1/d})$ simplicial cells in $\mathcal {P}(P,s,t)$, fixing the points $p,q,p_1,\ldots ,p_{d-2}$, together with *H*, leaves only $O\left( \frac{n}{st}\cdot st^{1-1/d}\right) =O\left( n/t^{1/d}\right)$ possible choices for the remaining vertex $p_{d-1}$ from within the zone of *H*. Thus, the number of the possible choices of π cannot exceed $O\left( n^{d-2}\cdot n/t^{1/d}\right) =O\left( n^{d-1}/t^{1/d}\right)$. □

In view of the just established Lemma 5.10, and the upper bound (23) on the cardinality of Λ(P,s,t), the short 2-edges $\lambda \in \Lambda (P,s,t)$ transfer via their vertices a total of

$$ O\left( |\Lambda (P,s,t)|\cdot \frac{n^{d-1}}{t^{1/d}}\right) =O\left( \frac{n^{d+1}}{st^{1+1/d}}\right) $$

units of charge to the friendly rich simplices $\pi \in \tilde{\Pi }$ (each time via a common vertex *p* of λ and π, which may not be unique). Since any simplex of $\tilde{\Pi }$ receives at least $c_3\cdot \frac{\delta \epsilon n}{st^{1-1/d}}$ of these units of charge, via one or more of its vertices, it follows that

$$ \left| \tilde{\Pi }(P,\epsilon ,\delta ,s,t)\right| =O\left( \frac{n^{d+1}}{st^{1+1/d}}\cdot \frac{st^{1-1/d}}{\delta \epsilon n}\right) =O\left( \frac{n^d}{\delta \epsilon t^{2/d}}\right) , $$

which concludes the proof of the first part of Lemma 5.6.

**Part (ii).** It, therefore, remains to show, for any flat and δ-punctured convex set $K\in {{\mathcal {K}}}_{>\delta }$, that its induced set ${P_K\atopwithdelims ()d}$ encompasses sufficiently many simplices of the just defined family $\tilde{\Pi }=\tilde{\Pi }(P,\epsilon ,\delta ,s,t)\subseteq {P\atopwithdelims ()d}$.

To this end, recall that every set *K* as above is endowed with the principal (d+1)-tuple $\Psi (K)=(P_0,\ldots ,P_{d})$. Since *K* is flat, the secondary *t*-partition $\mathcal {P}_0=\mathcal {P}(P_0,t)$ of $P_0$ (or, more precisely, the partition $\mathcal {P}_0(K)$ of $P_0(K)$ by $\mathcal {P}_0$) yields the subset $\mathcal {T}_0(K)\subseteq \mathcal {P}_0(K)$, whose tight parts $P_{0,j}(K)$ together encompass at least $|P_0(K)|/5$ points of $P_0(K)$. Specifically, every part $P_{0,j}(K)\in \mathcal {T}_0(K)$ is full (that is, it satisfies $|P_{0,j}(K)|\ge \epsilon _2 n\ge 100$), and yields at least $|\Lambda _{0,j}(K)|\cdot |\Sigma (K)|/80$ tight pairs $(\lambda ,\tau )\in \Lambda _{0,j}(K)\times \Sigma (K)$.

At the heart of our argument lies the following property.

#### Lemma 5.11

Let $K\in {{\mathcal {K}}}_{>\delta }$ be a flat convex set, and $P_{0,j}(K)\in \mathcal {T}_0(K)$ a tight subset, and $(\lambda ,\tau )$ a tight pair in $\Lambda _{0,j}(K)\times \Sigma (K)$. Then there exists a hyperplane through λ that intersects at least d-1 among the ambient cells $\varphi (p_i)$ of the vertices of $\tau ={{\texttt{conv}}}(p_1,\ldots ,p_d)$.

#### Proof

**Fig. 29:**
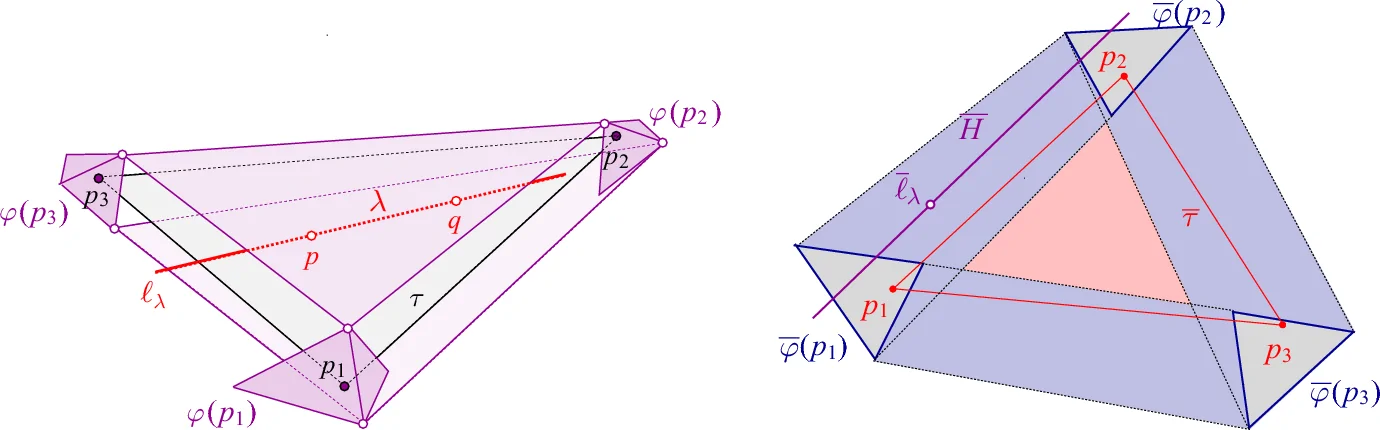
Lemma 5.11 in $\mathbb {R}^3$. Left: The pair $(\lambda ,\tau )$ is tight, so the line $\ell _\lambda$ through the 2-edge λ must intersect the convex hull of some 2 simplicial cells among $\varphi (p_1),\varphi (p_2),\varphi (p_3)$. Right: Proof of the lemma. Depicted are the projections $\overline{\varphi }(p_i)$ of the cells $\varphi (p_i)$ in the direction of $\ell _\lambda$, along with the intercept $\overline{\ell }_\lambda$ of $\ell _\lambda$. Since $\overline{\ell }_\lambda \not \in \overline{\tau }$, there exists a 1-flat (i.e., a line) $\overline{H}$ through $\overline{\ell }_\lambda$ that intersects some 2 among the projected sets $\overline{\varphi }(p_i)$

Refer to Fig. 29. Recall that, by the definition of a tight pair, both vertices of the short 2-edge $\lambda =\{p,q\}$ must lie in the envelope $\Phi (\tau )={{\texttt{conv}}}\left( \varphi (p_1)\cup \ldots \cup \varphi (p_d) \right)$ (which contains the *d* *distinct* simplicial cells $\varphi (p_i)$ within the respective secondary partitions $\mathcal {P}_i=\mathcal {P}(P_i,t)$, for $1\le i\le d$). Furthermore, τ is missed by the line $\ell _{\lambda }={{\texttt{aff}}}(\lambda )$. Projecting the cells $\varphi (p_i)$ in the direction of $\ell _\lambda$ yields *d* convex polytopes $\overline{\varphi }(p_i)$ within $\mathbb {R}^{d-1}$, while $\ell _\lambda$ similarly projects to a point $\overline{\ell }_\lambda \in \mathbb {R}^{d-1}$.

Note that $\overline{\ell }_\lambda$ cannot be surrounded by $\overline{\varphi }(p_1),\ldots ,\overline{\varphi }(p_d)$. Indeed, in the contrapositive scenario $\overline{\ell }$ would lie within the projection $\overline{\tau }$ of τ, whose *d* vertices are selected from the *d* distinct polytopes $\overline{\varphi }(p_i)$, in which case the line $\ell _\lambda$ would intersect τ.

Since $\overline{\ell }_\lambda$ clearly lies within the projection $\overline{\Phi }(\tau )={{\texttt{conv}}}\left( \overline{\varphi }(p_1)\cup \ldots \cup \overline{\varphi }(p_d)\right)$ of $\Phi (\tau )$ (as $\Phi (\tau )$ contains $\lambda \subset \ell _\lambda$), Lemma 2.2 yields a (d-2)-flat $\overline{H}$ within $\mathbb {R}^{d-1}$ that contains $\overline{\ell }_\lambda$, and intersects some d-1 among the *d* polytopes $\overline{\varphi }(p_i)$, say, $\overline{\varphi }(p_1),\ldots ,\overline{\varphi }(p_{d-1})$. (See Fig. 29 (right).) Then the respective d-1 cells $\varphi \left( p_1\right) ,\ldots ,\varphi \left( p_{d-1}\right)$ must be intersected in $\mathbb {R}^d$ by the hyperplane *H*, through λ, that is obtained by “lifting” $\overline{H}$ in the direction of $\ell _\lambda$. □

#### Definition

Let us fix a flat convex set $K\in {{\mathcal {K}}}_{>\delta }$. Denote $\tilde{P}(K):=\biguplus _{i=1}^d P_i(K)$. For every tight subset $P_{0,j}(K)\in \mathcal {T}_0(K)$, let $\Pi _{j}(K)$ denote the family $$P_{j}(K)*\left( \begin{array}{l}\tilde{P}(K) \\ d-2\end{array}\right) \subset {P_K\atopwithdelims ()d}$$ which is comprised of all such simplices that exactly one of their vertices belongs to $P_{0,j}(K)$, whereas the rest d-1 vertices are chosen from $\tilde{P}(K)$.

The asserted claim for *K* will follow from the fact that, provided a suitably small choice of $c_3>0$ in (24), every tight subset $P_{0,j}(K)$ within $\mathcal {T}_0(K)$ contributes to $\tilde{\Pi }\cap {P_K\atopwithdelims ()d}$ a subfamily $\tilde{\Pi }_j(K)$ of $\Theta \left( |\Pi _j(K)|\right) =\Theta \left( |P_{0,j}(K)|\cdot |\tilde{P}(K)|^{d-1}\right) \gg \epsilon _2n\cdot \left( \delta \epsilon n/s\right) ^{d-1}$ such simplices $\pi \in \Pi _j(K)$ that are rich at their only vertex within $P_{0,j}(K)$. The following lemma implies that an average simplex $\pi \in \Pi _j(K)$ is indeed friendly with $\Omega \left( |P_{0,j}(K)|\right) \gg \epsilon _2n$ short edges $\lambda \in \Lambda _{0,j}(K)$, at their shared vertex within $P_{0,j}(K)$.

#### Lemma 5.12

Let $K\in {{\mathcal {K}}}_{>\delta }$ be a tight convex set, and $P_{0,j}(K)\in \mathcal {T}_0(K)$ be a tight subset. Then there exist

$$ \Omega \left( |\Lambda _{0,j}(K)|\cdot |\tilde{P}(K)|^{d-1}\right) =\Omega \left( \left| P_{0,j}(K)\right| ^2\cdot \left( \delta \epsilon n/s\right) ^{d-1}\right) $$

friendly pairs $(\lambda ,\pi )$ with $\lambda \in \Lambda _{0,j}(K)$ and $\pi \in \Pi _{j}(K)$.

#### Proof

Since the subset $P_{0,j}(K)$ is tight, there exist at least $|\Lambda _{0,j}(K)\times \Sigma (K)|/80$ tight pairs $(\lambda ,\tau )\in \Lambda _{0,j}(K)\times \Sigma (K)$. For any short 2-edge $\lambda \in \Lambda _{0,j}(K)$, let $\Sigma _\lambda (K)$ denote the subset of all such simplices $\tau \in \Sigma (K)$ that yield a tight pair $(\lambda ,\tau )$. By the pigeonhole principle, $\Lambda _{0,j}(K)$ must contain a subset $\tilde{\Lambda }_{0,j}(K)$ of at least $\frac{1}{400}|\Lambda _{0,j}(K)|$ such short 2-edges λ that satisfy $|\Sigma _\lambda (K)|\ge \frac{1}{400}|\Sigma (K)|$.

We claim that any short 2-edge $\lambda ={{\texttt{conv}}}(p,q)\in \tilde{\Lambda }_{0,j}(K)$ must lie in a hyperplane $H_\lambda$ whose zone, within the secondary partition $\mathcal {P}(P,s,t)$, encompasses a subset $\tilde{P}_\lambda (K)\subseteq \tilde{P}(K)$ of cardinality $\Omega \left( |\tilde{P}(K)|\right)$. To see this, let $\tau \in \Sigma _\lambda (K)$. By the previous Lemma 5.11, there is a hyperplane *H* through λ that intersects at least d-1 ambient cells $\varphi (p_i)$ of the vertices of $\tau ={{\texttt{conv}}}(p_1,\ldots ,p_d)$. Furthermore, Lemma 2.3 yields such a hyperplane $H=H_{\lambda ,\tau }$ that, in addition, passes through *p*, *q*, and some d-2 vertices of the cells $\varphi (p_i)$. It thus can be assumed, with no loss of generality, that all of these vertices come from the boundaries of the first d-2 cells $\varphi \left( p_1\right) ,\ldots ,\varphi \left( p_{d-2}\right)$. Hence, the hyperplane *H* can be “guessed” from *p*, *q* and $p_1,\ldots ,p_{d-2}$ in $$O\bigg ({\left( \begin{array}{c}d(d-2) \\ d-2\end{array}\right) }\bigg )$$ ways.

We keep $\lambda \in \tilde{\Lambda }_{0,j}(K)$ fixed, and assign to each simplex $\tau \in \Sigma _\lambda (K)$ a unique signature $S_{\tau }=\{p_1,\ldots ,p_{d-2}\}$ in $$\left( \begin{array}{l}\tilde{P}(K) \\ d-2\end{array}\right) $$. The pigeonhole principle then yields such a signature $$S=\{p_1,\ldots ,p_{d-2}\}\in \left( \begin{array}{l}\tilde{P}(K) \\ d-2\end{array}\right) $$ that is shared by a subset $\Sigma _S\subseteq \Sigma _\lambda (K)$ of $\Theta \left( |\tilde{P}(K)|^2\right)$ simplices τ (which all satisfy $S_\tau =S$). Note that, for each of these simplices $\tau \in \Sigma _{S}$, at least one of the two “free” vertices in the pair $\tau \setminus S$ must fall in the zone of the hyperplane $H=H_{\lambda ,\tau }$.

Repeating this argument for all $\tau \in \Sigma _S$, and using the pigeonhole principle one more time, yields (i) a hyperplane *H* that contains λ along with some d-2 vertices chosen from the boundaries of $\varphi (p_1),\ldots ,\varphi (p_{d-2})$, and (ii) a subset $\tilde{P}_\lambda (K)$ of $\Omega \left( |\tilde{P}(K)|^2\right)$ such pairs in ${\tilde{P}(K)\atopwithdelims ()2}$ that at least one of their vertices lies in the zone *H*. As a result, the zone of *H* must contain a subset $\tilde{P}_\lambda (K)\subseteq \tilde{P}(K)$ whose cardinality is $\Theta \left( |\tilde{P}(K)|\right)$.

The asserted claim now stems from the following two facts: (i) any combination of $\lambda =\{p,q\}\in \tilde{\Lambda }_{0,j}(K)$ with a (d-1)-size subset $\mu \in {\tilde{P}_\lambda (K)\atopwithdelims ()d-1}$ yields two friendly pairs $(\lambda ,\tau )\in \Lambda _{0,j}(K)\times \Pi _j(K)$, with $\tau \in \{\mu \cup \{p\},\mu \cup \{q\}\}$, and (ii) a friendly pair $(\lambda ,\tau )$ is derived from at most *O*(1) combinations $(\lambda ,\mu )$, all of which involve the same set $\lambda \cup \tau$ of d+1 points. □

To complete the proof of Lemma 5.6, let us fix a tight subset $P_{0,j}(K)\in \mathcal {T}_0(K)$. For every simplex $\pi \in \Pi _{j}(K)$ let $\Lambda _{0,j}(K;\pi )$ denote the subset of all such simplices $\lambda \in \Lambda _{0,j}(K)$ that are friendly with π at its only vertex that belongs to $P_{0,j}(K)$. Since $\left| \Pi _{j}(K)\right| =\Theta \left( |P_{0,j}(K)|\cdot \left( \delta \epsilon n/s\right) ^{d-1}\right)$ and $|P_{0,j}(K)|\ge \epsilon _2 n=\Omega \left( \delta \epsilon n/\left( st^{1-1/d}\right) \right)$, Lemma 5.12 yields, via the pigeonhole principle, a subset $\tilde{\Pi }_{j}(K)\subset \Pi _{j}(K)$ of $\Theta \left( |P_{0,j}(K)|\cdot \left( \delta \epsilon n/s\right) ^{d-1}\right)$ such simplices $\pi \in \Pi _{j}(K)$ that satisfy $|\Lambda _{0,j}(K;\pi )|=\Theta \left( |P_{0,j}(K)|\right) =\Omega \left( \delta \epsilon n/\left( st^{1-1/d}\right) \right)$.

A sufficiently small choice of the constant $c_{3}>0$ in (24) guarantees that every $\pi \in \tilde{\Pi }_{j}(K)$ satisfies

$$ |\Lambda _{0,j}(K;\pi )|\ge c_{3}\cdot \frac{\delta \epsilon n}{st^{1-1/d}}; $$

that is, every $\pi \in \tilde{\Pi }_{j}(K)$ is rich at its only vertex within $P_{0,j}(K)$. As the subsets $\tilde{\Pi }_{j}(K)$ are clearly disjoint for all $1\le j\le t$, every tight subset $P_{0,j}(K)\in \mathcal {T}_0(K)$ contributes $|\tilde{\Pi }_{j}(K)|=\Theta \left( |\Pi _{j}(K)|\right)$ simplices to $\tilde{\Pi }\cap {P_K\atopwithdelims ()d}$, for a total amount of

$$\begin{aligned}&\sum _{P_{0,j}(K)\!\in \! \mathcal {T}_0(K)}|\tilde{\Pi }_{j}(K)|\gg \sum _{P_{0,j}(K)\in \mathcal {T}_0(K)}|\Pi _{j}(K)|\gg \\&\quad \gg \sum _{P_{0,j}(K)\in \mathcal {T}_0(K)}|P_{0,j}(K)|\cdot \left( \frac{\delta \epsilon n}{s}\right) ^{d-1} \gg |P_0(K)|\cdot \left( \frac{\delta \epsilon n}{s}\right) ^{d-1}=\Theta \left( \frac{\delta ^d\epsilon ^dn^d}{s^d}\right) . \end{aligned}$$

Here the third bound uses that $|\biguplus \mathcal {T}_0(K)|\ge |P_0(K)|/5$ for every flat convex set $K\in {{\mathcal {K}}}_{>\delta }$. This concludes the proof of Lemma 5.6. □

### Wrap Up for the δ-Punctured Sets

To finish the proof of Theorem 4.1, let us first point out that, according to Lemma 5.7, every ε-spread and δ-punctured set $K\in K_{>\delta }$ is pierced by the net $N_{>\delta }:=N_{>\delta ,1}\cup N_{>\delta ,2} \cup N_{>\delta ,3}$. Putting together the estimates in Lemmas 5.3, 5.4, and 5.7 yields

25 $$\begin{aligned} |N_{>\delta }|\le t^{1+\eta }\cdot f\left( \epsilon \cdot t^{1/\alpha _d-\eta }\right) +u^{1+\eta }\cdot f\left( \epsilon \cdot u^{1/(d-1)-\eta }\right) + \end{aligned}$$

$$ +O\left( t^{d/\alpha _d+\eta /(10d)}+\frac{t^{1-1/d}}{\epsilon ^{1+\eta }}+\frac{1}{\epsilon ^{d+1+\eta }t^{2/d}}\right) , $$

where $t=t_d$ is set to $\Big \lceil 1/\epsilon ^{\theta _d}\Big \rceil$, for some constant $d/2\le \theta _d\le \alpha _d$ that is left to be determined for all d≥3.

Since all the recursive terms in (25) are of the “right” sort $a^{1+\eta }\cdot f\left( \epsilon \cdot b^{1-\eta }\right)$, with $b\ge a^{\frac{1}{\alpha _d}}$, it suffices to fix such an exponent $d/2\le \theta _d\le \alpha _d$ that would render all the three non-recursive terms $O\left( (1/\epsilon )^{\alpha _d+\eta }\right)$.

For d=3, fixing $\theta _3:={2.165}$ renders all the non-recursive terms $O\left( 1/\epsilon ^{2.558+\eta }\right) =O\left( 1/\epsilon ^{\alpha _d+\eta }\right)$.

For d=4, we use $\theta _4:=3.02$, and similarly check that all the non-recursive terms are $O\left( (1/\epsilon )^{3.48+\eta }\right)$.

In any higher dimension d≥5, we choose $\theta _d:=d(d+1-\alpha _d)/2=d(3/4+o_d(1))$ so as to directly render the third non-recursive term $O\left( (1/\epsilon )^{\alpha _d+\eta }\right)$. It suffices to check the first two non-recursive terms are $O\left( 1/\epsilon ^{\alpha _d+\eta }\right)$.

Indeed, as we have that

$$ \theta _d=d(d+1-\alpha _d)/2=\frac{d}{4}\left( d+2-\sqrt{d^2-2d}\right) $$

and, in particular, $\theta _d<d-1$ for all d≥5, the second non-recursive term is $O\left( (1/\epsilon )^{\frac{(d-1)^2}{d}+1+\eta }\right) =O\left( (1/\epsilon )^{d-1+1/d+\eta }\right) =O\left( (1/\epsilon )^{\alpha _d+\eta }\right)$ for all d≥5.

Lastly, the first non-recursive term satisfies

$$ t^{d/\alpha _d+\eta /(10d)}=O\left( (1/\epsilon )^{\frac{d^2\cdot \left( d+2-\sqrt{d^2-2d}\right) }{2d+2\sqrt{d^2-2d}}+\eta }\right) , $$

where the exponent in the right hand side is smaller than $\alpha _d+\eta =\frac{1}{2}(d+\sqrt{d^2-2d})+\eta$ for all d≥5. □

## Piercing the δ-Hollow Sets – Proof of Theorem 4.2

**Setup.** Throughout this section, we will stick with the quantities *P*, Π, ϵ, σ, n=|P|, and the auxiliary parameters that were set in the beginning of Sect. 4. In particular, we will use positive integers *h*, *r*, *s*, and *u*, that are very small, albeit fixed and positive, powers of 1/ϵ, which satisfy

$$ h\lll _\eta r\lll _\eta s\lll _\eta u\lll _\eta 1/\epsilon , $$

the fraction $\delta =1/r^{d(d+1)+1}$, and the “spread threshold” $\varepsilon =\Omega (\epsilon ^{1+\eta })$ whose value (7) has been selected in the beginning of Sect. 4.

In the rest of this section, we denote m:=|Π|, and use ${\mathcal {H}}$ to denote the family ${\mathcal {H}}(\Pi )=\{H(\tau )\mid \tau \in \Pi \}$ of the *m* hyperplanes that support the simplices of Π. Recall that we have that $\epsilon {n\atopwithdelims ()d}\le |\Pi |\le \rho {n\atopwithdelims ()d}$, and that our “edge-constrained” family ${{\mathcal {K}}}={{\mathcal {K}}}(P,\Pi ,\epsilon ,\sigma )$ consists of all such convex sets $K\in {{\mathcal {K}}}(P,\epsilon )$ that are $(\epsilon ,\sigma )$-restricted to the hypergraph (P,Π) and, thereby, satisfy $|\Pi _K|\ge \sigma {|P_K|\atopwithdelims ()d}$. (As before, $\Pi _K$ is used to denote the subset ${P_K\atopwithdelims ()d}\cap \Pi$ of Π that is induced by the points of $P_K$.) Also recall that the fraction $0<\sigma \le 1$ is bounded from below by some constant $\sigma _0>0$ that was described in Sect. 4.

To establish Theorem 4.2 in Sect. 4 and, therefore, complete the proof of Theorem 1.1, we will describe a small-size net $N_{\le \delta }$ for the sub-family ${{\mathcal {K}}}_{\le \delta }$, which is comprised of all such δ-hollow convex sets in ${{\mathcal {K}}}$ that are ε-spread within the vertical partition $\mathcal {V}(P,s)$ of *P*, which too was introduced in Sect. 4.

Similar to Sect. 5, our divide-and-conquer treatment of the family ${{\mathcal {K}}}_{\le \delta }$ will use a decomposition of the ground point set *P*, along with the ambient space $\mathbb {R}^d$. However, for reasons that will become clear towards the end of this section, this decomposition will be based on the random arrangement $\mathcal {A}(\mathcal {R})$ of an *r*-sample $\mathcal {R}\subseteq {\mathcal {H}}={\mathcal {H}}(\Pi )$, and a subsequent refinement of $\mathcal {A}(\mathcal {R})$ to an $O\left( \frac{\log r}{r}\right)$-cutting $\mathcal {D}$ [21, 25] of the family ${\mathcal {H}}={\mathcal {H}}(\Pi )$.

**Fig. 30:**
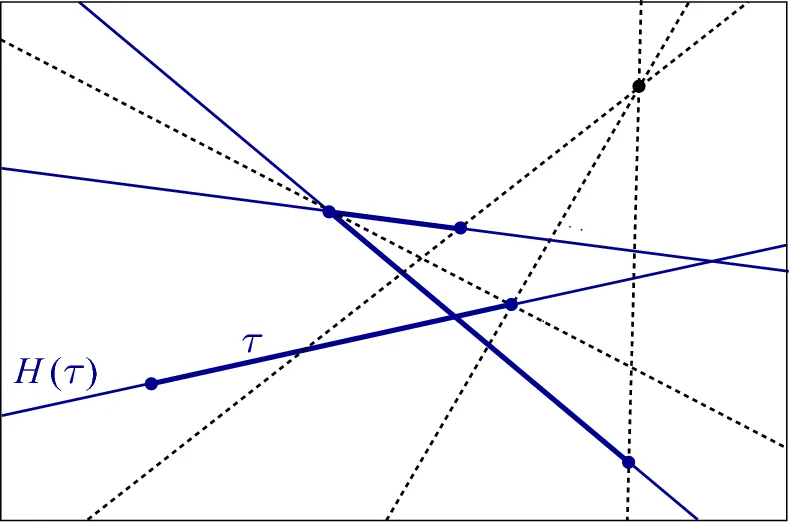
The random arrangement $\mathcal {A}(\mathcal {R})$ illustrated in $\mathbb {R}^2$. The family ${\mathcal {H}}={\mathcal {H}}(\Pi )$ is comprised of the hyperplanes H(τ) that support the simplices of $\tau \in \Pi$. The hyperplanes of the *r*-sample $\mathcal {R}\subset {\mathcal {H}}(\Pi )$ are blue and solid, whereas the rest of the hyperplanes of ${\mathcal {H}}(\Pi )$ are black and dashed

**The random arrangement** $\mathcal {A}(\mathcal {R})$. Combining the assumption $m\ge \epsilon {n\atopwithdelims ()d}$ with the lower bound (8) on *n*, we obtain that $|{\mathcal {H}}|=m\ge r$. Consider a random *r*-sample $\mathcal {R}$ of ${\mathcal {H}}$. Note that the hyperplanes of $\mathcal {R}$ are not necessarily in general position (because those of ${\mathcal {H}}$ are, most likely, *not* – any point of *P* can lie in up to nd-1 hyperplanes of ${\mathcal {H}}$). Nevetheless, any hyperplane in ${\mathcal {H}}$ contains exactly *d* points of *P*. As was mentioned in Sect. 2.5, the hyperplane collection $\mathcal {R}$ determines the *arrangement* $\mathcal {A}(\mathcal {R})$ whose *d*-dimensional faces constitute a decomposition of $\mathbb {R}^d\setminus (\bigcup R)$ into $O\left( r^d\right)$ open polyhedral *cells*, of overall complexity $O(r^d)$ [43, Section 6]. See Fig. 30.

As a result, the ground point set *P* is subdivided into two parts $P^{\prime }$ and $P^{\prime \prime }$. The larger part $$P'=P\setminus \left( \bigcup \mathcal {R}\right) $$ is comprised of all the points in *P* that lie in the interiors of the cells of $$\mathcal {A}(\mathcal {R})$$, whereas the complementary subset $P^{\prime \prime }$ consists of at most *rd* points that lie on one or more of the sample hyperplanes of $$\mathcal {R}$$. By again using the lower bound (8) on *n*, it follows that the principal subset $$P_K$$ of every convex set $$K\in {{\mathcal {K}}}_{\le \delta }$$, must encompass at least $$\lceil \epsilon n\rceil -rd\ge \lceil \epsilon n\rceil /2$$ points of $P^{\prime }$.

**Fig. 31:**
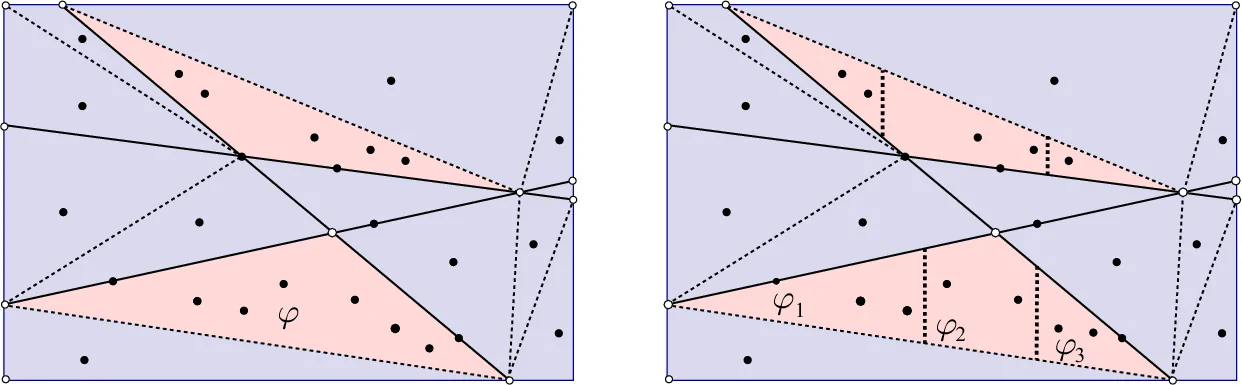
The cutting $\mathcal {D}(\mathcal {R},P)$ illustrated in $\mathbb {R}^2$. We begin with a bottom-vertex triangulation $\mathcal {D}(\mathcal {R})$ of the cells of $\mathcal {A}(\mathcal {R})$ (left). The “overfull” cells $\varphi \in \mathcal {D}(\mathcal {R})$, with at least $\lceil n/k\rceil$ points in their interiors, are called *red*; each of them is split, by the means of vertical walls, into several red cells $\varphi _i$ within $\mathcal {D}=\mathcal {D}(\mathcal {R},P)$ (right)

**The cutting** $$\mathcal {D}=\mathcal {D}(\mathcal {R},P)$$. Applying the bottom-vertex triangulation of Sect. 2.5 to the cells of $$\mathcal {A}(\mathcal {R})$$ yields a simplicial decomposition $$\mathcal {D}(\mathcal {R})$$, that is, a collection of $$O\left( r^d\right) $$ pairwise disjoint open *d*-dimensional simplices, so that every point of $P^{\prime }$ lies in the interior of a unique simplicial cell φ of $$\mathcal {D}(\mathcal {R})$$. See Fig. 31 (left). As has been noted in Sect. 2.5, we have that, with probability at least 1/2, the interior of such cell φ of $$\mathcal {D}(\mathcal {R})$$ is crossed by $$O\left( \frac{m}{r}\log r\right) $$ hyperplanes of $${\mathcal {H}}$$. Hence, in the broadly accepted terminology [21, 23, 25], the decomposition $$\mathcal {D}(\mathcal {R})$$ already yields a O(logr/r)-cutting of $${\mathcal {H}}$$.

To further refine the described cutting $\mathcal {D}(\mathcal {R})$, let us fix another auxiliary parameter $k:=r^{(d+\sqrt{d^2-2d})/2}\le r^{\alpha _d}$, which satisfies $r^{d-1}\le k\le r^{d-1/2}$.

### Definition

Let us denote $P_\varphi :=P\cap \varphi$ for each cell $\varphi \in \mathcal {D}(\mathcal {R})$. We say that a simplicial cell $\varphi \in \mathcal {D}(\mathcal {R})$ is *red* if $|P_\varphi |\ge \lceil n/k\rceil$, and we say that it is *blue* otherwise. By definition, there exist at most *k* red cells in $\mathcal {D}(\mathcal {R})$.

We further subdivide every red cell φ of $$\mathcal {D}(\mathcal {R})$$ into $$\lceil |P_\varphi | /\lfloor \frac{n}{k}\rfloor \rceil $$ open polyhedral cells $\varphi^{\prime }$, which satisfy $$|\varphi '\cap P|\le \lfloor n/k\rfloor $$. To this end, we introduce at most $$\lceil |P_\varphi | /\lfloor \frac{n}{k}\rfloor \rceil -1\le 2|P_\varphi | \frac{k}{n}-1$$ walls which are orthogonal to the first coordinate axis. (None of these walls can pass through a point of *P*.) Let $$\mathcal {D}(\mathcal {R},P)$$ denote this more refined decomposition of $$\mathbb {R}^d$$, as illustrated in Fig. 31 (right).

### Definition

For every red simplex of $\mathcal {D}(\mathcal {R})$ we refer to *all* its off-shoots in $\mathcal {D}(\mathcal {R},P)$ as *red*. If a cell of $\mathcal {D}(\mathcal {R},P)$ is not red, we say that it is *blue*, in which case it has been inherited en toto from the bottom-vertex triangulation $\mathcal {D}(\mathcal {R})$.

Note that the refined decomposition $\mathcal {D}:=\mathcal {D}(\mathcal {R},P)$ still encompasses $O\left( r^d\right)$ cells, including at most 2*k* red cells; each of these cells φ is a convex polytope with at most d+2 facets, at most ${d+1\atopwithdelims ()2}+2{d\atopwithdelims ()2}=O(d^2)$ edges, and $O(d^2)$ vertices. As $\mathcal {D}$ is a refinement of the $O\left( \frac{1}{r}\log r\right)$-cutting $\mathcal {D}(\mathcal {R})$, any open cell of $\mathcal {D}$ is still crossed by $O\left( \frac{m}{r}\log r\right)$ hyperplanes of ${\mathcal {H}}$, which yields the following immediate corollary.

### Observation 6.1

There is a constant $c_{ cut}>0$, which depends only on the dimension *d*, so that the interior of every cell $\varphi \in \mathcal {D}(\mathcal {R},P)$ is crossed by at most $c_{ cut}\left( \frac{m}{r}\log r\right)$ hyperplanes of ${\mathcal {H}}$.

The net $N_{\le \delta }$ will combine the following key ingredients: (i) $\tilde{\epsilon }$-nets which are defined over smaller ground sets $\tilde{P}$, each of them cut out by either a cell of $\mathcal {A}(\mathcal {R})$, or a red cell φ of $\mathcal {D}$, (ii) 1-dimensional $\Omega (\epsilon ^{d-1+\eta })$-nets which will be constructed within the lines of the canonical family $\mathcal {{L}}=\mathcal {{L}}(P,s)$, that was defined in Sect. 4 over the vertical partition $\mathcal {V}(P,s)$.

Similar to its predecessor $N_{>\delta }$ in Sect. 5, the net $N_{\le \delta }$ will be defined in three incremental steps. Initially, the set $N_{\le \delta }$ is empty. In each subsequent step $1\le i\le 3$ we add to $N_{\le \delta }$ a relatively simple partial net $N_{\le \delta ,i}$ that pierces one or several specific categories of the convex sets, to be immediately removed from ${{\mathcal {K}}}_{\le \delta }$. By the end of Step 3, no set in ${{\mathcal {K}}}_{\le \delta }$ will be left unpierced by $N_{\le \delta }=\bigcup _{i=1}^3N_{\le \delta ,i}$.

The first two nets $N_{\le \delta ,1}$ and $N_{\le \delta ,2}$ will overly resemble their counterparts in Sect. 5, and make no use of the δ-hollowness of the sets $K\in {{\mathcal {K}}}_{\le \delta }$.

By the end of Step 2, it will be shown that every remaining convex set $K\in {{\mathcal {K}}}_{\le \delta }$, that have been “missed” by the combination $N_{\le \delta ,1}\cup N_{\le \delta ,2}$, is sufficiently “thin” with respect to both the random arrangement $\mathcal {A}(\mathcal {R})$, and its finer cutting $\mathcal {D}=\mathcal {D}(\mathcal {R},P)$. In particular, a large fraction of its principal subset $P_K$ will lie in the zone of a certain *principal hyperplane* $H_K\in {\mathcal {H}}$, which crosses only $O^*\left( \frac{k}{r}\right)$ among the red cells of $\mathcal {D}(\mathcal {R},P)$.

In Step 3, the δ-hollowness will be used, at last, to dispose of the remaining convex sets $K\in {{\mathcal {K}}}_{\le \delta }$ by the means of the basic recurrence in ϵ>0, that was sketched in the beginning of Sect. 2.6.

### Step 1

#### Definition

Let $\Pi^{\prime \prime }$ denote the subset of all the simplices $$\tau \in \Pi $$ that meet at least one of the following criteria:

(i) one or more of the vertices of τ belong to $P^{\prime \prime }$ and, therefore, lie on a hyperplane of $$\mathcal {R}$$.
(ii) the supporting hyperplane $H(\tau )\in {\mathcal {H}}$ crosses at least $4c_{ cut}\cdot \frac{hk}{r}\log r$ red cells of $\mathcal {D}=\mathcal {D}(\mathcal {R},P)$.

We then denote $\Pi ':=\Pi \setminus \Pi ''$.

**The net** $N_{\le \delta ,1}$. We set

26 $$\begin{aligned} N_{\le \delta ,1}:=N(P,\Pi '',\epsilon ,\sigma /2), \end{aligned}$$

where the right hand side denotes the net of cardinality at most $$f(\epsilon ,|\Pi ''|/{n\atopwithdelims ()d},\sigma /2)$$, that exists for the family $${{\mathcal {K}}}(P,\Pi '',\epsilon ,\sigma /2)$$, which is comprised of all such convex sets $$K\in {{\mathcal {K}}}(P,\epsilon )$$ that are $$(\epsilon ,\sigma /2)$$-restricted to the hypergraph $\left(P,\Pi^{\prime \prime }\right)$ (and thus satisfy $$|{P_K\atopwithdelims ()d}\cap \Pi ''|\ge (\sigma /2){|P_K|\atopwithdelims ()d}$$).

**The analysis.** The following claim shows that the recursive instance (26) involves a considerably sparser subset $\Pi^{\prime \prime }$.

#### Lemma 6.2

We have that $|\Pi ''|\le m/h\le \rho {n\atopwithdelims ()d}/h$.

#### Proof

Using the lower bound (8) on n=|P| in the beginning of Sect. 4, that $$h\lll _\eta r\lll _\eta 1/\epsilon $$, and that $$m=|\Pi |\ge \epsilon {n\atopwithdelims ()d}$$, it follows that at most $$rd {n \atopwithdelims ()d-1}\le r {n\atopwithdelims ()d}/n\le rm/(\epsilon n)\le m/(2h)\le \rho {n\atopwithdelims ()d}/(2h)$$ simplices of $\Pi^{\prime \prime }$ can fall in the first category.

Furthermore, in view of Observation 6.1, there exist at most $2c_{ cut}\cdot \frac{mk}{r}\log r$ “incidences” between the (at most 2*k*) red cells of $\mathcal {D}$, and the *m* hyperplanes of ${\mathcal {H}}$ that intersect their interiors. Thus, by the pigeonole principle, at most $m/(2h)\le \rho {n\atopwithdelims ()d}/(2h)$ simplices $\tau \in \Pi$ can fall in the second category. □

The properties of $N_{\le \delta ,1}$ are summarized in the following lemma.

#### Lemma 6.3

1. The net $N_{\le \delta ,1}$ has cardinality at most $f(\epsilon ,\rho /h,\sigma /2)$.
2. Upon including the points of $N_{\le \delta ,1}$ in $N_{\le \delta }$ and, accordingly removing from ${{\mathcal {K}}}_{\le \delta }$ all the sets that are pierced by $N_{\le \delta ,1}$, every remaining set $K\in {{\mathcal {K}}}_{\le \delta }$ satisfies $|{P_K\atopwithdelims ()d}\cap \Pi '|\ge (\sigma /2){\lceil \epsilon n\rceil \atopwithdelims ()d}$. Namely, at least $\Big \lceil (\sigma /2) {\lceil \epsilon n\rceil \atopwithdelims ()d}\Big \rceil$ of the simplices in the family $\Pi _K={P_K\atopwithdelims ()d}\cap \Pi$ belong to P′d, and their supporting hyperplanes H(τ) in ${\mathcal {H}}$ cross the interiors of fewer than $4c_{ cut}\cdot \frac{hk}{r}\log r$ red cells within $\mathcal {D}$.

In the sequel, we denote $P'_K:=P_K\cap P'$, and $\Pi '_K:=\Pi '\cap {P_K\atopwithdelims ()d}$, for every $K\in {{\mathcal {K}}}_{\le \delta }$.

### Step 2

Our second net $N_{\le \delta ,2}$ for the family ${{\mathcal {K}}}_{\le \delta }$ will be comprised of 1-dimensional $\Omega \left( \epsilon ^{d-1+\eta }\right)$-nets $N_\ell$, which will be restricted to the $O\left( s^{(d-1)^2}\right)$ vertical lines ℓ of the ϑ-canonical family $\mathcal {{L}}(P,s)$ over the vertical partition $\mathcal {V}(P,s)$.

Upon adding the points of $N_{\le \delta ,2}$ to $N_{\le \delta }$, and thereby removing from ${{\mathcal {K}}}_{\le \delta }$, every convex set that is pierced by them, every remaining convex set $K\in {{\mathcal {K}}}_{\le \delta }$ will be assigned a so called *principal hyperplane* $H_K\in {\mathcal {H}}\left( \Pi '_K\right)$, whose zone in $\mathcal {D}$ will contain $\Omega (\epsilon n)$ points of $P_K$.

**The net** $N_{\le \delta ,2}$. The net $N_{\le \delta ,2}$ is attained through several explicit constructions using the ϑ-canonical line set $\mathcal {{L}}(P,s)$, the sample $\mathcal {R}\subseteq \Pi$ of *r* hyperplanes, and the cutting $\mathcal {D}=\mathcal {D}(\mathcal {R},P)$, which has been defined in the beginning of this section.

1. For each vertical line $\ell \in \mathcal {{L}}(P,s)$, we consider the set
  $$ X_\ell :=\{\ell \cap H\mid H\in \mathcal {R}\} $$
  of all the ℓ-intercepts of the hyperplanes in $\mathcal {R}$, and let
  $$ X:=\bigcup _{\ell \in \mathcal {{L}}(P,s)} X_\ell . $$
  We include in the latter set *X* every vertex of $\mathcal {D}$. Since $r\lll _\eta s$, we have that
  $$ |X|\le s^{(d-1)^2}r+O(r^d)=O\left( s^{(d-1)^2}r\right) . $$
2. We then use the points of *X* to define a smaller auxiliary family $\Pi _X$ of “synthetic” (d-1)-simplices
  $$ \Pi _X:={P\atopwithdelims ()d-1}*X=\left\{ {{\texttt{conv}}}(\kappa \cup \{x\})\mid \kappa \in {P\atopwithdelims ()d-1},x\in X\right\} $$
  which determine the family ${\mathcal {H}}_X:={\mathcal {H}}(\Pi _X)$ of supporting hyperplanes. Notice that
  $$ |{\mathcal {H}}_X|=|\Pi _X|=O\left( n^{d-1}|X|\right) =O\left( n^{d-1}s^{(d-1)^2}r\right) . $$
3. For each canonical line $\ell \in \mathcal {{L}}(P,s)$ we construct the point set
  $$ Y_\ell =:\{\ell \cap H\mid H\in {\mathcal {H}}_X\} $$
  which encompasses all the ℓ-intercepts of the hyperplanes of ${\mathcal {H}}_X$, and note that $|Y_\ell |=O\left( n^{d-1}s^{(d-1)^2}r\right)$.
4. Lastly, for each line $\ell \in \mathcal {{L}}(P,s)$ we construct the net $N_\ell$ which includes every $\Big \lceil (\vartheta /4)(\sigma _0/2)^{\beta _{d-1}}{\lceil \epsilon n\rceil \atopwithdelims ()d-1}\Big \rceil$-th point of $Y_\ell$, and let
  $$ N_{\le \delta ,2}:=\bigcup _{\ell \in \mathcal {{L}}(P,s)}N_\ell . $$

We add $N_{\le \delta ,2}$ to $N_{\le \delta }$, and remove from $K_{\le \delta }$ every convex set already pierced by $N_{\le \delta ,2}$.

**The analysis.** Using that $r\lll _\eta s\lll _\eta 1/\epsilon$ (and that $\sigma _0$ is a constant that depends only on η and the dimension *d*), we obtain the following immediate bound on the cardinality of $N_{\le \delta ,2}$.

#### Lemma 6.4

The previously defined net $N_{\le \delta ,2}$ has cardinality

$$ O\left( \frac{s^{2(d-1)^2}r}{\sigma _0^{\beta _{d-1}}\epsilon ^{d-1}}\right) =O\left( \frac{1}{\epsilon ^{d-1+\eta }}\right) . $$

#### Definition

For any point $p\in \mathbb {R}^d$, and any non-vertical hyperplane *H* in $\mathbb {R}^d$, we use $[p]_H$ to denote the vertical projection of *p* onto *H*, and we use *e*(*p*, *H*) to denote the closed vertical segment between *p* and $[p]_H$.

**Fig. 32:**
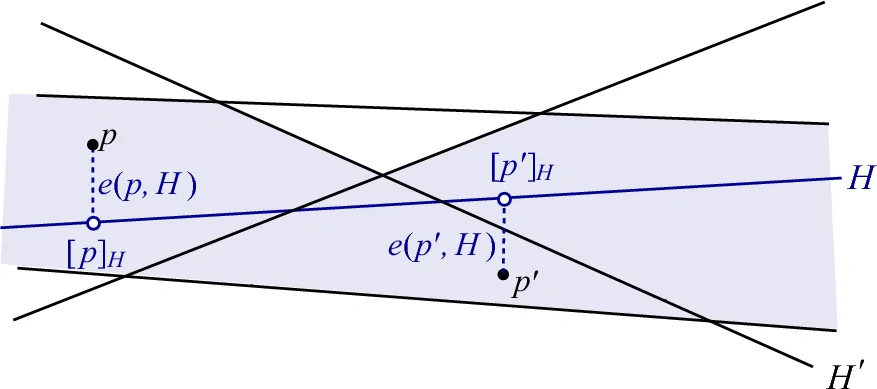
The point $$p\in P_K$$ *sees* the hyperplane $$H\in {\mathcal {H}}$$ in $$\mathcal {A}(\mathcal {R})$$: its *H*-projection $$[p]_H$$ lies in the same cell of $$\mathcal {A}(\mathcal {R})$$. In contrast, the point $p^{\prime }$ *does not see* *H* in $$\mathcal {A}(\mathcal {R})$$, as the vertical segment $e(p^{\prime },H)$ between $p^{\prime }$ and $$[p']_H$$ is crossed by a hyperplane $$H'\in \mathcal {R}$$. Notice that both *p* and $p^{\prime }$ lie in the shaded zone of *H* in $$\mathcal {A}(\mathcal {R})$$

We then say that *p* *sees* the hyperplane *H* in $\mathcal {A}(\mathcal {R})$ if *e*(*p*, *H*) does not cross any hyperplane in $\mathcal {R}$. (In other words, both *p* and $p_H$ have to lie in the same open cell of $\mathcal {A}(\mathcal {R})$. See Fig. 32.)

#### Lemma 6.5

Let

$$ c'_2:=\frac{d\vartheta }{2^{\beta _{d-1}+5}}. $$

Then every convex set $K\in {{\mathcal {K}}}_{\le \delta }$ that is missed by $N_{\le \delta ,1}\cup N_{\le \delta ,2}$, can be assigned a hyperplane $H_K\in {\mathcal {H}}\left( \Pi '_K\right)$, and a subset $A_K\subseteq P_K$, with the following properties:

(i) We have that $|A_K|\ge c'_2 \sigma _0^{\beta _{d-1}} \lceil \epsilon n \rceil$.
(ii) $A_K$ is contained in the zone of $H_K$ in $\mathcal {D}(\mathcal {R},P)$.
(iii) Every point $p\in A_K$ sees $H_K$; see Fig. 32.

#### Definition

We assign to each remaining convex set $K\in {{\mathcal {K}}}_{\le \delta }$ a *unique* hyperplane $H_K\in {\mathcal {H}}\left( \Pi '_K\right)$ with a *unique* subset $A_K\subseteq P_K$, that together meet the criteria (i) – (iii) of Lemma 6.5, and refer to $H_K$ as the *principal hyperplane* of *K*.

#### Proof of Lemma 6.5

We fix a convex set $K\in {{\mathcal {K}}}_{\le \delta }$, and suppose, for a contradiction, that there does not exist any such combination of a hyperplane $H_K\in {\mathcal {H}}\left( \Pi '_K\right)$, with a subset $A_K\subseteq P_K$, that together meet the criteria (i) – (iii). We show that *K* is already pierced by the net $N_{\le \delta ,2}$.

Indeed, since (a) *K* is ε-spread in $\mathcal {V}(P,s)$, with the the threshold ε>0 in (7) which is much smaller than $\vartheta (\sigma _0/2)^{\beta _{d-1}}\epsilon$, and (b) we have that $|\Pi '_K|\ge \frac{\sigma }{2} {\lceil \epsilon n\rceil \atopwithdelims ()d}\ge \frac{\sigma _0}{2} {\lceil \epsilon n\rceil \atopwithdelims ()d}$ (by Lemma 6.3), the ϑ-canonical family $\mathcal {{L}}(P,s)$ must include a line $\ell _0\in \mathcal {{L}}(P,s)$ that pierces a subset $\Pi '_K(\ell _0)$ of $\vartheta \left( \frac{\sigma _0}{2}\right) ^{\beta _{d-1}}{\lceil \epsilon n\rceil \atopwithdelims ()d}$ simplices within $\Pi '_K$.

To proceed with the proof of Lemma 6.5, let us introduce further notation.

#### Definition

Let κ be a (d-2)-simplex in ${P'_K\atopwithdelims ()d-1}$.

**Fig. 33:**
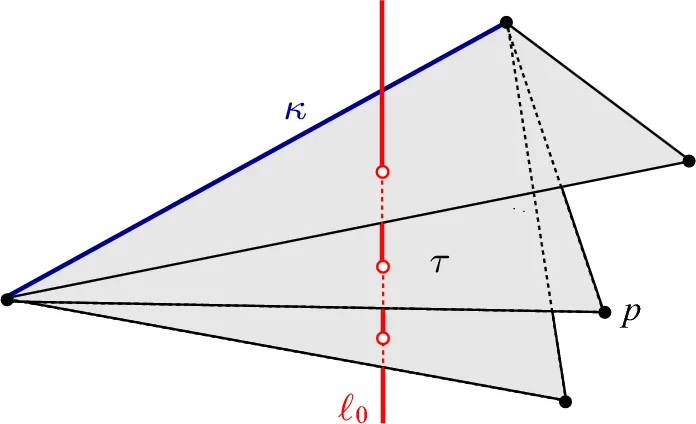
A (d-2)-simplex κ along with the adjacent simplices $\tau \in \Pi '_K(\ell _0)$, which are crossed by the canonical line $\ell _0$. Each $\tau \in \Pi '_K(\ell _0)$ contributes a vertex *p* to the neighbor set $\Gamma '_K(\kappa )$

1. We say that a simplex $\tau \in \Pi '_K(\ell _0)$ is *adjacent* to κ if κ contains d-1 vertices of τ. We then say that a point $p\in P'_K$ is a *neighbor* of κ if the (d-1)-dimensional simplex ${{\texttt{conv}}}(\kappa \cup \{p\})$ belongs to $\Pi '_K(\ell _0)$. See Fig. 33.
2. We use $\Gamma '_K(\kappa )\subseteq P_K$ to denote the subset of all the neighbours of κ. (Notice that the points of $\Gamma '_K(\kappa )$ correspond to the simplices of $\Pi '_K(\ell _0)$ that are adjacent to κ.)
3. We say that κ is *good* if we have that
  27 $$\begin{aligned} |\Gamma '_K(\kappa )|\ge \frac{\vartheta }{4d}\cdot \left( \frac{\sigma _0}{2}\right) ^{\beta _{d-1}}\left( \lceil \epsilon n\rceil -d+1\right) \ge \frac{\vartheta }{8d}\cdot \left( \frac{\sigma _0}{2}\right) ^{\beta _{d-1}}\lceil \epsilon n\rceil , \end{aligned}$$
  where the last inequality follows from the lower bound (8) on *n*.

Since $|\Pi '_K(\ell _0)|\ge \vartheta \left( \frac{\sigma _0}{2}\right) ^{\beta _{d-1}}{\lceil \epsilon n\rceil \atopwithdelims ()d}$, the pigeonhole principle yields the following property.

#### Proposition 6.6

There exist at least $\frac{\vartheta }{4}\left( \frac{\sigma _0}{2}\right) ^{\beta _{d-1}}{\lceil \epsilon n \rceil \atopwithdelims ()d-1}$ good (d-2)-simplices in ${P'_K\atopwithdelims ()d-1}$.

#### Definition

We say that a good (d-2)-simplex $\kappa \in {P'_K\atopwithdelims ()d-1}$ is *very good* if for any two simplices $\tau _1,\tau _2\in \Pi '_K(\ell _0)$ that are adjacent to κ, their supporting hyperplanes $H_1:=H(\tau _1)$ and $H_2:=H(\tau _2)$ determine *identical* zones in $\mathcal {D}$ (i.e., $H_1$ and $H_2$ cross the *same* cells of $\mathcal {D}$).

To establish the lemma, it is enough to show that every good (d-2)-simplex $\kappa \in {P_K\atopwithdelims ()d-1}$ gives rise to a “synthetic” simplex $\pi _\kappa \in \Pi _X$, of the general form ${{\texttt{conv}}}(\kappa \cup \{x\})$; the supporting hyperplane $H\left( \pi _\kappa \right)$ then meets $\ell _0$ at a point of $Y_{\ell _0}\cap K$. Repeating this argument for each good (d-2)-simplex $\kappa \in {P'_K\atopwithdelims ()d-1}$ would yield, according to Proposition 6.6, a total of at least $\frac{\vartheta }{8}\left( \frac{\sigma _0}{2}\right) ^{\beta _{d-1}}{\lceil \epsilon n \rceil \atopwithdelims ()d-1}$ such points of $Y_{\ell _0}\cap K$ (which are easily seen to be distinct due to the general position of *P*, and the generic choice of the lines of $\mathcal {{L}}(P,s)$). Therefore, the convex set *K* under consideration must have been pierced by a point of the 1-dimensional net $N_{\ell _0}\subseteq N_{\le \delta ,2}$, that was constructed over $Y_{\ell _0}$.

We keep the good simplex $\kappa \in {P'_K\atopwithdelims ()d-1}$ fixed, and distinguish between two possible scenarios.

**Case (i).** The good (d-2)-simplex $\kappa \in {P_K\atopwithdelims ()d-1}$ is not very good. Thus, there must exist a pair of simplices $\tau _1,\tau _2\in \Pi '_K(\ell _0)$ that are adjacent to $\kappa =\tau _1\cap \tau _2$, and a cutting cell $\varphi \in \mathcal {D}$ that is intersected by $H(\tau _1)$ but not $H(\tau _2)$.

**Fig. 34:**
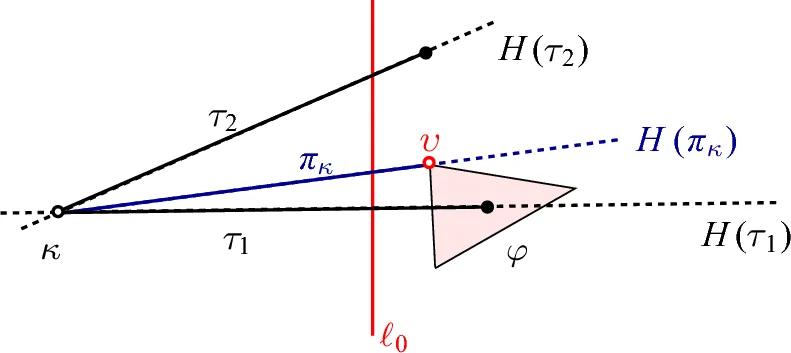
Proof of Lemma 6.5 – Case (i). The good (d-2)-simplex κ is not very good (view in $\mathbb {R}^3$, in the direction parallel to κ). There exist (d-1)-simplices $\tau _1,\tau _2\in \Pi '_K(\ell _0)$ so that $H(\tau _1)$ crosses a cell $\varphi \in \mathcal {D}$ that is missed by $H(\tau _2)$. There is a vertex *v* of φ, and a simplex $\pi _\kappa ={{\texttt{conv}}}(\kappa \cup \{v\})\in \Pi _X$, such that the hyperplane $H(\pi _\kappa )\in {\mathcal {H}}_X$ meets $\ell _0$ at a point of $Y_{\ell _0}$ between $\tau _1\cap \ell _0$ and $\tau _2\cap \ell _0$

Let us continuously rotate a hyperplane *H* around the (d-2)-flat ${{\texttt{aff}}}(\kappa )$, from $H(\tau _1)$ to $H(\tau _2)$, so that *H* never ceases intersecting the vertical line $\ell _0$. As is easy to check, there is exactly one direction that permits such a continuous rotation around κ. By continuity, there must be a point in time when the intersection $H\cap \varphi$ is about to vanish from *H*. By the general position, this can only happen when *H* coincides with a vertex *v* of φ and, therefore, supports the “synthetic” simplex $\pi _\kappa :={{\texttt{conv}}}(\kappa \cup \{v\})$ (so that $H=H\left( \pi _\kappa \right)$). Since *v* has been included in *X*, this simplex $\pi _\kappa$ belongs to $\Pi _X$. Hence, the set $Y_{\ell _0}$ must include the point $H\cap {\ell _0}$, which clearly lies in the vertical interval between the points $\tau _1\cap \ell _0$ and $\tau _2\cap \ell _0$, and well within $K\cap \ell _0$. See Fig. 34.

**Case (ii).** If the (d-2)-simplex $\kappa \in {P'_K\atopwithdelims ()d-1}$ is very good, then all of its $|\Gamma '_K(\kappa )|\ge (\vartheta /8d)(\sigma _0/2)^{\beta _{d-1}}\lceil \epsilon n\rceil$ neighbors $p\in \Gamma '_K(\kappa )$ must belong, in $\mathcal {D}$, to the zone of *every* hyperplane H(τ) that supports some κ-adjacent simplex $\tau \in \Pi '_K(\ell _0)$ (with $\kappa \subset \tau$).

Note that every κ-adjacent simplex $\tau \in \Pi '_K(\ell _0)$ yields a (tentative) assignment $H_K:=H(\tau )$ and $A_K:=\Gamma '_K(\kappa )$ which meets the first two criteria of Lemma 6.5, yet fails to meet the last one.

#### Definition

For any simplex $\tau \in \Pi '_K(\ell )$ that is adjacent to κ, let $\Gamma '_K(\kappa ,\tau )$ denote the subset of all such points in $\Gamma '_K(\kappa )$ that see H(τ).

It can be further assumed that every adjacent simplex $\tau \in \Pi '_K(\ell _0)$ of κ, satisfies

28 $$\begin{aligned} |\Gamma '_K(\kappa ,\tau )|<\frac{\vartheta }{16d}\left( \frac{\sigma _0}{2}\right) ^{\beta _{d-1}} \lceil \epsilon n \rceil , \end{aligned}$$

or, else, all the three conditions (i) – (iii) would have been met by the combination $H_K:=H(\tau )$ and $A_K:=\Gamma '_K(\kappa ,\tau )$.

Specializing to the $|\Gamma '_K(\kappa )|$ κ-adjacent simplices $\tau \in \Pi '_K(\ell _0)$, let us fix such a simplex $\tau _\kappa =\tau$ whose supporting hyperplane H(τ) attains the *lowest* possible $\ell _0$-intercept $H(\tau )\cap \ell _0$, and denote $H_\kappa :=H(\tau _\kappa )$. Clearly, the point $H_\kappa \cap \ell _0$ belongs to the interval $K\cap \ell _0$, for $\ell _0$ crosses the supporting simplex $\tau =\tau _\kappa$ of $H_\kappa$.

Combining the estimates (27) and (28) implies that the set $\Gamma '_K(\kappa )\setminus \Gamma '\left( \kappa ,\tau _\kappa \right)$ is comprised of at least

$$ |\Gamma '_K(\kappa )|-|\Gamma '_K(\kappa ,\tau _\kappa )|\ge \frac{\vartheta }{16d}\left( \frac{\sigma _0}{2}\right) ^{\beta _{d-1}} \lceil \epsilon n \rceil $$

such neighbouring points *p* of κ that *do not* see $H_\kappa$. Furthermore, it can be assumed, with no loss of generality, that at least half of these points in $\Gamma '_K(\kappa )\setminus \Gamma '\left( \kappa ,\tau _\kappa \right)$ lie *above* the hyperplane $H_\kappa$. Let $\Gamma _K^+(\kappa )$ denote this latter subset within $\Gamma '_K(\kappa )\setminus \Gamma '\left( \kappa ,\tau _\kappa \right)$, whose cardinality satisfies

$$ |\Gamma _K^+(\kappa )|>\frac{\vartheta }{32d}\left( \frac{\sigma _0}{2}\right) ^{\beta _{d-1}} \lceil \epsilon n \rceil . $$

**Fig. 35:**
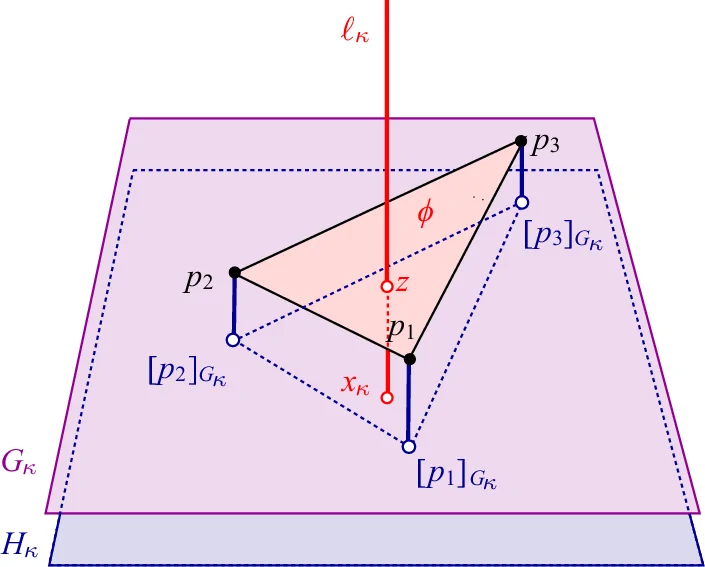
Proof of Lemma 6.5 in $\mathbb {R}^3$. The canonical line $\ell _\kappa \in \mathcal {{L}}(P,s)$ crosses at least one simplex ϕ, that is determined by $P_K^+(\kappa )$, at some point *z*. The vertices $p_i$ of ϕ are “blocked” from seeing $H_\kappa$ by the hyperplane $G_\kappa \in \mathcal {R}$. The depicted $\ell _\kappa$-intercept $x_\kappa \in X_\kappa$ of $G_\kappa$ too lies above $H_\kappa$

Notice that, for each $p\in \Gamma _K^+(\kappa )$, the vertical segment *e*(*p*, *H*), between *p* and $[p]_{H_\kappa }$, must be crossed by one or more hyperplanes $G\in \mathcal {R}$, so that, by the pigeonhole principle, one can fix such a hyperplane $G=G_\kappa \in \mathcal {R}$ that crosses at least

$$ |\Gamma ^+_K(\kappa )|/r\ge \frac{\vartheta }{32rd}\left( \frac{\sigma _0}{2}\right) ^{\beta _{d-1}} \lceil \epsilon n \rceil $$

segments in $$\{e(p,H_\kappa )\mid p\in \Gamma ^+_K(\kappa )\}$$.

Let $P^+_K(\kappa )$ denote the resulting subset $\{p\in \Gamma ^+_K(\kappa )\mid e(p,H_\kappa )\cap G_\kappa \ne \emptyset \}$. Note that the points of $P^+_K(\kappa )$ lie above both hyperplanes $G_\kappa$ and $H_\kappa$, and that

$$ |P_K^+(\kappa )|>\frac{\vartheta }{32rd}\left( \frac{\sigma _0}{2}\right) ^{\beta _{d-1}} \lceil \epsilon n \rceil \ge \varepsilon \cdot n/\vartheta \ge d, $$

where the second and the third inequalities follow from, respectively, the choice (7) of ε>0, and the lower bound (8) on *n* in Sect. 4. Since the convex set *K* is ε-spread in the vertical partition $\mathcal {V}(P,s)$, and the line family $\mathcal {{L}}(P,s)$ is ϑ-canonical with respect to $\mathcal {V}(P,s)$, there must be such a line $\ell _\kappa \in \mathcal {{L}}(P,s)$ that crosses at least *one* simplex ϕ whose *d* vertices belong to $P_K^+(\kappa )$. See Fig. 35.

Since $G_\kappa \in \mathcal {R}$, its $\ell _\kappa$-intercept $x_\kappa =\ell _\kappa \cap G_\kappa$ must belong to the previously defined subset $X_{\ell _\kappa } \subset X$, so that the simplex $\pi _\kappa :={{\texttt{conv}}}(\kappa \cup \{x_\kappa \})$ must belong to $\Pi _X$. Thus, the intersection point $y_\kappa =H(\pi _\kappa )\cap \ell _0$, between $\ell _0$ and the supporting plane $H(\pi _\kappa )$, must have been included in the set $Y_{\ell _0}$.

#### Proposition 6.7

Let $\kappa \in {P_K\atopwithdelims ()d-1}$ be a very good (d-2)-simplex. Then the previously described point $y_\kappa =H(\pi _\kappa )\cap \ell _0$ in $Y_{\ell _0}$ must lie in the interval $K\cap \ell _0$.

#### Proof of Proposition 6.7

In view of the choice of $\ell _\kappa$, one can fix a simplex $\phi ={{\texttt{conv}}}(p_1,\ldots ,p_d)$, that is crossed by $\ell _\kappa$ at some point *z*, and whose *d* vertices $p_1,\ldots ,p_d$ belong to the above set $P_K^+(\kappa )$. Let us emphasize that, as $P^+_K(\kappa )\subseteq \Gamma ^+_K(\kappa )$, each vertex $p_i$ of ϕ lies above the hyperplane $H_\kappa =H(\tau _\kappa )$. Furthermore, as each of the downward segments $e(p_i,G_\kappa )$ of the vertices of ϕ is crossed by the hyperplane $G_\kappa \in \mathcal {R}$, it follows, by the convexity of ϕ, that it lies entirely above $G_\kappa$, and the $\ell _\kappa$-intercepts of $H_\kappa$, $G_\kappa$ and ϕ appear along $\ell _\kappa$ in this increasing order of their *d*-th coordinates.

**Fig. 36:**
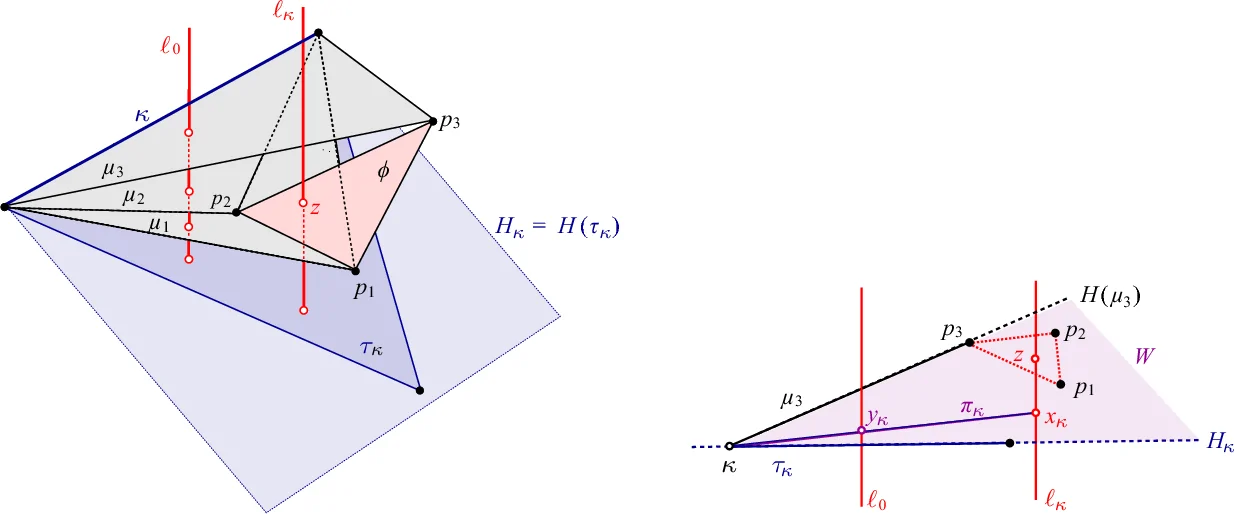
Proof of Proposition 6.7 in dimension d=3. Left: Depicted are the 4 simplices $\tau _\kappa$, $\mu _1$, $\mu _2$ and $\mu _3$, whose intercepts appear in this order along $\ell _0$. Right: Depicted in the κ-parallel direction is the dihedral wedge *W* which intersects $\ell _\kappa$ and contains $\phi ={{\texttt{conv}}}(p_1,p_2,p_3)$. Since $x_\kappa =\ell _\kappa \cap G_\kappa$ belongs to $X_{\ell _\kappa }$, and lies in *W*, the hyperplane through $\pi _\kappa =\kappa *\{x_\kappa \}$ lies within *W*, and meets $\ell _0$ at some point $y_\kappa$ of $Y_{\ell _0}\cap K$

Recall that each vertex $p_i$ of ϕ corresponds to a κ-adjacent simplex $\mu _i={{\texttt{conv}}}(\kappa \cup \{p_i\})$ of $\Pi '_K(\ell _0)\subseteq \Pi '_K$ (which, in particular, meets $\ell _0$). Assume with no loss of generality that the $\ell _0$-intercept $\mu _d\cap \ell _0$ of $\mu _d$ lies above the rest of such $\ell _0$-intercepts $\mu _i\cap \ell _0$, for $1\le i\le d-1$. See Fig. 36 (left). Consider the half-open dihedral wedge *W* between $H_\kappa$ and $H(\mu _d)$, which is comprised of all such points that lie above $H_\kappa =H(\tau _\kappa )$ and below (or at) $H(\mu _d)$. Then *W* contains the entire simplex $\phi ={{\texttt{conv}}}(p_1,\ldots ,p_d)$ and, moreover, its cross-section $W\cap \ell _0$ is contained in *K*, as it is delimited by the $\ell _0$-intercepts of the simplices $\tau _\kappa ,\mu _d\in \Pi '_K(\ell _0)$. See Fig. 36 (right).

Therefore, to “place” the intersection point $H(\pi _\kappa )\cap \ell _0$ within the interval $\ell _0\cap K$, it suffices to check that the point $x_\kappa =G_\kappa \cap \ell _\kappa$ too lies in the wedge *W* (so that the hyperplane $H(\pi _\kappa )$, through κ and $x_\kappa$, would remain in the same wedge *W* between $H(\tau _\kappa )$ and $H(\mu _d)$ and, thereby, cross $K\cap \ell _0$, at the aforementioned point $y_\kappa$). However, this easily follows from the fact that, by the convexity of *W* and ϕ, the point $z=\ell _\kappa \cap \phi$ lies in *W*; thus, $x_\kappa =e(z,H_\kappa )\cap G_{\kappa }$ has to lie below *z* (and, therefore, below $H(\mu _d)$), and above $H_\kappa$. □

To recap, Proposition 6.6 yields at least $(\vartheta /4)\left( \frac{\sigma _0}{2}\right) ^{\beta _{d-1}}{\epsilon n \atopwithdelims ()d-1}$ good (d-2)-simplices $\kappa \in {P'_K\atopwithdelims ()d-1}$. Each of these simplices κ (whether it is very good, or not) contributes a (d-1)-simplex $\pi _\kappa \in \Pi _X$ whose supporting hyperplane $H(\pi _\kappa )$ meets $\ell _0$ at a point of $Y_{\ell _0}\cap K$. Furthermore, the generic choice of $\mathcal {{L}}(P,s)$ easily implies that no pair of these simplices $\pi _\kappa ,\pi _{\kappa '}\in \Pi _X$ meet $\ell _0$ at the same point. Hence, the interval $K\cap \ell _0$ must contain at least $(\vartheta /4)\left( \frac{\sigma _0}{2}\right) ^{\beta _{d-1}}{\epsilon n \atopwithdelims ()d-1}$ points of $Y_{\ell _0}$ and, therefore, at least one point of the 1-dimensional net $N_{\ell _0}\subset N_{\le \delta ,2}$ (that was constructed over $Y_{\ell _0}$), which concludes the proof of Lemma 6.5. □

### Step 3

To finish the proof of Theorem 4.2, we describe the third net $N_{\le \delta ,3}$, which pierces all the remaining sets in ${{\mathcal {K}}}_{\le \delta }$, and then bound the cardinality of the entire net $N_{\le \delta }=\bigcup _{i=1}^3N_{\le \delta ,i}$.

**Setup.** Recall that every cell φ in our cutting $\mathcal {D}=\mathcal {D}(\mathcal {R},P)$ encompasses at most $\lfloor n/k\rfloor$ points of *P*, and is colored as either red or blue. Namely, the blue cells are the simplicial cells that have been inherited en toto from the bottom-vertex triangulation $\mathcal {D}(\mathcal {R})$ of the sampled arrangement $\mathcal {A}(\mathcal {R})$, and their number can be as large as $O\left( r^d\right)$. In contrast, the number of the red cells is at most 2*k*.

In view of Lemmas 6.3 and 6.5, every remaining set $K\in {{\mathcal {K}}}_{\le \delta }$ is now endowed with the principal hyperplane $H_K\in {\mathcal {H}}(\Pi '_K)$, which crosses at most $4c_{ cut}\cdot h\frac{k}{r}\log r=O\left( h\frac{k}{r}\log r\right)$ red cells of $\mathcal {D}$, and whose zone in $\mathcal {D}$ encompasses a subset $A_K\subseteq P_K$ of at least $c'_2 \sigma _0^{\beta _{d-1}} \lceil \epsilon n \rceil =\Theta (|P_K|)$ points which see $H_K$ within the underlying random arrangement $\mathcal {A}(\mathcal {R})$. (Here $c_{ cut}>0$ denotes the constant in Observation 6.1.)

#### Definition

For any open cell Δ in $$\mathcal {A}(\mathcal {R})$$, and any open cell φ in the cutting $$\mathcal {D}$$, let us denote $$P_\Delta :=P\cap \Delta $$, and $$P_\varphi :=P\cap \varphi $$. Note that both subsets $$P_\Delta $$ and $$P_\varphi $$ are contained in $$P'=P\setminus \left( \bigcup \mathcal {R}\right) $$, and every point of $P^{\prime }$ belongs to exactly one subset of each kind.

We will say that a point of $P^{\prime }$ is *blue* (resp., *red*) if it lies in a blue (resp., red) cell of $$\mathcal {D}(\mathcal {R},P)$$, and denote the resulting two subsets of $P^{\prime }$ by, respectively, *B* and *R*, so that $P^{\prime }=R⊎B$. Note that a given open cell Δ of $$\mathcal {A}(\mathcal {R})$$ may give rise to both red and blue cells within $$\mathcal {D}$$, so that its subset P(Δ) may include points of both color classes *R* and *B*.

**Overview.** Here is a high-level sketch of our divide-and-conquer treatment of the remaining sets $K\in {{\mathcal {K}}}_{\le \delta }$. Since at least half of the points in every subset $A_K$, that is induced by some remaining δ-hollow set $K\in {{\mathcal {K}}}_{\le \delta }$, must be of the same color, our task comes down to constructing a separate $\Theta (\epsilon )$-net $N_R$ (resp., $N_B$) over the point set *R* (resp., *B*).

1. *The net* $N_R$. Since the red points in every subset $A_K$ are distributed over $O\left( h\frac{k}{r}\log r\right)$ red cells $\varphi \in \mathcal {D}$, which are crossed by $H_K$, and each of these cells satisfies $|P_\varphi |\le \lfloor n/k\rfloor$, it is enough to construct for each of these cells $\varphi \in \mathcal {D}$ a $\tilde{\epsilon }$-net $N_\varphi$ with some $\tilde{\epsilon }>0$ that satisfies
  $$ \tilde{\epsilon }=\Theta \left( \frac{\epsilon n}{h\cdot \frac{k}{r}\log r\cdot (n/k)}\right) =\Theta \left( \frac{\epsilon \cdot r}{h\log r}\right) \gg \epsilon \cdot r^{1-\eta }, $$
  for the total cardinality $|N_R|=O\left( k \cdot f\left( \epsilon \cdot r^{1-\eta }\right) \right) =O\left( r^{\alpha _d}\cdot f\left( \epsilon \cdot r^{1-\eta }\right) \right)$. (Here the last estimate stems from our choice $k=r^{(d+\sqrt{d^2-2d})/2}$, which is bounded by $r^{\alpha _d}$ for all d≥3.)
2. *The net* $N_B$. If the majority of the points in the subset $A_K$ are blue, these points can be spread, within $\mathcal {D}$, over a far larger number of the blue cells. To still end up with an efficient recurrence, our decomposition of the blue set *B* will be based on the cells of the random arrangement $\mathcal {A}(\mathcal {R})$. A simple random sampling argument (to be shown in Lemma 6.10) will yield a (d-1)-dimensional polytope $T_K\subset H_K$ with the property that a fixed fraction of the blue points of $A_K$ lie in such cells Δ of $\mathcal {A}(\mathcal {R})$ that meet the relative boundary of $T_K$. According to Lemma 2.8 (applied within $H_K\cong \mathbb {R}^{d-1}$), the overall number of the latter cells Δ in $\mathcal {A}(\mathcal {R})$ is only $O\left( r^{d-2}\right)$. Though some of these cells $\Delta \in \mathcal {A}(\mathcal {R})$ may contain far more than $n/r^d$ points of *B*, Theorem 2.9 implies that such excessively “heavy” cells Δ encompass relatively few points of *B*, which can be “dispatched” by the means of a separate recursive $\Omega (\epsilon \log r)$-net. With the aforementioned exception, we will construct for each cell Δ in $\mathcal {A}(\mathcal {R})$ a separate $\epsilon _\Delta$-net, this time over the respective “blue” subset $B\cap \Delta$. Choosing a suitable threshold $\epsilon _\Delta$ for each cell $\Delta \in \mathcal {A}(\mathcal {R})$ will again result in a favourable upper bound on $|N_{B}|$, which involves only the terms of the “right” type $O\left( w^{\alpha _d+\eta }\cdot f\left( \epsilon \cdot w^{1-\eta }\right) \right)$ (where *w* is a fixed and positive power of *r*).

Let us now cast the missing details in the outlined construction of the nets $$N_R$$ and $$N_B$$, over the respective color classes *R* and *B* within $P^{\prime }$. To this end, let us fix a suitably small constant $$c'_3>0$$, which will be chosen in the sequel so as to suit the analysis of $$N_{>\delta ,3}$$. (This choice will depend on d≥3, η>0, $$\sigma _0>0$$, and also the constants $$c_0$$ and $$c'_2$$ in, respectively, Lemma 2.1 and Lemma 6.5.)

For the rest of this construction, and its subsequent analysis, it can be assumed that $\epsilon <c'_3$ for, otherwise, any of the previous methods [1, 24, 45] yields a constant-size net *N* of cardinality $O\left( 1/{c'_3}^{d+1}\right)$ for the entire family ${{\mathcal {K}}}(P,\epsilon )$.

In what follows, we use $\mathcal {D}_R$ (resp., $\mathcal {D}_B$) to denote the sub-family of all the red (resp., blue) cells in our cutting $\mathcal {D}=\mathcal {D}(\mathcal {R},P)$.

**The “red” net** $N_R$. For every red cell $\varphi \in \mathcal {D}$ we construct the net $N_\varphi :=N(P_\varphi ,\tilde{\epsilon })$ for the family ${{\mathcal {K}}}\left( P_\varphi ,\tilde{\epsilon }\right)$, with

29 $$\begin{aligned} \tilde{\epsilon }:=\frac{c'_2 \sigma _0^{\beta _{d-1}} \lceil \epsilon n\rceil }{8\cdot (n/k)\cdot c_{ cut}\cdot \frac{hk}{r}\log r}=\Theta \left( \frac{r}{h\log r}\cdot \epsilon \right) . \end{aligned}$$

We then set

$$ N_R:=\bigcup _{\varphi \in \mathcal {D}_R}N_\varphi . $$

**The “blue” net** $N_B$. Let

30 $$\begin{aligned} M:=c'_3\cdot \frac{k^2}{r^d(\log r)^{2\zeta _d+2}}, \end{aligned}$$

where $\zeta _d>0$ is the constant in Theorem 2.9.

For every cell $\Delta \in \mathcal {A}(\mathcal {R})$, let us denote $B_\Delta :=B\cap \Delta$. We then fix a threshold b:=n/M and classify the cells $\Delta \in \mathcal {A}(\mathcal {R})$ according to the number $|B_\Delta |$ of the blue points in each cell. The first sub-family $\mathcal {A}'$ is comprised of all such cells $\Delta \in \mathcal {A}(\mathcal {R})$ that satisfy either $|B_\Delta |<c'_3n/r^{d+2}$ or $|B_\Delta |\ge b$. The second sub-family $\tilde{\mathcal {A}}:=\mathcal {A}(\mathcal {R})\setminus \mathcal {A}'$ encompasses all the remaining cells Δ of $\mathcal {A}(\mathcal {R})$, which satisfy

$$ c'_3n/r^{d+2} \le |B_\Delta |<b. $$

The net $N_B$ combines the following ingredients.

1. *The nets* $N_\Delta$. For every cell $\Delta \in \tilde{\mathcal {A}}$, we include in $N_B$ the net $N_\Delta :=N\left( B_\Delta ,\epsilon _\Delta \right)$ for the family ${{\mathcal {K}}}\left( B_\Delta , \epsilon _\Delta \right)$, with
  31 $$\begin{aligned} \epsilon _\Delta :=c'_3\cdot \frac{\sigma _0^{\beta _{d-1}} \epsilon }{r^{d-2}}\frac{n}{|B_\Delta |}, \end{aligned}$$
2. *The net* $N_{B'}$. Lastly, we let $B':=\bigcup _{\Delta \in \mathcal {A}'}B_\Delta$, and add to $N_B$ the net $N_{B'}:=N\left( B',\epsilon \log r\right)$ for the family ${{\mathcal {K}}}\left( B',\epsilon \log r\right)$.

**The analysis.** The properties of our third (and last) net $N_{\le \delta ,3}:=N_R\cup N_B$ for the family ${{\mathcal {K}}}_{\le \delta }$ are summarized in the following lemma, whose somewhat technical proof is postponed to Sect. 6.4.

#### Lemma 6.8

1. With a suitably small choice of the constant $c'_3>0$, every remaining convex set in ${{\mathcal {K}}}_{\le \delta }$, that was missed by the combination $N_{\le \delta ,1}\cup N_{\le \delta ,2}$, is pierced by the just described net $N_{\le \delta ,3}=N_R\cup N_B$.
2. The cardinality of $N_{\le \delta ,3}$ satisfies
  $$\begin{aligned}&|N_{\le \delta ,3}|=O\left( f\left( \epsilon \log r\right) +k\cdot f\left( \epsilon \cdot r^{1-\eta }\right) \right. +\\&\quad \left. +\sum _{i=1}^{l} \frac{2^ik^2}{r^d}\cdot f\left( \epsilon \cdot 2^i\cdot \frac{k^2}{r^{2d-2+\eta /(10d)}}\right) \right) \end{aligned}$$
  for some l=O(logr).

### Proof of Lemma 6.8

**Part 1.** Let us first check that, provided a suitably small choice of the constant $c'_3>0$ in (30), every convex set $K\in {{\mathcal {K}}}_{\le \delta }$ (that survived so far) is pierced by the net $N_{\le \delta ,3}=N_R\cup N_B$. To this end, we fix such a set *K*, and distinguish between several scenarios according to the distribution of the (at least) $c'_2 \sigma _0^{\beta _{d-1}}\lceil \epsilon n\rceil$ points of the subset $A_K\subseteq P_K$ described in Lemma 6.5.

**Case (a).** Assume first that at least $|A_K|/2$ of all points in $A_K$ are red and, therefore, belong to the set $R=\bigcup _{\varphi \in \mathcal {D}_R}P_\varphi$. Since the principal hyperplane $H_K$ belongs to ${\mathcal {H}}(\Pi ')$ and, therefore, crosses a total of at most $4c_{ cut}\cdot h\frac{k}{r}\log r$ red cells $\varphi \in \mathcal {D}_R$, there must be such a cell $\varphi \in \mathcal {D}_R$ that encompasses at least

$$ \frac{c'_2 \sigma _0^{\beta _{d-1}}\epsilon n}{8c_{ cut}\cdot h\frac{k}{r}\log r}\ge \tilde{\epsilon }\cdot \frac{n}{k}\ge \tilde{\epsilon }\cdot |P_\varphi |. $$

points of $A_K$ (where the second inequality uses the definition (29) of $\tilde{\epsilon }$). Hence, such a set *K* is pierced by the local net $N_\varphi \subseteq N_{R}$, which was constructed for the instance ${{\mathcal {K}}}\left( P_\varphi ,\tilde{\epsilon }\right)$.

**Case (b).** Let us assume, then, that at least $|A_K|/2\ge c'_2 \sigma _0^{\beta _{d-1}}\lceil \epsilon n\rceil /2$ among the points of $A_K$ belong to *B* and, thereby, lie in the blue cells $\varphi \in \mathcal {D}_B$. In view of the (potentially) very non-uniform distribution of the blue points between the cells of $\mathcal {A}(\mathcal {R})$, two sub-scenarios are to be considered.

**Case (b1).** If $|A_K\cap B'| \ge |A_K|/4$, then a suitably small choice of $c'_3>0$ yields

$$ |A_K\cap B'|\ge c'_2 \sigma _0^{\beta _{d-1}}\lceil \epsilon n\rceil /4\ge \epsilon \log r \cdot |B'|, $$

where the last inequality follows from Proposition 6.9 below. As a result, such a set *K* is $$(\epsilon \log r)$$-heavy with respect to $B^{\prime }$ and, therefore, must have been pierced by the net $$N_{B'}\subseteq N_B$$.

#### Proposition 6.9

With a suitably small choice of the constant $c'_3>0$ in the definition of $N_{\le \delta ,3}$, we have that $|B'|\le c'_2\sigma _0^{\beta _{d-1}} n/\left( 6\log r\right)$.

#### Proof

Since $|\mathcal {A}'|\le |\mathcal {A}(\mathcal {R})|=O\left( r^d\right)$, the cells of $\Delta \in \mathcal {A}'$ that satisfy $|B_\Delta |< c'_3 n/r^{d+2}$ account for a total of

32 $$\begin{aligned} O\left( |\mathcal {A}(\mathcal {R})|\cdot n/r^{d+2}\right) =O\left( n/r^2\right) \end{aligned}$$

points of $B^{\prime }$.

The remaining points of $B^{\prime }$ are covered by at most $$\lfloor n/b\rfloor \le M$$ cells of $$\mathcal {A}'$$ which satisfy $$|B_\Delta |\ge b$$. Using the estimate of Theorem 2.9 (and the definition (30) of *M*), we obtain the bound

33 $$\begin{aligned} O\left( M^{1/2}r^{d/2}(\log r)^{\zeta _d}\right) =O\left( k/\log r\right) \end{aligned}$$

on the overall complexity of the latter cells Δ in $$\mathcal {A}'$$. Hence, their bottom vertex triangulation yields a total of $$O\left( k/\log r\right) $$ simplices of $$\mathcal {D}_B$$, each of them containing at most *n*/*k* points of $B^{\prime }$, for a total amount of $$O\left( \left( k/\log r\right) \cdot (n/k)\right) =O\left( n/\log r\right) $$ points.

Provided a small enough, albeit, fixed choice of $c'_3>0$ (which depends on $\sigma _0$ and $c'_2$), the implicit constants of proportionality on the right hand sides of both estimates (32) and (33) are small enough to ensure that the cells of $\mathcal {A}'$ encompass a total of at most $c'_2 \sigma ^{\beta _{d-1}}n/(6\log r)$ points of *B*. □

**Case (b2).** We have that

34 $$\begin{aligned} \left| A_K\cap \tilde{B}\right| \ge |A_K|/4\ge c'_2 \sigma _0^{\beta _{d-1}}\lceil \epsilon n\rceil /4, \end{aligned}$$

where $\tilde{B}$ denotes the set $B\setminus B'=\bigcup _{\Delta \in \tilde{\mathcal {A}}} B_\Delta$. Then the claim is an straightforward consequence of the following observation.

#### Lemma 6.10

Let $K\in {{\mathcal {K}}}_{\le \delta }$ be a convex set that is missed by $N_{\le \delta ,1}\cup N_{\le \delta ,2}$, and that falls into case (b2). Then, provided a sufficiently small choice of the constant $c'_3>0$, there is a (d-1)-dimensional convex polytope $T_K$ within $H_K$, and a subset $B_{K}\subseteq A_K\cap \tilde{B}$ of at least $|A_K|/12$ such blue points whose ambient cells in $\mathcal {A}(\mathcal {R})$ meet the relative boundary of $T_K$ within $H_K$.

To complete the proof of Lemma 6.8 using Lemma 6.10, let $\tilde{\mathcal {A}}_K$ denote the subset of all such cells $\Delta \in \tilde{\mathcal {A}}$ that meet the relative boundary of the (d-1)-dimensional polytope $T_K\subset H_K$ described in Lemma 6.10. Notice that every cell $\Delta \in \tilde{\mathcal {A}}_K$ corresponds to a unique (d-1)-dimensional cell $\Delta \cap H_K$, which too meets the relative boundary of $T_K$, and is part of the arrangement that is determined by the family $\{H\cap H_K\mid H\in \mathcal {R}\}$ of (d-2)-flats within $H_K\equiv \mathbb {R}^{d-1}$. Applying Lemma 2.8 to this (d-1)-dimensional arrangement implies the estimate $|\tilde{\mathcal {A}}_K|\le cr^{d-2}$, with a suitable constant c>0 which depends on the dimension d≥3. As a result, the pigeonhole principle yields such a cell $\Delta \in \tilde{\mathcal {A}}_K$ that satisfies

$$ |B_K\cap B_\Delta |\ge \frac{|B_K|}{cr^{d-2}}\ge \frac{|A_K|}{12cr^{d-2}}\ge \frac{c'_2}{12cr^{d-2}}\cdot \sigma _0^{\beta _{d-1}}\lceil \epsilon n\rceil . $$

Hence, provided that $c'_3\le c'_2/(12c)$, such a convex set *K* must have been pierced by the “local” net $N_\Delta \subseteq N_B$, which was constructed for the family ${{\mathcal {K}}}\left( B_\Delta ,\epsilon _\Delta \right)$.

#### Proof of Lemma 6.10

Let us fix a convex set $K\in {{\mathcal {K}}}_{\le \delta }$ as in the hypothesis, and denote $\tilde{B}_K:=A_K\cap \tilde{B}$. Consider the partition $\left\{ A_K\cap B_\Delta \mid \Delta \in \tilde{\mathcal {A}}\right\}$ of $\tilde{B}_K$, by the cells of $\tilde{\mathcal {A}}$, into $|\tilde{\mathcal {A}}|\le (d+1){r\atopwithdelims ()d}\le r^d$ subsets (see, e.g., [43, Proposition 6.1.1]). According to Lemma A.2, a random $\tilde{r}$-sample $S\subseteq \tilde{B}_K$, with $\tilde{r}=\min \left\{ \Big \lceil 10^3 r^d\Big \rceil ,|\tilde{B}_K|\right\}$, meets the following condition with probability at least 1/2:

(C1) *The cells of* $\tilde{\mathcal {A}}_S=\{\Delta \in \tilde{\mathcal {A}}\mid S\cap \Delta \ne \emptyset \}$ *encompass a total of at least* $|\tilde{B}_K|/3$ *points of* $\tilde{B}_K$.

Furthermore, the fact that the principal subset $P_K$ is δ-hollow implies, in particular, that there exist at most $\delta {\lceil \epsilon n\rceil \atopwithdelims ()d+1}$ punctured (d+1)-subsets in ${\tilde{B}_K\atopwithdelims ()d+1}$. Therefore, and because of (34), the point set $\tilde{B}_K$ must be $\tilde{\delta }$-hollow, with $\tilde{\delta }\ge 0$ that satisfies

$$ \tilde{\delta }\le \delta {\lceil \epsilon n\rceil \atopwithdelims ()d+1}/{|\tilde{B}_K|\atopwithdelims ()d+1} \le \delta {\lceil \epsilon n\rceil \atopwithdelims ()d+1}/{\lceil c'_2 \sigma _0^{\beta _{d-1}} \epsilon n\rceil /4\atopwithdelims ()d+1} =O(\delta )=O\left( 1/r^{d(d+1)+1}\right) . $$

**Fig. 37:**
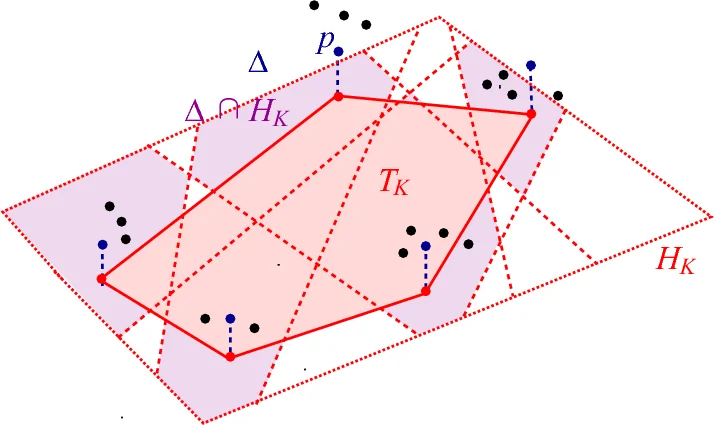
Proof of Lemma 6.10. We sample a 0-hollow subset *S* of $\tilde{r}=\min \left\{ \lceil 10^3r^d\rceil ,|\tilde{B}_K|\right\}$ blue points from $\tilde{B}_K$. The projected red points of $[S]_{H_K}$ determine the (d-1)-dimensional convex polytope $T_K:={{\texttt{conv}}}\left( [S]_{H_K}\right)$ within the principal hyperplane $H_K$. The cross-sections $\Delta \cap H_K$ of the ambient cells $\Delta \in \mathcal {A}(\mathcal {R})$ of the points p∈S are shaded as well

Since we have that $r=\Big \lceil (1/\epsilon )^{\tilde{\eta }^3}\Big \rceil$, $\tilde{\delta }=o\left( 1/r^{d(d+1)}\right)$, and $\epsilon <c'_3$, any sufficiently small, albeit fixed choice of $c'_3>0$ (which does not depend on the on the quantities ϵ and *n*) yields the inequality

$$ \tilde{\delta }\le \frac{c_0}{\Big \lceil 10^3 r^d \Big \rceil ^{(d+1)}}\le \frac{c_0}{\tilde{r}^{d+1}}. $$

According to Lemma 2.1, the sample *S* is 0-hollow with probability at least 0.99 (as depicted in Fig. 37). Hence, with probability at least 0.4, it both (i) satisfies the previous condition (C1), and (ii) is 0-hollow.

Let us assume, then, that both criteria hold for the sample *S* at hand. Consider its $H_K$-projection $[S]_{H_K}:=\{[p]_{H_K}\mid p\in S\}$. We claim that the (d-1)-dimensional polytope $T_K:={{\texttt{conv}}}\left( [S]_{H_K}\right) \subset H_K$ meets the criteria of the proposition with $B_K:=\tilde{B}_K\cap \left( \bigcup \tilde{\mathcal {A}}_S\right)$. In view of property (C1), it suffices to check that every cell of $\tilde{\mathcal {A}}_S$ meets the relative boundary of $T_K$ within $H_K$. Indeed, fix such a cell $\Delta \in \tilde{\mathcal {A}}_S$, with a point $p\in S\cap \Delta$. Then, by the 0-hollowness of our sample *S*, the point *p* projects to a boundary vertex $[p]_{H_K}$ of $T_K$. Furthermore, since *p* sees $H_K$ (as $S\subseteq A_K$), the vertex $[p]_{H_K}$ too lies in the cell Δ, which then intersects the relative boundary of $T_K$. □

**Part 2.** To complete the proof of Lemma 6.8, let us now bound the cardinality of $N_{\le \delta ,3}=N_R\cup N_B$. To estimate $|N_R|$, recall that $|\mathcal {D}_{R}|\le 2k$. Together with that fact that $h\lll _\eta r\lll _\eta 1/\epsilon$, this yields

$$ |N_R|=\sum _{\varphi \in \mathcal {D}_{R}} \left| N_\varphi \right| =O\left( k\cdot f\left( \frac{\sigma _0^{\beta _{d-1}}r}{h\log r}\cdot \epsilon \right) \right) =O\left( k\cdot f\left( \epsilon \cdot r^{1-\eta }\right) \right) . $$

To bound the remaining quantity $|N_B|=|N_{B'}|+\sum _{\Delta \in \tilde{\mathcal {A}}}|N_\Delta |$, note first that $|N_{B'}|\le f\left( \epsilon \log r\right)$. To estimate the overall cardinality of the remaining “blue” nets $N_\Delta$, let us arrange their respective cells $\Delta \in \tilde{\mathcal {A}}$ into sub-families with roughly the same density of the blue points in each cell. To this end, we fix $l:=\log \lceil r^{d+2}/M\rceil =O(\log r)$, and set

$$ \tilde{\mathcal {A}}_i:=\left\{ \Delta \in \tilde{\mathcal {A}}\ \Bigg \vert \frac{b}{2^i} \le |B_\Delta |<\frac{b}{2^{i-1}}\right\} $$

for all $1\le i\le l$. Notice that $\tilde{\mathcal {A}}=\biguplus _{i=1}^l\tilde{\mathcal {A}}_i$ and, according to (31), every cell $\Delta \in \mathcal {A}_i$ satisfies

$$\begin{aligned} \epsilon _\Delta =c'_3\cdot \frac{\sigma _0^{\beta _{d-1}} \epsilon }{r^{d-2}}\frac{n}{|B_\Delta |}=\Theta \left( \frac{\epsilon }{r^{d-2}}\cdot 2^iM\right) =\Theta \left( \frac{2^i k^2\epsilon }{r^{2d-2}\cdot (\log r)^{2\zeta _d+2}}\right) . \end{aligned}$$

Since we have that

$$ \left| \tilde{\mathcal {A}}_i\right| \le 2^i n/b=\Theta (2^i\cdot M)=\Theta \left( \frac{2^i k^2}{r^d(\log r)^{2\zeta _d+2}}\right) , and $$

and $\log r\lll _\eta r$, it follows that

$$ \sum _{\Delta \in \tilde{\mathcal {A}}_i}\left| N_\Delta \right| \le \left| \tilde{\mathcal {A}}_i\right| \cdot f\left( \frac{2^i k^2\epsilon }{r^{2d-2}\cdot (\log r)^{2\zeta _d+2}}\right) =O\left( \frac{2^ik^2}{r^{d}}f\left( \epsilon \cdot \frac{2^i k^2}{r^{2d-2+\eta /(10d)}}\right) \right) $$

for all $1\le i\le l$, which concludes the proof of the second part of Lemma 6.8. □

### Wrap-Up for the δ-Hollow Sets

According to the first part of Lemma 6.8, every set in ${{\mathcal {K}}}_{\le \delta }$ is pierced by the combined net

$$ N_{\le \delta }=N_{\le \delta ,1}\cup N_{\le \delta ,2}\cup N_{\le \delta ,3}. $$

Putting together the bounds in Lemmas 6.3, 6.4, and 6.8 yields

$$ |N_{\le \delta }|\le f(\epsilon ,\lambda /h,\sigma /2)+f\left( \epsilon \log r\right) + $$

$$ +O\left( k\cdot f\left( \epsilon \cdot r^{1-\eta }\right) +\sum _{i=1}^{l} \frac{2^ik^2}{r^d}\cdot f\left( \epsilon \cdot 2^i\cdot \frac{k^2}{r^{2d-2+\eta /(10d)}}\right) \right) +O\left( \frac{1}{\epsilon ^{d-1+\eta }}\right) , $$

where l=O(logr). Recalling our choice $k=r^{(d+\sqrt{d^2-2d})/2}=r^{d-1/2-o_d(1)}$, and fixing $w:=k^2/r^{2d-2+\eta /(10d)}$, yields

$$\begin{aligned}&|N_{\le \delta }|\le f(\epsilon ,\rho /h,\sigma /2)+f\left( \epsilon \cdot \log r \right) +O\left( r^{(d+\sqrt{d^2-2d})/2+\eta }\cdot f\left( \epsilon \cdot r^{1-\eta }\right) +\right. \\&\quad \left. +\sum _{i=1}^{l} 2^iw^{(d+\sqrt{d^2-2d})/2+\eta }\cdot f\left( \epsilon \cdot 2^i w\right) \right) +O\left( \frac{1}{\epsilon ^{d-1+\eta }}\right) . \end{aligned}$$

Since we have that $\alpha _d\ge (d+\sqrt{d^2-2d})/2$ for all d≥3, this completes the proof of Theorem 4.2. □

## Concluding Remarks

- Despite the upper bound $f_d(\epsilon )=O\left( (1/\epsilon )^{d-1/2-o_d(1)}\right)$ that Theorem 1.1 yields in all dimensions d≥4, the present analysis cannot be used to show that $f_2(\epsilon )=o\left( (1/\epsilon )^{3/2}\right)$. (Nevertheless, it can be easily adapted to show a weaker bound $f_2(\epsilon )=O\left( (1/\epsilon )^{a}\right)$, for some constant 3/2<a<2.)
- The author conjectures that the actual asymptotic behaviour of the functions $f_d(\epsilon )$ in any dimension d≥1 is close to 1/ϵ, as is indeed the case for their “strong” counterparts which exist with respect to simply shaped objects in $\mathbb {R}^d$ [36].
- The author anticipates that a major improvement of the upper bound on $f_d(\epsilon )$ (i.e., beyond $O^*\left( 1/\epsilon ^{d-1}\right)$) would require stronger selection-type results for piercing simplices with points, lines and other flats, in the general vein of Theorem 3.2.
- The recently improved analysis of the transversal numbers $C_d(p,q)$ that arise in the Hadwiger-Debrunner problem (see Sect. 1), due to Keller, Smorodinsky, and Tardos [40], implies that
  35 $$\begin{aligned} C_d(p,q)\le f_d\left( \Omega \left( p^{-1-\frac{d-1}{q-d}}\right) \right) . \end{aligned}$$
  Here, as before, $f_d(\epsilon )$ denotes the smallest possible number *f* with the property that any *n*-point set $P\subset \mathbb {R}^d$ admits a weak ϵ-net of cardinality *f* with respect to convex sets.
  Plugging the result of Theorem 1.1, and the previous planar bound [53], into (35) yields improved bounds in all dimensions d≥2, namely,
  $$ C_2(p,q)=O\left( p^{(3/2+\gamma )\left( 1+\frac{d-1}{q-d}\right) }\right) $$
  and
  $$ C_d(p,q)=O\left( p^{(\alpha _d+\gamma )\left( 1+\frac{d-1}{q-d}\right) }\right) , $$
  for all d≥3. Here γ>0 is an arbitrary positive constant, and *p* has to be larger than a certain constant threshold which depends on γ.
- Holmsen and Lee [37, 38] showed that weak ϵ-nets of cardinality $O\left( 1/\epsilon ^{c_r}\right)$ exist for convex sets in any abstract convexity space whose so called Radon number *r* is bounded; here $c_r$ is a (potentially enormous) constant that depends on *r*. (The *Radon number* of a convexity space is the smallest number *r* with the following property: any set *A* of *r* elements admits a partition A=B⊎C so that ${{\texttt{conv}}}(B)\cap {{\texttt{conv}}}(C)\ne \emptyset$). In that rather more general setting, it would comprise a significant progress to obtain a weak ϵ-net construction of cardinality $O\left( 1/\epsilon ^{cr}\right)$, where *c* is a constant that does not depend on the Radon number *r*.
- Our proof of Theorem 1.1 is fully constructive and combines the following explicit ingredients across the various instances ${{\mathcal {K}}}\left( P,\Pi ,\epsilon ,\sigma \right)$ which are encountered in the course of our recurrence in ϵ>0, the ground set *P*, and the subset $\Pi \subseteq {P\atopwithdelims ()d}$ of (d-1)-simplices:
  1. 1-dimensional strong $\Omega ^*(\epsilon ^{\alpha _d})$-nets that are constructed via Theorem 3.3 within the lines ℓ of the canonical families $\mathcal {{L}}(P,s)$. Each of these nets is defined over the ℓ-intercepts $\ell \cap \tau$ of the (d-1)-simplices $\tau \in \Pi$.
  2. 1-dimensional strong $$\Omega ^*\left( \epsilon ^\alpha \right) $$-nets that are obtained in Step 2 of the construction of $$N_{>\delta }$$, for the “secondary” canonical lines $$\ell \in \mathcal {{L}}(P_i,u)$$. Each of these nets $$N'_\ell $$ is defined with respect to the ℓ-intercepts of the $$O^*\left( t^d\right) $$ auxiliary (d-1)-simplices of V′d (where the set $V^{\prime }$ is comprised of all the vertices of the clipped cells $\varphi^{\prime }\left(p\right)$ which have been constructed around the points p∈P).
  3. 1-dimensional strong $$\Omega ^*\left( \epsilon ^{d-1}\right) $$-nets that are obtained in Step 2 of the construction of $$N_{\le \delta }$$, for the lines $$\ell \in \mathcal {{L}}(P,s)$$. Each of these nets $$N_\ell $$ over the ℓ-intercepts of the “mixed” (d-1)-simplices of $${P\atopwithdelims ()d-1}*X$$. (The set *X* is comprised of the $\ell^{\prime }$-intercepts of the sampled hyperplanes of $$\mathcal {R}$$, taken over all canonical lines $$\ell '\in \mathcal {{L}}(P,s)$$, and also of the vertices of the cutting $$\mathcal {D}$$.)
  4. Strong $\Theta \left( \frac{\delta \epsilon }{st^{1-1/d}}\right) =\Omega (\epsilon ^{\alpha _d})$-nets that are constructed in Sect. 5.2, via Lemma A.1, with respect to convex “(2*d*)-hedra”.
- As the primary focus of this study is on the quantity $f_d(\epsilon )$, we did not seek to optimize the construction cost of our net *N*. A straightforward implementation of the recursive construction runs in time $O^*\left( n^{d}\right)$, which is dominated by the non-recursive overhead spent on maintaining the sets $\Pi \subseteq {P\atopwithdelims ()d}$ and tracing the zones of the hyperplanes of ${\mathcal {H}}(\Pi )$ within the cutting $\mathcal {D}=\mathcal {D}(P,\mathcal {R})$ of Sect. 6. This also accounts for the cost of constructing the 1-dimensional nets of Theorem 3.3 with respect to the ℓ-intercepts $\ell \cap \tau$, for all simplices $\tau \in \Pi$ and canonical lines $\ell \in \mathcal {{L}}(P,s)$.
  Since $s\lll _\eta u\lll _\eta 1/\epsilon$, all the vertical partitions $\mathcal {V}(P,s)$ and $\mathcal {V}(P_i,u)$, along with the induced canonical line families $\mathcal {{L}}(P,s)$ and $\mathcal {V}(P_i,u)$, can be obtained in $O^*(n)$ time via Matoušek’s Theorem 2.6. Since $r\lll _\eta s$, the same holds true for the arrangement $\mathcal {A}(\mathcal {R})$ and its cutting $\mathcal {D}=\mathcal {D}(\mathcal {R},P)$ in Sect. 6, and the assignment of the points of *P* to their ambient cells in $\mathcal {A}(\mathcal {R})$ and $\mathcal {D}$. Furthermore, as Matoušek’s partition theorem holds with any partition parameter $1\le r\le n$, the secondary partition $\mathcal {P}(P,s,t)$ in Sect. 5 can be constructed in similar time $O^*(n)$. Armed with the partition $\mathcal {P}(P,s,t)$, the set $\tilde{\Pi }$ in Lemma 5.6 can be obtained in $O\left( |\Lambda (P,s,t)|\cdot (st)^{d-1}+n^d\right) =O\left( n^d\right)$ time.
- It would be interesting to obtain a more elementary weak epsilon-net construction in all dimensions d≥2, whose cardinality is close to $1/\epsilon ^{d-1/2}$ (or at least far smaller than $1/\epsilon ^d$), and that can be implemented in $O^*(n)$ time.

## Random Sampling and Strong ϵ-nets

Let *X* be a (finite) set of elements and $\mathcal {F}\subset 2^X$ be a set of hyperedges spanned by *X*. A *strong* ϵ*-net* for the hypergraph $(X,\mathcal {F})$ is a subset Y⊆X so that $F\cap Y\ne \emptyset$ holds for all the hyperedges $F\in \mathcal {F}$ that satisfy $|F|\ge \epsilon n$.

The Epsilon-Net Theorem of Haussler and Welzl [36] states that any hypergraph $(X,\mathcal {F})$, as above, that is drawn from a so called range space of a bounded VC-dimension D>0, admits a strong ϵ-net *Y* of cardinality $r=O\left( \frac{D}{\epsilon }\log \frac{D}{\epsilon }\right)$. Moreover, such a net *Y* can obtained, with probability at least 1/2, by choosing an *r*-sample of *X*. In particular, this implies the following property which will be used in the proof of Theorem 1.1.

### Lemma A.1

Let ϵ>0. Then any finite point set *P* in the Euclidean space $\mathbb {R}^d$ of fixed dimension d≥1, contains a subset *Q* of $O\left( \frac{1}{\epsilon }\log \frac{1}{\epsilon }\right)$ points piercing all the ϵ-heavy polytopes in $\mathbb {R}^d$ whose boundaries contain at most 2*d* vertices; the implicit constant of proportionality depends on the dimension *d*.

In addition to strong ϵ-nets, in Sect. 6.4 we will use the following more elementary property of *r*-samples.

### Lemma A.2

Let *A* be a set of *m* elements, and let $$A=A_1\uplus A_2\uplus \ldots \uplus A_r$$ be a partition of *A* into r≥1 parts. Let *S* be a random $r^{\prime }$-sample of *A*, so that $$r'=\min \{10^3 r,m\}$$. Then, with probability at least 1/2, the parts $$A_i$$ that intersect *S* encompass at least *m*/3 elements of *A*.

### Proof

If $$m\le 10^3r$$, then we have that S=A, and there is nothing to prove. Otherwise, note that at most *m*/10 elements of *A* can belong to the partition sets $$A_i$$ with $$|A_i|\le m/(10r)$$. Let $$A':=\bigcup \left\{ A_i\mid 1\le i\le r, |A_i|>m/(10r)\right\} $$. Then we have $|A^{\prime }|>m-r\cdot m/\left(10r\right)=9r/10$. Assume with no loss generality that $$r'<m(1-\frac{1}{10r})$$ (or, else, the claim again holds with probability 1). Fix any $x\in A^{\prime }$ with the ambient set $$A_{i(x)}$$, so that $$1\le i(x)\le r$$. Since $$|A_{i(x)}|> m/(10r)$$, the probability $${\boldsymbol{p}}_x$$ that $$A_{i(x)}$$ is missed by the sample *S* satisfies

$$ {\boldsymbol{p}}_x\le {\lfloor m-m/(10r)\rfloor \atopwithdelims ()r'}/{m\atopwithdelims ()r'}\le {\left( 1-\frac{1}{100r}\right) }^{10^3r}\le 1/100. $$

Hence, the expected number of elements in the union $\bigcup _{x\in R}A_{i(x)}$ of the parts that intersect *S*, is

$$ \sum _{x\in A'}{\boldsymbol{p}}_x\ge 0.99\cdot 99\,m/100. $$

Now the lemma follows by Markov’s inequality. □

## Elementary Properties of Point Sets and Hyperplanes

### Proof of Lemma 2.1

It can be assumed that r≥d+1, or else the 0-hollowness holds trivially, with S=A and ${S\atopwithdelims ()d+1}=\emptyset$. Let $B\in {A \atopwithdelims ()d+1}$ be a punctured (d+1)-size subset. Notice that *B* arises in our *r*-sample S⊆A with probability $\displaystyle {\boldsymbol{p}}:=\frac{{m-d-1\atopwithdelims ()r-d-1}}{{m\atopwithdelims ()r}}=\Theta \left( \frac{r^{d+1}}{m^{d+1}}\right)$. Hence, the expected number of such (d+1)-subsets $B\in {A\atopwithdelims ()d+1}$ is at most

36 $$\begin{aligned} \delta {m\atopwithdelims ()d+1} {\boldsymbol{p}}=O\left( \delta {m\atopwithdelims ()d+1}\left( \frac{r}{m}\right) ^{d+1}\right) . \end{aligned}$$

A suitably small choice of the constant $c_0>0$ guarantees that the left-hand side of (36) is always smaller than 1/100. In particular, this implies that, with probability at least 99/100, *all* the (d+1)-size subsets in Sd+1 are hollow. □

### Proof of Lemma 2.2

Notice that, since $\Delta _0$ intersects ${{\texttt{conv}}}\left( \bigcup _{i=1}^{d+1} \Delta _i\right)$, there must exist points $p_0\in \Delta _0,p_1\!\in \!\Delta _1,\!\ldots \!,p_{d+1}\in \Delta _{d+1}$ so that $p_0\!\in \!{{\texttt{conv}}}(p_1,\!\ldots \!,p_{d+1})$.^33^ The claim of the lemma follows immediately if there exists a hyperplane *H* that contains $p_0$ together with some *d* points among $p_1,\ldots ,p_{d+1}$. Assume, then, that there is no such hyperplane *H*; hence, $p_0$ lies in the interior of ${{\texttt{conv}}}(p_1,\ldots ,p_{d+1})$. However, since $\Delta _0$ is *not* surrounded by $\Delta _1,\ldots ,\Delta _{d+1}$, there must also exist some points $q_0\in \Delta _0,q_1\in \Delta _1,\ldots ,q_{d+1}\in \Delta _{d+1}$ so that $q_0$ does not lie in the interior of ${{\texttt{conv}}}(q_1,\ldots ,q_{d+1})$. For each t∈[0,1], and each $0\le i\le d+1$, we define the point $q_i(t):=(1-t)p_i+tq_i$, which clearly lies within the convex set $\Delta _i$, and use τ(t) to denote the convex hull ${{\texttt{conv}}}(q_1(t),\ldots ,q_{d+1}(t))$. Notice that $\tau (0)={{\texttt{conv}}}\{p_i\mid 1\le i\le d+1\}$ contains the point $p_0=q_0(0)$ in its interior, whereas the interior of $\tau (1)={{\texttt{conv}}}\{q_i\mid 1\le i\le d+1\}$ no longer contains $q_0=q_0(1)$. Thus, there must be $t^*\in [0,1]$ so that $q_0(t^*)$ lies on the boundary of $\tau (t^*)$. It can, therefore, be assumed, with no loss of generality, that $q_0(t^*)\in {{\texttt{conv}}}(q_1(t^*),\ldots ,q_{d}(t^*))$, so that any hyperplane through ${{\texttt{conv}}}(q_1(t^*),\ldots ,q_{d}(t^*))$ contains $q_0(t^*)$ and intersects $\Delta _1,\ldots ,\Delta _{d}$ at the respective points $q_1(t^*),\ldots ,q_{d}(t^*)$. □

### Proof of Lemma 2.3

Let *H* be any hyperplane transversal to Σ. We continuously translate *H* in a fixed direction until the first time *H* meets a vertex $v_1$ of some simplex $\Delta _{i_1}\in \Sigma$. We then continuously rotate *H* in a fixed direction around $v_1$, so the normal to *H* is moving along a fixed great circle of ${\mathbb {S}}^{d-1}$, until the first time *H* hits an additional vertex $v_2$ of a simplex $\Delta _{i_2}\in \Sigma$ (where $i_1$ is not necessarily different from $i_2$). This procedure is repeated d-1 times, each time adding a vertex $v_j$ of some simplex $\Delta _{i_j}\in \Sigma$, for $2\le j\le d$, to the hyperplane *H* which we rotate around the affine hull of the previously added vertices $v_1,\ldots ,v_{j-1}$. (This is possible because the vertex sets $V(\Delta _i)$ of the simplices $\Delta _i\in \Sigma$ encompass a total of at least *d* vertices, whilst *H* never contains more than *d* of these vertices.) Once *H* encounters a new vertex $v_{i_j}$, this vertex never leaves *H*, which reduces by 1 the available degrees of freedom that are available to the following rotation step. The process terminates when *H* contains *d* vertices, so its rotation is no longer possible.

Notice that, at every step, *H* meets every closed simplex $\Delta _{j}\in \Sigma$, for the contact between *H* and $\Delta _j$ cannot be lost before *H* “gains” at least one of the vertices of $\Delta _j$. □

## Solving the Recurrence for f(ϵ)

To solve the recurrence (13) in the end of Sect. 4.3, let us fix a small enough auxiliary threshold $$\epsilon '_0=\epsilon '_0(d,\gamma )>0$$ so as to guarantee that all the recursive terms $f(\epsilon^{\prime })$ in the right hand side of (13) involve arguments that satisfy $$\epsilon '>2\epsilon $$ whenever $$\epsilon \le \epsilon '_0$$.^34^ Since the inequality (14) trivially holds for $$\epsilon _0<\epsilon \le 1$$ whenever $$a_0\ge f(\epsilon _0)$$, it suffices to choose the constant threshold $$\epsilon _0\le \epsilon '_0(d,\gamma )$$ that would facilitate the induction step for all the smaller values $$\epsilon <\epsilon _0$$. To this end, plugging the desired induction assumption (14), with so far unknown $$a_0$$, into (13), we obtain

$$ f(\epsilon )\le \frac{a_0}{\epsilon ^{\alpha _d+\gamma }} \cdot J(\epsilon ), $$

where

$$ J(\epsilon )\le a_1\cdot \frac{1}{\log ^{\alpha _d+\gamma } r}+a_2\cdot \frac{s}{s^{(\alpha _d+\gamma )\left( \frac{1}{d-1}-\eta \right) }}+a_3\cdot \frac{t^{1+\eta }}{t^{(\alpha _d+\gamma )\left( \frac{1}{\alpha _d}-\eta \right) }}+ $$

$$ +a_4\cdot \frac{u^{1+\eta }}{u^{(\alpha _d+\gamma )\left( \frac{1}{d-1}-\eta \right) }}+a_5\cdot \frac{r^{\alpha _d+\eta }}{r^{(\alpha _d+\gamma )(1-\eta )}}+a_6\cdot \sum _{i=1}^l\frac{2^i w^{\alpha _d+\eta }}{2^{i\cdot (\alpha _d+\gamma )}w^{\alpha _d+\gamma }}+a_7\cdot \epsilon ^{\gamma -\eta }, $$

and $a_1,\ldots ,a_7$ are positive constants that do not depend on the choice of $\epsilon _0\le \epsilon '_0(d,\gamma )$ or $a_0$. Using that (i) *s*, *t*, *u*, *r* and *w* are fixed a positive powers of 1/ϵ, (ii) γ<1/100, and (iii) $\eta \le \gamma ^{2d}/100$, a sufficiently small choice of $0<\epsilon _0\le \epsilon '$ yields the inequality $J(\epsilon )\le 1$ for all $\epsilon \le \epsilon _0$. Hence, the bound (14) holds, with $a_0=f(\epsilon _0)$, for all $0<\epsilon \le 1$.

## Footnotes

1 The constant hidden within the O(·)-notation is specific to the family of geometric ranges under consideration, and is proportional to the VC-dimension of the induced hypergraph.

2 The preliminary version of this paper has claimed a slightly weaker bound in dimension d≥4, and misstated the bound for $f_3(\epsilon )$.

3 As a rule, the constants proportionality in our O(·) and $\Omega (\cdot )$ notation heavily depend on the dimension *d*, and the choice of γ>0.

4 According to the prevailing convention, the empty set is too considered a simplex whose dimension is -1.

5 Note that the implicit constant of proportionality in the lower bound $\Omega \left( h^{\beta _d}{n\atopwithdelims ()d+1}\right)$ cannot be improved beyond $(d+1)^{-(d+1)}$ [16] even in the special case h=1 (which yields the so called First Selection Theorem).

6 For the sake of brevity, we require that each partition encompasses exactly *r* sets $P_i$, for $1\le i\le r$, some of which can be empty.

7 This is done so as to differentiate between these simplices, which enclose the partition subsets $P_i$, on one hand, and the simplices of Pd on the other. Notice that the relative interiors of these cells $\Delta _i$ may overlap. Though a point of *P* may belong to several cells $\Delta _i$, for $1\le i\le r$, it is assigned to a *unique* cell $\Delta _i$ via the respective set $P_i$.

8 The running time was improved by Chan [20] to O(nlogn) (with high probability).

9 In the sequel we use logx denotes the standard binary logarithm $\log _2 x$.

10 The vertex *v* is unique given that no hyperplane in ${\mathcal {H}}$ is horizontal, which can be achieved by a slight generic rotation of the axis frame.

11 In the latter case, the dependence of our bounds on σ>0 will be explicitly spelled out.

12 Unless specified otherwise, in what follows we set *s* and other partition parameters to be arbitrary small, albeit fixed and positive, powers of 1/ϵ.

13 The earlier estimate $f_2(\epsilon )=O(1/\epsilon ^2)$ of Alon et al. [1] uses the essentially same argument with a *single line* (s=1) to derive the recurrence $f_2(\epsilon )\le 2\cdot f_2\left( 3\epsilon /2\right) +O(1/\epsilon ^2)$ in ϵ>0.

14 So far, we have only used the special case of Theorem 2.5, with h=1 (the so called *First Selection Theorem*). The reasons why we need the full generality of Theorem 2.5 (and even more than that) will become clear already in Sect. 3.

15 In Sect. 3 we refer to these categories of convex sets *K* as, respectively, ε*-narrow* and ε*-spread*.

16 In the sequel, *s* is chosen to be a very small, albeit fixed and positive, power of 1/ϵ. Matoušek and Wagner [45] used a similar divide and conquer argument, albeit using a *d*-dimensional simplicial partition; the resulting recurrence $f_d(\epsilon )=s\cdot f_d\left( \epsilon \cdot s^{1/d}\right) +s^{d^2}$ solved to $f_d(\epsilon )=O^*\left( 1/\epsilon ^d\right)$.

17 To be precise, such a partition $\mathcal {P}(P_i,t)$ will be constructed in Sect. 5 for each part $P_i$ of the vertical partition $\mathcal {V}(P,s)$ that underpins our small-size canonical family $\mathcal {{L}}=\mathcal {{L}}(P,s)$ of vertical lines. Given that *s* is an arbitrary small (albeit, fixed and positive) power of 1/ϵ, this will yield a simplicial $O^*(t)$-partition $\mathcal {P}(P,s,t)=\bigcup _{1\le i\le s}\mathcal {P}(P_i,t)$ of *P* whose properties resemble those described in Matoušek’s Theorem 2.6.

18 In particular, every “ε-light” convex set outside ${{\mathcal {K}}}(P,\epsilon )$ is ε-spread. However, most applications of the lemma involve convex sets *K* that are at least ε-heavy with respect to the underlying point set.

19 For the sake of brevity, we keep suppressing the constant multiplicative factors within the recursive terms of the general form $a\cdot f\left( \epsilon \cdot b\right)$, provided that these factors are much larger than 1/*b* and 1/*a*.

20 Though the image X=χ(V) is in general position, the map χ is not necessarily injective. Thus, some simplices f∈E could be mapped to lower-dimensional simplices $\chi (f)\in {X\atopwithdelims ()\le d}$.

21 Equivalently, the set *Y* is comprised of all the vertices in the (d-1)-dimensional arrangement of ${\mathcal {H}}\left( {X\atopwithdelims ()d}\right)$.

22 This is done so as to guarantee that the arguments in all the recursive terms $a \cdot f(\epsilon \cdot b)$, with $b=b(\epsilon )\gg \log 1/\epsilon$, significantly increase with each invocation of the recurrence.

23 Note that the first family, ${{\mathcal {K}}}_{>\delta }$, may include additional ϵ-heavy sets $K\in {{\mathcal {K}}}(P,\epsilon )$, which are not $(\epsilon ,\sigma )$-restricted to (P,Π). As a result, their respective nets will not depend on the subset $\Pi \subseteq {P\atopwithdelims ()d}$.

24 With some abuse of notation, $\mathcal {V}(P,s)$ is treated as the collection $\{P_1,\ldots ,P_{s}\}$ of its parts.

25 Up to an infinitesimally small symbolic perturbation [30] of the clipped cells $\varphi^{\prime }\left(p\right)$, it can be further assumed with no loss of generality that (i) the entire set $V^{\prime }\cup P$ is in general position, and that (ii) either $$V'(p)\cap V'(q)=\emptyset $$ or $$\varphi '(p)=\varphi '(q)$$ holds for any two points p,q∈P.

26 This is because no hyperplane H(τ) through a simplex $$\tau \in {P_K\atopwithdelims ()d}$$ can pass in a close vicinity of a point of $$X'_\ell $$, provided a generic enough position of *P* and $V^{\prime }$.

27 Notice that these *d* ambient cells $\varphi (p_i)$ must be distinct, as each of them comes from a distinct secondary partition $\mathcal {P}_i=\mathcal {P}(P_i,t)$.

28 A more general study of the space of tangent hyperplanes, due to Cappell et al. [19], implies that *any* family of *d* convex sets in $\mathbb {R}^d$, whose elements cannot be simultaneously intersected by a (d-2)-flat, must be “sandwiched” between a pair of distinct oriented external hyperplane tangents. The condition of Cappell et al. is clearly satisfied by $\{\varphi _1,\ldots ,\varphi _d\}$, due to the separation of $\{\varphi '_1,\ldots ,\varphi '_d\}$ in $\mathbb {R}^{d-1}$.

29 Notice that the described subdivision of $\mathcal {P}_0(K)$ is not necessarily disjoint due to the possible overlap between the families $\mathcal {I}_0(K)$ and $\mathcal {Z}_0(K)$.

30 In particular, this implies that the clipped cells $\varphi '(p_1),\ldots ,\varphi '(p_d)$ are distinct.

31 For the sake of our subsequent case analysis, it is immaterial whether the families $\mathcal {F}'_0(K)$ and $\mathcal {F}''_0(K)$ are contained in $\mathcal {T}_0(K)$, as long as they cover sufficiently many points of $P_0(K)$.

32 The somewhat weaker bound, than in case of (b), is because the ambient clipped cells $\varphi '(p_i)$ of the vertices $p_i$ of τ, for $1\le i\le d$, can be full and, thus, each contain up to $\lfloor \epsilon _1n\rfloor$ points of the respective subset $P_i(K)$.

## References

[1] Alon, N., Bárány, I., Füredi, Z., Kleitman, D.J.: Point selections and weak -nets for convex hulls. Comb. Prob. Comput. **1**, 189–200 (1992)

[2] Alon, N.: A non-linear lower bound for planar epsilon-nets. Discrete Comput. Geom. **47**, 235–244 (2012)

[3] Alon, N., Kalai, G.: Bounding the piercing number. Discrete Comput. Geom. **13**, 245–256 (1995)

[4] Alon, N., Kalai, G., Meshulam, R., Matoušek, J.: Transversal numbers for hypergraphs arising in geometry. Adv. Appl. Math. **29**, 79–101 (2001)

[5] Alon, N., Kaplan, H., Nivasch, G., Sharir, M., Smorodinsky, S.: Weak -nets and interval chains. J. ACM **55**(6), 28 (2008)

[6] Alon, N., Kleitman, D.J.: Piercing convex sets and the Hadwiger-Debrunner (p, q)-problem. Adv. Math. **96**(1), 103–112 (1992)

[7] Aronov, B., Ezra, E., Sharir, M.: Small-size epsilon nets for axis-parallel rectangles and boxes. SIAM J. Comput. **39**, 3248–3282 (2010)

[8] Aronov, B., Matoušek, J., Sharir, M.: On the sum of squares of cell complexities in hyperplane arrange- ments. J. Combin. Theory Ser. A. **65**, 311–321 (1994)

[9] Aronov, B., Sharir, M.: Cell complexities in hyperplane arrangements. Discrete Comput. Geom. **32**, 107–115 (2004)

[10] Aronov, B., Pellegrini, M., Sharir, M.: On the zone of a surface in a hyperplane arrangement. Discrete Comput. Geom. **9**(2), 177–186 (1993)

[11] Aronov, B., Miller, E., Sharir, M.: Eliminating depth cycles among triangles in three dimensions. Discrete Comput. Geom. **64**, 627–653 (2020)

[12] Balogh, J., Morris, R., Samotij, W.: Independent sets in hypergraphs. J. AMS **28**, 669–709 (2015)

[13] Balogh, J., Solymosi, J.: On the number of points in general position in the plane. Disc. Analysis **16**, 1–20 (2018)

[14] Bárány, I., Füredi, Z., Lovász, L.: On the number of halving planes. Discrete Comput. Geom. **10**(2), 175–183 (1990)

[15] Brönnimann, H., Goodrich, M.T.: Almost optimal set covers in finite VC-dimension. Discrete Comput. Geom. **14**(4), 463–479 (1995)

[16] Bukh, B., Matoušek, J., Nivasch, G.: Stabbing simplices by points and flats. Discrete Comput. Geom. **43**, 321–338 (2010)

[17] Bukh, B., Matousek, J., Nivasch, G.: Lower bounds for weak epsilon-nets and stair-convexity. Israel J. Math. **182**, 199–228 (2011)

[18] Bradford, P.G., Capoyleas, V.: Weak epsilon-nets for points on a hypersphere. Discrete Comput. Geom. **18**(1), 83–91 (1997)

[19] Cappell, S.E., Goodman, J.E., Pach, J., Pollack, R., Sharir, M., Wenger, R.: Common tangents and common transversals. Adv. Math. **106**(2), 198–215 (1994)

[20] Chan, T.M.: Optimal Partition Trees. Discrete Comput. Geom. **47**, 661–690 (2012)

[21] Chazelle, B.: Cutting hyperplane arrangements. Discrete Comput. Geom. **6**, 385–406 (1991)

[22] Chazelle, B., Welzl, E.: Quasi-optimal range searching in spaces of finite VC-dimension. Discrete Comput. Geom. **4**, 467–489 (1989)

[23] Chazelle, B.: The discrepancy method: randomness and complexity. Cambridge University Press, New York, NY, USA (2000)

[24] Chazelle, B., Edelsbrunner, H., Grigni, M., Guibas, L.J., Sharir, M., Welzl, E.: Improved bounds on weak epsilon-nets for convex sets, Discrete Comput. Geom. 13 (1995), 1–15. Also in Proc. 25th ACM Sympos. Theory Comput. (STOC), (1993)

[25] Chazelle, B., Friedman, J.: A deterministic view of random sampling and its use in geometry. Combinatorica **10**(3), 229–249 (1990)

[26] Clarkson, K.L., Edelsbrunner, H., Guibas, L.J., Sharir, M., Welzl, E.: Combinatorial complexity bounds for arrangement of curves and spheres. Discrete Comput. Geom. **5**, 99–160 (1990)

[27] Clarkson, K.L., Shor, P.: Applications of random sampling in computational geometry, II. Discrete Comput. Geom. **4**, 387–421 (1989)

[28] Clarkson, K.L., Varadarajan, K.R.: Improved approximation algorithms for geometric set cover. Discrete Comput. Geom. **37**(1), 430–58 (2007)

[29] Edelsbrunner, H., Guibas, L.J., Sharir, M.: The complexity of many cells in arrangements of planes and related problems. Discrete Comput. Geom. **5**, 197–216 (1990)

[30] Edelsbrunner, H., Mücke, E.: Simulation of simplicity: a technique to cope with degenerate cases in geometric algorithms. ACM Trans. Graph. **9**(1), 66–104 (1990)

[31] Eppstein, D.: Improved bounds for intersecting triangles and halving planes. J. Combin. Theory Ser. A **62**, 176–182 (1993)

[32] Even, G., Rawitz, D., Shahar, S.: Hitting sets when the VC-dimension is small. Inf. Process. Lett. **95**(2), 358–362 (2005)

[33] Furstenberg, H., Katznelson, Y.: A density version of the Hales-Jewett Theorem. J. Analyse Math. **57**(1991), 64–119 (1991)

[34] Hadwiger, H., Debrunner, H.: Über eine variante zum Hellyschen satz. Arch. Math. **8**(4), 309–313 (1957)

[35] Halperin, D., Sharir, M.: New bounds for lower envelopes in three dimensions, with applications to visbility in terrains. Discrete Comput. Geom. **12**, 313–326 (1994)

[36] Haussler, D., Welzl, E.: -nets and simplex range queries. Discrete Comput. Geom. **2**, 127–151 (1987)

[37] Holmsen, A.: Large cliques in hypergraphs with forbidden substructures. Comput. Geom., (2020). (to appear in Discrete)

[38] Holmsen, A., Lee, D.-G.: Radon numbers and the fractional Helly theorem, (2019). priprint arXiv:1903.01068

[39] Keller, C., Smorodinsky, S.: A New Lower Bound on Hadwiger-Debrunner Numbers in the Plane, in Proc. ACM-SIAM Symposium on Discrete Algorithms (SODA 2020), pp. 1155–1169 (2020)

[40] Keller, C., Smorodinsky, S., Tardos, G.: Improved bounds for Hadwiger-Debrunner numbers, Israel J. Math. 225 (2), 925 – 945. (2018). (Also in Proc. SODA 2017, pp. 2254 – 2263.)

[41] Kolmós, J., Pach, J., Woeginger, G.J.: Almost tight bounds for epsilon-Nets. Discrete Comput. Geom. **7**, 163–173 (1992)

[42] Matoušek, J.: Epsilon-Nets and Computational Geometry. In: Pach, J. (ed.) New Trends in Discrete Computational Geometry, pp. 69–89. Algorithms and Combinatorics, Berlin (1993)

[43] Matoušek, J.: Lectures on Discrete Geometry. Springer-Verlag, New York (2002)

[44] Matoušek, J.: Efficient partition trees. Discrete Comput. Geom. **8**(3), 315–334 (1992)

[45] Matousek, J., Wagner, U.: New constructions of weak epsilon-nets. Discrete Comput. Geom. **32**(2), 195–206 (2004)

[46] Matousek, J., Seidel, R., Welzl, E.: How to net a lot with little: small epsilon-nets for disks and halfspaces, Proc. 6th ACM Symp. Comput. Geom., (1990), pp. 16–22

[47] Moran, S., Yehudayoff, A.: On weak epsilon-nets and the Radon number, Proc. 35th Symp. Comput. Geom. (2019), pp. 51:1-51:14

[48] Mustafa, N.H., Ray, S.: Weak -nets have a basis of size . Comput. Geom. **40**, 84–91 (2008)

[49] Mustafa, N.H., Varadarajan, K.: Epsilon-approximations and epsilon-nets, Chapter 47 in Handbook of Discrete and Computational Geometry. Presented at the (2017). (**3rd edition**)

[50] Nivasch, G., Sharir, M.: Eppstein’s bound on intersecting triangles revisited. J. Comb. Theory, Ser. A **116**(2), 494–497 (2009)

[51] Pach, J., Tardos, G.: Tight lower bounds for the size of epsilon-nets. J. AMS **26**, 645–658 (2013)

[52] Pollack, R., Wenger, R.: Necessary and sufficient conditions for hyperplane transversals. Combinatorica **10**(3), 307–311 (1990)

[53] Rubin, N.: An improved bound for weak epsilon-nets in the plane, J. ACM (69) (5), Article 32, 35pp. Also in Proc FOCS (2018), pp. 224–235

[54] Rubin, N.: Improved bounds for point selections and halving hyperplanes in higher dimensions, in Proc. 2024 Annual ACM-SIAM Sympos. Discrete Algorithms (SODA 2024), pp. 4464–4561. Also in https://arxiv.org/abs/2403.00412.

[55] Tagansky, B.: A new technique for analyzing substructures in arrangements of piecewise linear surfaces. Discret. Comput. Geom. **16**(4), 455–479 (1996)

[56] Saxton, D., Thomason, A.: Hypergraph containers. Invent. Math. **201**(3), 925–992 (2015)

[57] Sharir, M.: Almost tight upper bounds for lower envelopes in higher dimensions. Discrete Comput. Geom. **12**, 327–345 (1994)

[58] Sharir, M., Agarwal, P.K.: Davenport-Schinzel Sequences and Their Geometric Applications. Cambridge University Press, New York (1995)

[59] Szemerédi, E., Trotter, W.T.: Extremal problems in discrete geometry. Combinatorica **3**(3–4), 381–392 (1983)

[60] Vapnik, V.N., Chervonenkis, A.Y.: On the uniform convergence of relative frequencies of events to their probabilities. Theory Prob. Appls. **16**, 264–280 (1971)

[61] Wenger, R.: Helly-type theorems and geometric transversals, in *Handbook of Discrete and Computational Geometry*, 2nd Edition (J.E. Goodman and J. O’Rourke, Eds.), Chapman & Hall/CRC Press, (2004), pp. 73–96

